# Integrative genetic and immune cell analysis of plasma proteins in healthy donors identifies novel associations involving primary immune deficiency genes

**DOI:** 10.1186/s13073-022-01032-y

**Published:** 2022-03-09

**Authors:** Barthelemy Caron, Etienne Patin, Maxime Rotival, Bruno Charbit, Matthew L. Albert, Lluis Quintana-Murci, Darragh Duffy, Antonio Rausell, the Milieu Intérieur Consortium, Laurent Abel, Andres Alcover, Hugues Aschard, Philippe Bousso, Nollaig Bourke, Petter Brodin, Pierre Bruhns, Nadine Cerf-Bensussan, Ana Cumano, Caroline Demangel, Christophe d’Enfert, Ludovic Deriano, Marie-Agnès Dillies, James Di Santo, Françoise Dromer, Gérard Eberl, Jost Enninga, Jacques Fellay, Ivo Gomperts-Boneca, Milena Hasan, Magnus Fontes, Gunilla Karlsson Hedestam, Serge Hercberg, Molly A. Ingersoll, Rose Anne Kenny, Olivier Lantz, Frédérique Michel, Hugo Mouquet, Cliona O’Farrelly, Etienne Patin, Sandra Pellegrini, Stanislas Pol, Antonio Rausell, Frédéric Rieux-Laucat, Lars Rogge, Anavaj Sakuntabhai, Olivier Schwartz, Benno Schwikowski, Spencer Shorte, Frédéric Tangy, Antoine Toubert, Mathilde Touvier, Marie-Noëlle Ungeheuer, Christophe Zimmer, Matthew L. Albert, Darragh Duffy, Lluis Quintana-Murci

**Affiliations:** 1 Université de Paris, INSERM UMR1163, Imagine Institute, Clinical Bioinformatics Laboratory, F-75006 Paris, France; 2 Human Evolutionary Genetics Unit, Institut Pasteur, UMR2000, CNRS, Université de Paris, F-75015 Paris, France; 3 Cytometry and Biomarkers UTechS, CRT, Institut Pasteur, Université de Paris, F-75015 Paris, France; 4 HIBIO, South San Francisco, CA 94080 USA; 5 Human Genomics and Evolution, Collège de France, F-75005 Paris, France; 6 Translational Immunology Unit, Institut Pasteur, Université de Paris, F-75015 Paris, France; 7 Service de Médecine Génomique des Maladies Rares, AP-HP, Necker Hospital for Sick Children, F-75015 Paris, France

**Keywords:** pQTL, Plasma proteins, Immune cells, Immune variability

## Abstract

**Background:**

Blood plasma proteins play an important role in immune defense against pathogens, including cytokine signaling, the complement system, and the acute-phase response. Recent large-scale studies have reported genetic (i.e., protein quantitative trait loci, pQTLs) and non-genetic factors, such as age and sex, as major determinants to inter-individual variability in immune response variation. However, the contribution of blood-cell composition to plasma protein heterogeneity has not been fully characterized and may act as a mediating factor in association studies.

**Methods:**

Here, we evaluated plasma protein levels from 400 unrelated healthy individuals of western European ancestry, who were stratified by sex and two decades of life (20–29 and 60–69 years), from the Milieu Intérieur cohort. We quantified 229 proteins by Luminex in a clinically certified laboratory and their levels of variation were analyzed together with 5.2 million single-nucleotide polymorphisms. With respect to non-genetic variables, we included 254 lifestyle and biochemical factors, as well as counts of seven circulating immune cell populations measured by hemogram and standardized flow cytometry.

**Results:**

Collectively, we found 152 significant associations involving 49 proteins and 20 non-genetic variables. Consistent with previous studies, age and sex showed a global, pervasive impact on plasma protein heterogeneity, while body mass index and other health status variables were among the non-genetic factors with the highest number of associations. After controlling for these covariates, we identified 100 and 12 pQTLs acting in *cis* and *trans*, respectively, collectively associated with 87 plasma proteins and including 19 novel genetic associations. Genetic factors explained the largest fraction of the variability of plasma protein levels, as compared to non-genetic factors. In addition, blood-cell fractions, including leukocytes, lymphocytes, monocytes, neutrophils, eosinophils, basophils, and platelets, had a larger contribution to inter-individual variability than age and sex and appeared as confounders of specific genetic associations. Finally, we identified new genetic associations with plasma protein levels of five monogenic Mendelian disease genes including two primary immunodeficiency genes (Ficolin-3 and FAS).

**Conclusions:**

Our study identified novel genetic and non-genetic factors associated to plasma protein levels which may inform health status and disease management.

**Supplementary Information:**

The online version contains supplementary material available at 10.1186/s13073-022-01032-y.

## Background

Plasma proteins play important physiological roles in human health and disease. They participate in immune responses against pathogens (e.g., interferons, chemokines, and complement factors [1, 2]), blood clotting [3], hormone transport [4, 5], and energy metabolism regulation [6]. Plasma protein levels can reflect physiological homeostasis or pathological states including active cellular secretion [7–9], tissue leakage [10, 11], protein degradation [12], and protein excretion in urine [13]. Plasma proteins are widely used as markers of the physiological state of an individual and represent ~42% of all requested blood-based laboratory tests [10]. As of today, the US Food and Drug Agency (FDA) approved 235 plasma proteins as diagnostic, prognostic, risk predictive, or treatment response biomarkers [14, 15]) for a broad range of diseases such as cancer [16–18], pulmonary defects [19], autoimmune [20], and metabolic diseases [21]. In addition to their association with clinical outcomes, natural heterogeneity of plasma protein levels among the general population has been widely reported but is not considered in clinical applications. Recent large-scale studies performed both in healthy and disease cohorts have identified both non-genetic (e.g., age and sex) and genetic factors (i.e., protein quantitative trait loci, pQTLs) that determine variable plasma protein levels [22–24]. pQTLs are enriched in disease-susceptibility loci identified from GWAS studies [24, 25] and could have protective or modifying effects, potentially in conjunction with pathogenic mutations leading to disease due to altered expression levels, e.g., loss of homeostasis, proteotoxic stress, or insufficiency [26]. Yet, the assessment of the genetic associations reported by previous studies did not characterize the specific cell types accounting for the observed variation in plasma proteins. Thus, it remains unclear whether a fraction of the plasma protein variability initially associated with a pQTL could have been mediated by the concomitant heterogeneity in their cellular sources. This may be especially relevant for plasma proteins displaying immune-related functions, since significant variability in immune cell fractions is observed across individuals driven by both genetic and non-genetic factors [27–29].

Here, we present an in-depth characterization of heterogeneity in plasma protein levels in healthy individuals from the Milieu Intérieur study [30], with a focus on immune-related proteins. The Milieu Intérieur consortium aims at characterizing the genetic and environmental factors underlying the observed variability of the immune response in a healthy population [30]. This study was performed on 400 individuals equally distributed by sex and across two decades of life (aged 30–39 and 60–69). We evaluated the association of 229 plasma protein concentrations with a total of 254 non-genetic factors including lifestyle, environmental, physiological, and blood biochemical variables as well as with 5,201,092 common single-nucleotide polymorphisms (SNPs). To control for the natural variation in blood-cell populations, we systematically accounted for the levels of seven major blood-cell fractions, including leukocytes, lymphocytes, monocytes, neutrophils, eosinophils, basophils, and platelets. We found that together with age and sex, blood-cell fractions explain an important fraction of the inter-individual plasma protein variability. After controlling for such factors, we identified 112 pQTLs associated with 87 proteins, 19 of which are reported here for the first time. Among these, six are associated with five monogenic Mendelian disease genes (MMDGs), including 2 primary immunodeficiency (PID) genes. Such genetic variants may have potential clinical value as susceptibility or protective factors for immune-related diseases.

## Methods

### The Milieu Intérieur cohort

The 400 donors in this study were a subset of the 1000 healthy donors of the Milieu Intérieur cohort [27, 29–32] recruited at BioTrial (Rennes, France). The Milieur Intérieur cohort was approved by the Comité de Protection des Personnes – Ouest 6 (Committee for the protection of persons) on June 13, 2012 and by French Agence nationale de sécurité du médicament (ANSM) on June 22, 2012. The study is sponsored by Institut Pasteur (Pasteur ID-RCB Number: 2012-A00238-35) and was conducted as a single-center interventional study without an investigational product. The original protocol was registered under ClinicalTrials.gov (study # NCT01699893). The samples and data used in this study were formally established as the Milieu Interieur biocollection (NCT03905993), with approvals by the Comité de Protection des Personnes – Sud Méditerranée and the Commission nationale de l'informatique et des libertés (CNIL) on April 11, 2018. Donors included in this sub-study were stratified by sex and were between the ages of 30–39 (*n* = 200) or 60–69 (*n* = 200) years old. Participants were selected based on stringent inclusion and exclusion criteria, as detailed elsewhere [30]. To minimize the influence of population substructure on genomic analyses, the study was restricted to individuals of self-reported Metropolitan French origin for three generations (i.e., with parents and grand-parents born in continental France). Fasting whole blood samples were collected in EDTA tubes, and plasma was separated following high-speed centrifugation and stored at – 80 °C until analysis. Standard blood testing and complete hemogram was performed on fresh aliquots, while protein immunoassays were performed on frozen aliquots.

### Quantification of plasma protein levels in 400 healthy individuals

The protein immunoassays and the blood tests were performed on samples taken the same day and analyzed at different times, on frozen and fresh aliquots, respectively. Blood chemical and major cell fractions were estimated through direct enumeration and standard blood panels. The concentrations of 297 plasma proteins of 400 individuals were quantified by Luminex multi-analyte immunoassays (Discovery Map v3.3 from Myriad RBM, Additional file 1: Table S1), as previously described [33]. Proteins measured included cytokines, chemokines, metabolic markers, hormones, growth factors, tissue remodeling proteins, angiogenesis markers, acute-phase reactants, cancer markers, kidney damage markers, and central nervous system (CNS) biomarkers. Protein levels were analyzed and compared with their respective lower limit of quantification (LLOQ). Among the 297 assayed proteins, 68 proteins were reported at a concentration lower than the LLOQ in at least 20% of the individuals and were filtered out. For the 229 proteins that were kept, reported concentrations lower than the LLOQ were considered as missing values (NAs), to prevent incorrect protein-environment or protein-genotype associations due to undetected or undetectable proteins. Next, for each protein, plasma-level distributions across individuals were tested for normality using the Shapiro-Wilk test on the raw and log-transformed values within the 2.5% and 97.5% percentiles. Shapiro-Wilk null hypothesis was not rejected (*p*-value ≤ 0.001, after multiple testing correction) for a total of 50 (22%) and 183 (80%) proteins, depending on whether raw or log-transformed values were used. These results suggested that the majority of raw protein plasma levels followed a log-normal distribution. Thus, the levels of all 229 proteins were log-transformed for downstream analyses.

### Filters and tests for non-genetic variables

For each individual from the Milieu Intérieur cohort, an extensive electronic clinical record file was filled, gathering 754 lifestyle, environmental, and medical history variables as well as blood metabolite and enzyme levels from standard blood test and erythrocyte enumeration [30]. First, variables with names describing repetitive measurements over several visits after the first visit were filtered out. Second, redundant columns informative about the sex of the individual were removed. Third, mono-factorial variables, character variables, variables with missing values in 20% or more of the individuals, or varying in less than 10 individuals, variables correlated with other variables with a Spearman correlation coefficient of 1, and variables providing redundant information about the same phenotype were filtered out, for a final number of 254 variables. Then, for each of the 254 non-genetic factors, a univariate linear regression analysis was performed against the log-transformed expression levels of each of the 229 plasma proteins evaluated. Age and sex were systematically included as covariates in all such regressions, consistent with their pervasive influence shown in previous studies, as well as with their association with many of the non-genetic factors evaluated [30, 34]. Univariate linear regression was performed between each pair of proteins and non-genetic factors. In addition, to reduce the sensitivity of the linear models to outliers, the ten lowest and ten highest values of each protein were removed from the regression analysis. Significance was declared at *p*-value ≤ 0.05 after Bonferroni multiple testing correction accounting for the number of tests (*n* = 58,166). A total of 152 significant associations collectively involving 49 proteins and 20 non-genetic factors were found. In addition, seven major blood-cell fractions, i.e., leukocytes, lymphocytes, monocytes, neutrophils, eosinophils, basophils, and platelets, were assessed through hemogram on fresh aliquots, along the other blood chemicals and enzymes [30].

### Genotyping and imputation

Each individual from the Milieu Intérieur cohort was genotyped by the HumanOmniExpress-24 BeadChip (Illumina), covering 719,665 SNPs. In total, 245,766 rare functional variants were also genotyped on a HumanExome-12 BeadChip (Illumina). After quality control, both datasets were merged, for a total of 723,341 SNPs, all mapped in GRCh37.p13 coordinates. Next, IMPUTE v.2 [35] was used to perform genotype imputation, on 1-Mb windows buffered by an additional 1 Mb. Before imputation, SNPs were phased using 500 conditioning haplotypes, 50 MCMC, 10 burn-in, and 10 pruning iterations. SNPs and allelic states were aligned to the imputation reference panel from the 1000 Genome Project Phase 1 v3 (2010/11/23). SNPs with dissimilar alleles (even after flipping) or ambiguous C/G or A/T alleles were filtered out. Imputation yielded a total of set of 37,895,612 SNPs. Removing SNPs with information metric ≤ 0.8, duplicated or monomorphic SNPs, and SNPs with missingness > 5% (SNPs with genotyping probability lower than 0.8 in an individual were considered as missing) reduced the set to 11,395,554 SNPs. Further removing non-SNP variants and filtering out variants with a MAF < 0.05 (with the --snps-only option of PLINK v1.9) in the 400 sampled individuals resulted in a final set of 5,201,092 SNPs. Following Patin et al [29], principal component analysis (PCA) of the OmniExpress array was performed on 261,827 independent SNPs with 36 reference populations from north and west Africa, Middle East, western Asia, and Europe (Human Genome Diversity Panel [36]) and the principal components (PCs) explaining more than 1 % of the total variance were kept to account for potential population stratification (PC1 = 5.42%, PC2 = 1.63%).

### Genome-wide association testing of plasma proteins

To perform the pQTL mapping of plasma proteins, we chose to use a multivariate approach by incorporating, for each protein, the associated non-genetic variables as covariates, in addition to sex, age, the 7 major blood-cell fractions (leukocytes, lymphocytes, monocytes, neutrophils, eosinophils, basophils, and platelets), and the two first PCs of the genetic data. If a non-genetic variable was a redundant measure with the corresponding protein (i.e., measured by both Luminex and standard blood panel, e.g., CRP), it was not added as a covariable in the model. We used a first linear mixed model to correct the protein expression levels for their specific covariates and for kinship (mean identity by state, a.k.a. IBS = − 0.0025 ± 0.026 over each pair of individuals, with two pairs of individuals with IBS = 0.2 and IBS = 0.3, potentially indicating second-degree relatives), using per chromosome genetic relationship matrix (GRM) computed using GenAbel v1.8 [37] (leaving one chromosome out). In order to limit the correction for multiple testing while still accounting for both the number of tested SNPs and proteins, the analysis was performed separately for *cis* and *trans* QTLs, and the false discovery rate (FDR) was computed independently for *cis*-acting and *trans*-acting SNPs, following Quach et al. [38]. *Cis*-acting SNPs were defined as SNPs located at a maximum distance of 1 MB from the transcription start or end site of the corresponding gene, while all other SNPs are defined as *trans-*acting. For each protein and each kind of associations tested (*cis* or *trans*), the minimal raw *p*-value was reported. In addition, for each protein, 100 permutations were performed between all *cis-* or *trans*-SNPs, and the minimal *p*-value of each of these permutations was extracted. Next, proteins were ascendingly sorted based on their raw *p*-values. Then, the FDR was computed, for each protein, as the mean over the *N* = 100 permutations of the number of times its raw *p*-value is lower than the *n*th permutation from all proteins, divided by the rank of the corresponding protein. Protein-SNP pairs were considered as significant when the corresponding FDR was equal to or lower than 0.05, for a total of 78 cis- and 22 trans-pQTLs. To investigate the potential presence of secondary pQTLs, we performed the same analysis a second time, incorporating the genotype of the most significant SNP detected in the first round of analysis as an additional covariate. The FDR was computed independently on each analysis iteration, both considering the same number of proteins and permutations. The conditional analysis yielded 11 cis- and 1 trans-pQTLs. The significance thresholds corresponding to first-round and second-round pQTLs were respectively around 2.2e−05 and 1.5e−05 for *cis* and respectively around 9e−10 and 2e−10 for *trans* (significance thresholds were computed as the mean between the last significant and the first non-significant *p*-values). The genome-wide analysis yielded a total of 100 *cis*-pQTLs and 12 *trans*-pQTLs.

### Contribution of non-genetic and genetic components in the variability of plasma proteins

The relative contribution of the various environmental and genetic variables was assessed using the correlation-adjusted marginal correlation score (CAR score [39]) from the care package in R. The CAR score is the shrunk estimator of the adjusted coefficient of determination (*R*^2^) of each independent variable in a linear model, which considers the marginal correlation between variables. The CAR score is determined for each independent variable within a model, representing their independent contribution to the total variability of the dependent variable. The sum of the CAR score attributed to each variable in a model is equal to the model adjusted *R*^2^. For each protein, the relative contribution of its significantly associated non-genetic variable was assessed at once. In case only a single variable was significantly associated with a protein, we used the adjusted coefficient of determination (*R*^2^) of the variable as its relative contribution in the variability of the corresponding protein levels. Then, the relative contribution of the identified pQTLs was assessed by computing their CAR score in protein-specific models incorporating age, sex, the protein-specific covariates, and the corresponding genotypes. The effect size of pQTLs was computed following a two-stage model similar to the GWAS. A first linear model was used to regress out the associated covariates (and previously identified SNP in the case of SNPs identified by the conditional analysis) from the log-transformed and non-transformed plasma protein levels, and a second linear model was used to regress the residuals of the first against the tested SNP. The Beta was extracted from this model and used as the SNP effect size.

### Global impact of age and sex on plasma protein levels

In order to characterize the global impact of age and sex on plasma protein heterogeneity, while accounting for the collinearity of several plasma proteins, we performed a principal component analysis (PCA) on the expression levels of the 229 proteins across the 400 individuals. When considered independently, only PC1 and PC2 explained more than 5% of the total variability (Additional file 2: Figure S1).

### Assessment of gene-gene interactions

The interactions between *trans*-pQTL associated proteins and candidate proteins coded by genes located within a 500-kb window centered around the associated SNP were assessed using STRING-db v11 [40] at https://string-db.org/. All protein-coding genes were queried at once through the “Multiple proteins” option and default settings (organism: “Homo sapiens”). Proteins were considered to interact when they were shown to be direct neighbors in protein-protein interaction networks, or when one of the protein was shown to be directly or indirectly involved in the regulation of the other.

### Contribution of blood-cell fractions in protein level predictions

To quantify the relevance of blood-cell fractions in the prediction of plasma protein levels, we used a one-way ANOVA to compare, for each pQTL, the predictions coming from two models. A first linear model considered the genotype of the corresponding SNP, the previously defined protein-specific covariates, and age, sex, the two first PCs of the genetic data, and the 7 blood-cell fractions. A second linear model was evaluated by considering all variables used in the previous one, with the exception of the 7 blood-cell fractions. The models including pQTLs obtained from the conditional analysis additionally corrected for the SNP used for their identification. To assess the potential relevance of the different circulatory cell fractions in the different results obtained from the two pQTL mapping, the proteins were first corrected for age and sex through linear regression, and the resulting residuals regressed against each circulatory cell counts individually. Association *p*-values were corrected independently for each protein and are reported in Additional file 3: Table S2.

### Genome-wide analysis of plasma proteins excluding blood-cell fractions

For the purpose of evaluating the contribution of blood-cell fractions in the pQTL assessment, the genome-wise association analysis was performed as previously detailed, while removing the 7 blood-cell fractions from the model (“Methods”). This approach identified 115 protein quantitative trait loci (pQTLs) collectively involving 94 proteins and 113 SNPs (FDR ≤ 0.05). Among them, 103 were defined as *cis*-pQTLs. In addition, 12 pQTLs were identified in *trans*, i.e., located further than 1 MB far from the gene boundaries, or located on another chromosome. In total, 73 proteins were associated with only one SNP, while 21 were associated with two independent SNPs. Among the 94 proteins with significant pQTLs, 83 proteins were associated exclusively with *cis*-pQTLs, 9 exclusively with *trans*-pQTLs, and 2 with both *cis* and *trans*-pQTLs. In comparison with the first analysis, 92 pQTLs were reproduced, 81 in *cis*, and 11 in *trans*. An association was considered as reproduced when the SNP, or a SNP in linkage disequilibrium with R2 ≥ 0.8, was significantly associated with the same protein at FDR ≤ 0.05. Twelve *cis*-pQTLs were no longer associating with the same SNPs or to SNPs in high LD (R2 ≥ 0.8) with it, but with other *cis*-SNPs, and 8 pQTLs (7 *cis*, 1 *trans*) were not reproduced. On the opposite, 10 additional *cis*- and 1 *trans*- pQTLs were obtained.

### Co-localization of trans-pQTLs and trans-eQTLs

We assessed the co-localization between the *trans*-pQTLs identified in this work and previously identified *trans*-eQTLs associated with the same gene and reported in the GWAS Catalog V1.0.2 [41], in eQTLs from GTEx V8 or in QTLbase v1.2 [42] (http://mulinlab.org/qtlbase) through the VannoPortal [43], using a LD threshold of *R*^2^ ≥ 0.8 (computed in Europeans from the 1000 Genome Project) between each pQTL and all SNPs present in a 200-kb window centered on the pQTL.

### Replication of previously identified plasma protein QTLs

We compared our significant SNP-protein pairs with four studies analyzing the genetic basis of plasma protein levels. Sun et al. [22] reported 1927 pQTLs, resulting from the analysis of 3622 plasma proteins in 3301 individuals; Suhre et al. [23] reported 539 pQTLs from the analysis of 1124 plasma proteins in 1000 individuals; Deming et al. [24] reported 56 pQTLs from the analysis of 146 plasma proteins in 818 individuals; and Zhong et al. [44] reported 144 pQTLs from the analysis of 107 plasma proteins in 101 individuals. The replication of the reported pQTLs was performed at the protein level rather than at the SNP level, due to the poor overlap in terms of sentinel SNPs reported in previous studies [22–24, 44] and our imputed set of 5,201,092 SNPs. A protein previously reported as cis-regulated (i.e., reported as significantly associated with a SNP annotated as “cis” by the authors) was considered replicated when it was significantly associated with a SNP located closer than 1 Mb around the gene extremities.

Overall, *cis*- and *trans*-pQTLs were labeled as novel when they were absent from the four reference studies used for replication, or when they—or SNPs in high LD with them (*R*^2^ ≥ 0.8, based on the reference European population from the 1000 Genome Project)—were absent from the GWAS catalog [41] and from QTLbase v1.2 [42] (http://mulinlab.org/qtlbase). The studies that previously identified the pQTLs reported in this work are referenced in Additional file 4: Table S3.

### Protein and gene annotations

Proteins were classified as immune-related when they were either (i) annotated as adaptive immune proteins (Table S2 from Fischer & Rausell, 2016 [45]), or innate immune proteins (Table S1 from Fischer & Rausell, 2016 [45], and Table S1 from Deschamps et al. [46]) or (ii) produced in sufficient concentrations in substantial fractions of immune cells, as described in Rausell et al. [47]. The list of primary immunodeficiency genes was obtained from Table S3 from Fischer & Rausell [45]. Gene-disease annotations were obtained from OMIM (downloaded at https://www.omim.org/downloads/ on the 2019/06/10). Entries were parsed following Caron et al. [48]. Mendelian disease genes were selected for their level of supporting evidence equal to 3 and for not having a “somatic” flag, and monogenic Mendelian disease genes (MMDGs) were further selected for not being flagged as “complex”. The list of secreted proteins was downloaded from UniProt (https://www.uniprot.org/) on the 2020/02/03, using the keywords: locations:(location:"Secreted [SL-0243]" type:component) AND organism:"Homo sapiens (Human) [9606]". The list of FDA-approved targeted proteins was downloaded on the 2020/02/05 from http://mrmassaydb.proteincentre.com/fdaassay/.

The protein classes were taken from Myriad RBM Discovery Map V3.3 table. Proteins were considered enriched or depleted in a specific class when the proportion of proteins in that class was larger than in 10,000 randomly sampled set of proteins of the same size, coming from the tested set of 229 proteins. Previous reports of pQTLs associated with monogenic Mendelian disease genes, with primary immunodeficiency genes or with genes coding for FDA-approved biomarkers were identified through QTLbase [42] (http://mulinlab.org/qtlbase).

### Disease loci enrichment

To assess the potential association with diseases or other traits of the *cis* and *trans*-pQTLs reported in this work was assessed, we used hits from the NHGRI-EBI Genome Wide Association Studies (GWAS) Catalog [41], downloaded on the 2019/03/22 (file name gwas_catalog_v1.0-associations_e95_r2019-03-22.tsv). SNPs associated with a trait or a disease with a reported *p*-value ≤ 1e−08 and mapped to autosomes were kept. SNPs associated with traits containing “blood,” “plasma” or “serum,” and “protein” were removed. A set of pQTLs was declared as enriched when the proportion of pQTLs in a set that were GWAS SNPs or in linkage disequilibrium with GWAS SNPs (*r*^2^ ≥ 0.8) was larger in 95% of 10,000 randomly sampled set of SNPs of the same size, matched by MAF (bins of 5%). Randomly sampled SNPs were drawn from 122,757 and 384,897 SNPs, selected respectively from the 1,674,134 and 5,201,092 SNPs tested for *cis* and *trans* associations respectively (with the --indep-pairwise 100 5 0.5 function of PLINK v1.9). When several traits or diseases were associated with one locus, the most significant one was selected.

### Functional annotation of pQTLs

The *cis*-pQTL molecular consequences were assessed using VEP v97 [49]. Each *cis*-pQTL was annotated based on the canonical transcript of the gene coding [50] for the regulated protein.

## Results

### Variation of plasma protein levels in a well-defined healthy population

**Fig. 1:**
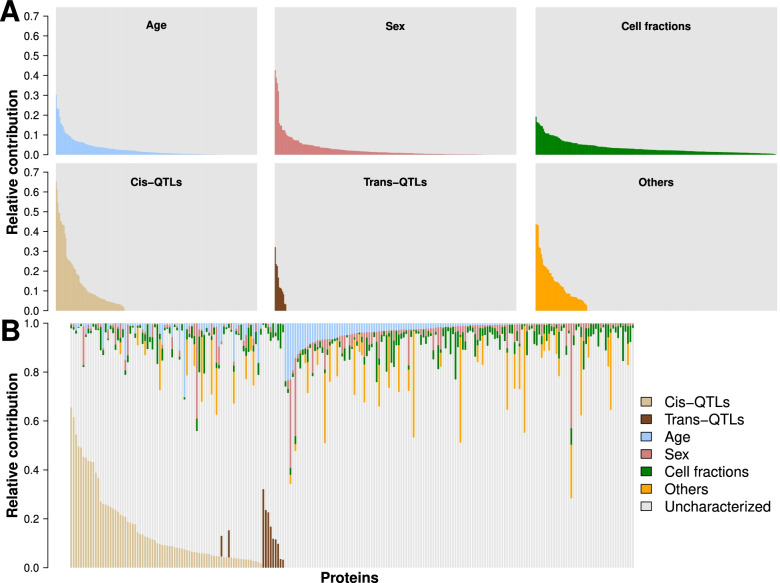
Contribution of environmental and genetic factors to the variability of plasma protein levels. The variability explained by the associated genetic and non-genetic factors in the levels of 229 plasma protein levels, taken independently (**A**) or altogether (**B**). Each vertical bar represents the total variability of a protein, with the contribution of the considered (colored) or other and unknown (gray) factors summing up to 1. Age (blue), sex (brick-red), *cis* (light brown), and *trans* (dark brown) pQTLs correspond directly to the assessed relative importance, the cell fraction category (green) represents the cumulated relative importance of lymphocytes, leukocytes, neutrophils, eosinophils, basophils, monocytes, and platelets, and the “Others” category (orange) represent the sum of the relative importance all other non-represented variables. In **A**, the gray area represents 1 minus the sum of all non-considered and unknown factors. In **B**, the “Uncharacterized” category was computed as 1 minus the sum of all other variables or groups of variables

We quantified the concentrations of 297 plasma proteins in 400 healthy individuals from the Milieu Intérieur (MI) study [30] using CLIA certified assays (Additional file 1: Table S1). After quality control accounting for detection limits and missing data (“Methods”), 229 proteins were retained for downstream analyses, including 141 immune-related plasma proteins (i.e., proteins with previously identified immune functions or produced by immune cells, “Methods”). First, we evaluated the impact of individuals’ genetics and non-genetic factors on plasma protein levels. Genome-wide association tests for each of the 229 proteins were performed against a total of 5,201,092 common SNPs (minor allele frequency ≥ 0.05). Covariates that we systematically included in the analysis were age, sex, counts of 7 major blood-cell sub-populations (lymphocytes, leucocytes, neutrophils, basophils, eosinophils, monocytes, and platelets), and the first two principal components of a principal component analysis of the genetic data, representing genetic stratification in the sample (“Methods”). Additional non-genetic factors were selected among 254 lifestyle, environmental, physiological, and blood biochemical variables and added as confounders in a protein-specific manner, based on their significant association with each protein (“Methods”). The relative contribution (marginal correlation, CAR score, “Methods”) of non-genetic, genetic, and cell fraction components to the inter-individual variability of the 229 plasma proteins evaluated in this work is presented in Fig. 1 and Additional file 5: Table S4, Additional file 6: Table S5, Additional file 7: Table S6 (“Methods”).

Consistent with previous studies [22–24, 44, 51–53], we found that age and sex had a widespread effect on plasma protein levels, each explaining on average 2.8% of the total observed variability (Fig. 1, Additional file 5: Table S4, Methods). Similar figures were observed for the subset of immune-related proteins, i.e., 2.3% and 2.1% for age and sex respectively. For specific proteins, however, the observed contribution of age and sex was particularly large, in line with previous findings. For example, age explained 30.2% of growth differentiation factor 15 variability (GDF15) [51, 54], while variability attributed to sex was 42.8% for Leptin [55], 39% for Stromelysin-1 (MMP3) [51, 56], 36.2% for FSH, and 32.1% for LH [51, 57]. Moreover, when accounting for potential covariation among the 229 proteins through a principal component analysis (PCA), both age and sex showed a strong association with the global heterogeneity of protein levels (univariate linear modeling against PC1 coordinates, *p*-values = 1.8e−07 and 1.4e−06, respectively; and PC2, *p*-values = 4.2e−24 and 3.5e−05, respectively; Additional file 2: Figure S1, “Methods”). While previous studies mostly assessed the global impact of age and sex on plasma proteins, their effects appear highly heterogeneous across proteins.

### Blood-cell fractions explain a substantial part of plasma protein-level heterogeneity

To assess the potential effect of circulatory cell counts on plasma protein levels, we next quantified their relative contribution to the inter-individual variability of each protein (“Methods”). Taken together, blood-cell fractions explained on average 3.6% of the variability of the observed plasma protein levels. This contribution was comparatively higher than those of age and sex (two-sided Wilcoxon test *p*-value = 6.4e−11 and 2.3e−14, respectively; Fig. 1, Additional file 5: Table S4). Furthermore, blood-cell fractions explained significantly more variability for immune-related proteins than for the rest of proteins evaluated (mean explained variability 4% and 2.9% respectively, one-sided Wilcoxon test, *P* = 4.8e−02). Platelet counts alone explained an average of 1.6% of the variability of immune-related proteins, as compared to 0.78% for the rest of proteins (one-sided Wilcoxon test, *p*-value = 3.9e−04), with contributions as high as 16.3% for the Neutrophil Activating Peptide 2 and 13.3% for Thrombospondin-1. These results highlight the contribution of blood-cell fractions to the variability of plasma protein levels and support their consideration as a covariate in the assessment of genetic associations.

### Plasma lipids and body mass index are important covariates of specific plasma proteins

**Fig. 2:**
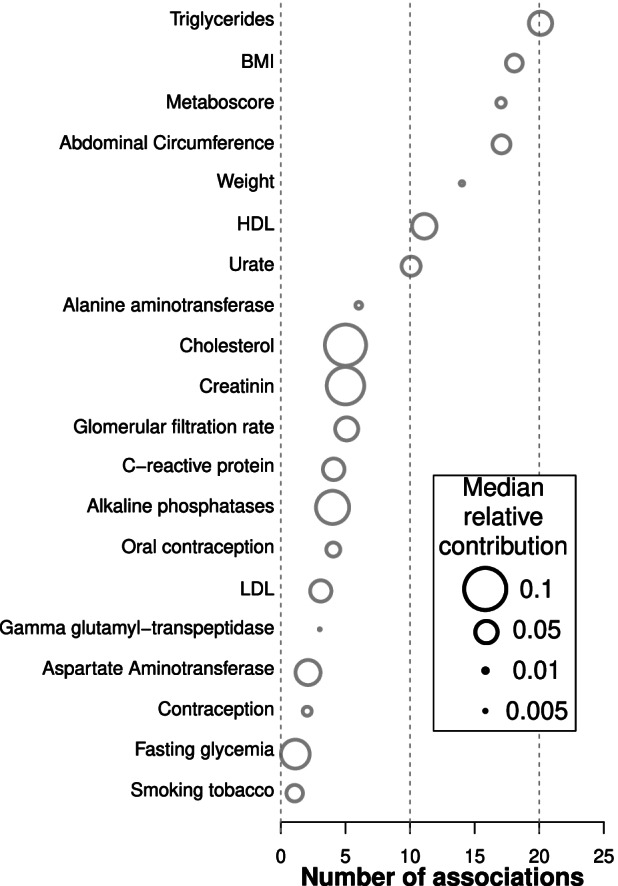
Relative contribution of selected factors in the variability of plasma protein levels. Relative contribution (CAR score [39]) of the 20 significantly associated factors to the variability of plasma protein levels. Variables are sorted depending on the number of significant associations with proteins. The number of proteins significantly associated with each variable is reported on the *x*-axis. The diameter of each dot represents the median CAR score of the corresponding factor in the variation of the associated plasma proteins

Two other classes of non-genetic factors were found to substantially associate with plasma levels of specific proteins. First, plasma lipids such as triglycerides, HDL, LDL, and total cholesterol were associated with expression levels of 25 proteins, including various components of cholesterol particles as well as proteins involved in lipid transport (ApoA1, ApoB, ApoC1, ApoC3, ApoD, ApoE, FABP-adipocyte, SHBG), metabolism, and homeostasis (Adiponectin, Carboxypeptidase B2, C3, CFH, C-peptid, Endoglin, FGF21, IGFBP2, Leptin, Leptin Receptor, PEDF, Prostatin, PSAT, RBP4, SAP, tPA). Second, anthropometric factors such as body mass index (BMI) and abdominal circumference were associated with plasma levels of 20 proteins, most of which also associate with plasma lipids (e.g., Adiponectin, ApoD, C-peptid, FABP-adipocyte, SHBG). Blood lipids and anthropometric factors accounted on average for 11% and 8.1% of the variability of the associated plasma proteins, respectively. Yet, the highest association was observed between HDL and the Apolipoprotein A-1 (marginal correlation of 44.6%) [58] (Figs. 1, 2, Additional file 6: Table S5, “Methods”). Globally, anthropometric factors and plasma lipid levels are known to be markers of physical shape and overall health. Interestingly, the association of complement factors (C3 and CFH) with anthropometric traits may reflect the low level inflammation induced by higher body mass, both of which associate with obesity, cardiovascular diseases, and increased susceptibility to infections [59–61].

### Contribution of human genetics on plasma protein levels

**Fig. 3:**
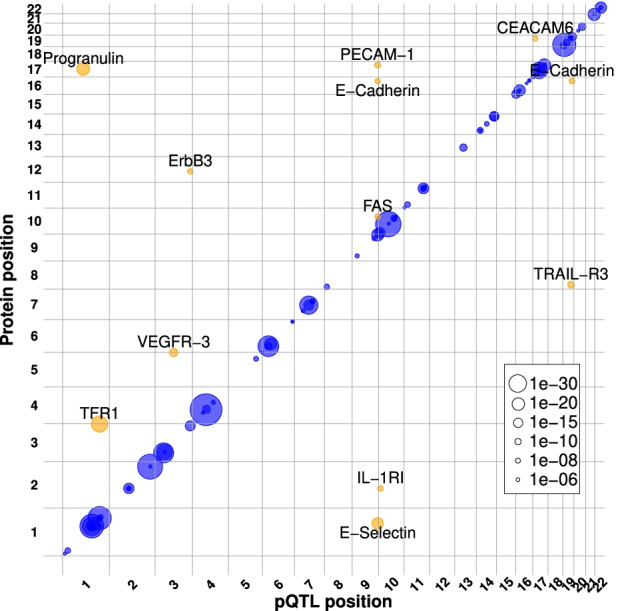
Plasma protein QTLs localization and co-regulation. The genomic positions of the *cis* (blue) and *trans* (orange) pQTLs identified in this work (*x*-axis) and the genomic location of the gene coding for the associated protein (*y*-axis). The point size is proportional to the uncorrected association *p*-value as reflected in the legend

Genome-wide association testing against the 229 plasma proteins identified 112 pQTLs, including 100 *cis*- and 12 *trans*-pQTLs, and collectively involving 87 proteins and 111 SNPs (FDR ≤ 0.05; Fig. 3, Additional file 2: Figure S2, Additional file 2: Figure S3, and Additional file 7: Table S6, Methods). Among the 87 proteins, 76 were only associated with *cis*-SNPs, 9 with only *trans*-SNPs, and 2 with both. Sixty-two proteins were associated with one SNP, and 25 with two independent SNPs. Interestingly, three loci aggregated several pQTLs associated with different proteins. First, among the 12 *trans*-pQTLs identified, 4 (E-selectin-rs2519093; PECAM-1-rs2519093; Cadherin-1-rs635634; FASLG receptor-rs687621) were in moderate (*R*^2^ = 0.45 rs2519093 and rs687621) to high (*R*^2^ = 0.99, rs2519093 and rs635634) linkage disequilibrium (LD) and co-localized in a 18-kb region of chromosome 9, previously described as the ABO locus, and known to be associated with the expression of many plasma proteins [23, 25, 62, 63]. Second, two SNPs on chromosome 1 in high LD (*R*^2^ ≥ 0.99) at the *CFHR4* locus associated, respectively, in *cis* (rs60642321) with CFHR1 plasma levels, and in *trans* (rs115094736) with TFR1 plasma levels. While the former had previously been reported in blood [64], the latter was, to the best of our knowledge, not reported before, neither as a pQTL nor as an eQTL. In addition, no physical or regulatory interactions have been reported between *TFRC* and any other gene or protein in a 500-kb window centered on the associated SNP (as reported in STRING-db [40], “Methods”). Last, two SNPs (rs584007 and rs3826688) in high LD (*R*^2^ ≥ 0.99), located on chromosome 19, associated in *cis* with the plasma levels of Apo E and Apo C1, respectively. Both SNPs are located within a known *ApoE* enhancer [65] and were previously described as *cis*-eQTLs of both genes (in blood or in other tissues), hinting at a potential co-regulation of the expression of both genes [66, 67]. Among the 12 *trans*-pQTLs identified therein, only two co-localized with known eQTLs for the same gene (based on VannoPortal [43], “Methods”). Last, only one gene located at the *trans*-pQTL locus was shown to interact with the associated gene (*SORT1* and *GRN*, respectively; physical or regulatory interactions reported in STRING-db, Methods). Indeed, *GRN* encoded protein, Progranulin, was shown to bind to *SORT1* encoded protein, Sortilin 1, based on co-immunoprecipitation experiments performed in various mice cell lines [68–70] and in green monkey fibroblasts, and on co-expression experiments in human breast cancer cell lines [71, 72] (Additional file 7: Table S6). Individually considered, *cis*-pQTLs explained a mean of 12.6% of the associated protein levels (marginal correlation interquartile range from 4.3 to 14.6%). The highest variance explained (marginal correlation, CAR score, “Methods”) by *cis*-pQTLs were for the rs7041 polymorphism and vitamin D-binding protein (65.6%), rs2856448 for the Tenascin-X protein (61.5%) and rs60642321 for the Complement factor H-related protein 1 (54.4%). Significant *trans*-pQTLs explained a mean of 12.9% of the variability of the associated protein levels (interquartile range from 8.1 to 14.6%), with a maximum contribution of 32.2% in the case of the rs115094736 SNP for the Transferrin receptor protein 1. Considering all *cis*-pQTLs and all *trans*-pQTLs, they explained on average 16.2 and 14.1% of the total variance, respectively. The per-protein global contribution of *cis*-QTLs to plasma-level heterogeneity were lower for immune-related proteins as compared to the rest of the evaluated proteins (13.8% and 19.5% on average, respectively, two-sided Wilcoxon test *p*-value = 0.10).

### Accounting for blood-cell fractions reveals new genetic associations

To evaluate the impact of considering blood-cell fractions in the evaluation of genetic associations with plasma protein levels, we tested whether a linear model accounting for cell fractions better fits protein levels than a simpler model not considering them as covariates. Out of the 112 pQTLs reported in this work, the addition of cell fractions significantly improved the linear model in 42 of the cases (one-way ANOVA, F test *p*-values ≤ 0.05, “Methods”). To characterize the effect of considering or not such covariate, we repeated a genome-wide pQTL assessment as previously described, while excluding blood-cell fractions from the association tests (“Methods”). Under this setting, we found 115 genetic associations with 94 proteins, as compared to the 112 associations with 87 proteins initially identified, with 84 proteins with pQTLs common to both datasets. Thus, three proteins were specific to the analysis accounting for blood-cell fractions as covariates, while 10 proteins were specific to the analysis not considering blood-cell fractions (Additional file 3: Table S2, “Methods”). Moreover, 6 of the 13 proteins showing different pQTL results between the two settings were in turn significantly associated with at least one of the seven circulatory cell fractions tested herein (CEACAM6, Hemopexin, Resistin, TARC, Thrombospondin 1, and TTR; Additional file 3: Table S2, “Methods”). Yet, the nominal *p*-values of genetic associations (*p*-value ≤ 0.05) from one analysis to the other remained significant. In addition, out of the initial 112 significant SNP-protein pairs, only 92 pairs (82%, collectively associated to 76 proteins, and including pairs involving SNPs in high LD, *r*^2^ ≥ 0.8) reached statistical significance in the second setting. Among the protein-SNP pairs not being replicated, 11 proteins (initially associated with 13 SNPs) associated with different SNPs (or with SNPs in lower LD with the previously associated SNPs, *r*^2^ < 0.8), and 7 protein-SNP pairs, involving 7 proteins, were no longer significant. Collectively considered, the *p*-value distribution of the 135 unique SNP-protein pairs (i.e., the 92 pairs common to both analyses, the 20 pairs specific to the first analysis and the 23 pairs specific to the second analysis) corrected for blood-cell fractions was significantly different from the *p*-value distribution obtained for the same protein-SNP pairs while not controlling for blood-cell fractions (two-sided paired Wilcoxon test, *p*-value = 1.97e−06; Additional file 2: Figure S4, Additional file 3: Table S2 and Additional file 7: Table S6). In addition, while no significant differences were observed between the effect size distributions of the two settings (two-sided paired Wilcoxon test, *p*-value = 0.86), controlling for cell fractions lead to a significant shift towards lower effect-size standard deviations (two-sided paired Wilcoxon test, *p*-value < 5.68e−23; Additional file 2: Figure S4). Overall, these results show that cell fractions are an important factor for the study of genetic and non-genetic associations with plasma protein variability across healthy individuals. However, a mediator role cannot be directly inferred from the previous associations.

### Replication of previously reported plasma proteins presenting cis-pQTLs

**Fig. 4:**
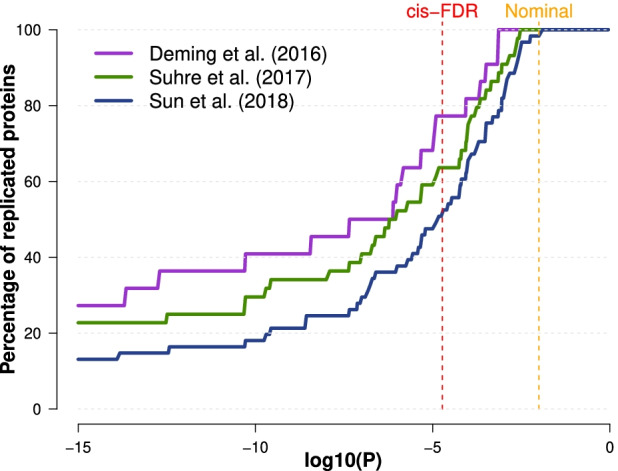
Replication of *cis*-pQTLs. The percentage of replication of previously reported *cis*-regulated proteins between our analysis and three previous studies: Sun et al. [22], Suhre et al. [23], and Deming et al. [24]. For each dataset, the percentage of replication (*y*-axis) as a function of the significance threshold (*x*-axis) was computed as the number of *cis*-regulated proteins reported in this work that were also reported in the corresponding dataset as *cis*-regulated (the “replicated” proteins) divided by the total number of proteins reported as *cis*-regulated in a previous study that were analyzed in our work (the “replicable” proteins). The dashed vertical lines represent the *p*-value significance threshold corresponding to the FDR of *cis*-pQTLs (red) and to the nominal replication threshold (orange)

We then evaluated the extent to which our study replicated previously reported plasma protein associations with proximal genetic polymorphisms (i.e., *cis*-pQTLs) in three recent large-scale studies [22–24, 34] (Additional file 4: Table S3). At a significance threshold based on FDR correction (“Methods”), we replicated 52.2%, 63.6%, and 77.3% of the *n* = 87 proteins reported with significant associations by the three studies, out of the *n* = 131 plasma proteins common with our study, for Sun et al. [22], Suhre et al. [23], and Deming et al. [24] respectively (Fig. 4). From a complementary perspective, 54 out of 78 (66.7%) and 5 out of 11 (45.5%) of the proteins with *cis*- and *trans*-pQTLs in our study, respectively, had been previously reported by at least one of 4 large-scale studies [22–24, 44]. Conversely, we identified 14 novel *cis-* and 5 novel *trans*-pQTLs associated with 15 plasma proteins (Additional file 7: Table S6), absent from reference repositories (i.e., the GWAS Catalog [41] and QTLbase [42], “Methods”) and large-scale plasma pQTL mapping studies [22–24, 44].

### Clinical relevance of plasma proteins and associated genetic factors

**Fig. 5:**
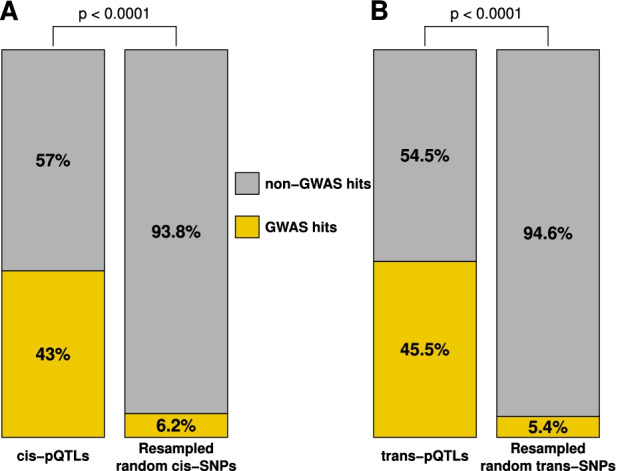
Clinical relevance of pQTLs and associated genes. Enrichment in GWAS hits (orange) of *cis*-pQTLs (**A**—left) and *trans*-pQTLs (**B**—left) in comparison with, respectively, 10,000 randomly sampled set of *cis*-SNPs matched by MAF (bins of 5%) (**A**—right) and 10,000 randomly sampled set of *trans*-SNPs matched by MAF (bins of 5%) (**B**—right). Empirical resampling *p*-values are shown

**Fig. 6:**
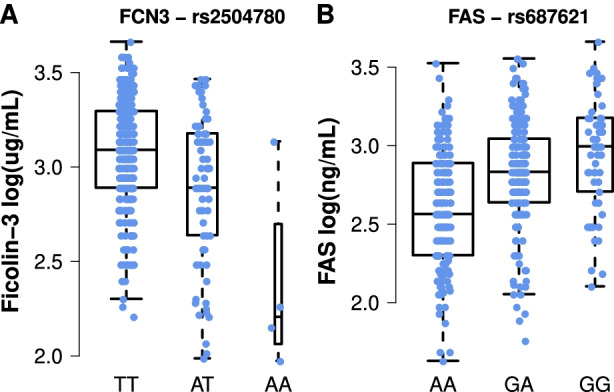
Impact of the novel pQTLs identified on the expression of the 3 target primary immunodeficiency genes. Expression levels of the two homozygous states and the heterozygous state of **A** Ficolin 3 × rs2504780 and **B** FAS × rs687621. Each dot corresponding to the non-transformed plasma levels of an individual of the corresponding genotype

We characterized the potential medical interest of the pQTLs identified and their associated genes. Both *cis-* and *trans-*pQTLs reported in our study were significantly enriched in GWAS-based disease- or trait-associated SNPs, showing ~7 and ~7.8 times more GWAS hits, respectively, than expected (43% and 41.6% observed as compared to an expectation of 6.2% and 5%, odds ratio of 12 and 15.8 respectively, with a resampling *p*-value < 1e−04; “Methods,” Fig. 5, Additional file 7: Table S6). Seventeen of the 87 proteins with pQTLs are FDA-approved biomarkers, including one plasma protein not previously associated with plasma pQTLs, i.e., Cancer Antigen 15-3. In addition, among the 87 genes collectively associated to the 112 pQTLs identified, 29 genes are known monogenic Mendelian disease genes (MMDGs), including six primary immunodeficiency genes (PIDs) (“Methods”). Notably, the plasma protein levels of five out of these 29 MMDGs, including two PID genes, i.e., *FCN3* and *FAS*—had not been previously reported to be associated with these genetic loci (pQTLs) in reference repositories (Fig. 6, “Methods”). The identification of pQTLs associated to such Mendelian disease genes may contribute to the genetic characterization of the observed incomplete penetrance or severity heterogeneity across patients suffering from primary immune deficiencies.

## Discussion

In this work, we characterized non-genetic and genetic factors explaining the natural heterogeneity of 229 plasma protein levels observed in healthy individuals. We replicated previous findings [22–24, 44, 51–53] describing that age and sex have a global impact on plasma proteins, while anthropometric variables, blood lipids, and metabolic markers are also relevant factors for specific proteins. In agreement with previous observations from this cohort [29, 31], environmental factors had a relatively low influence on protein levels in this study of well-defined healthy donors. In addition, we characterized the contribution of seven major blood-cell fractions to inter-individual heterogeneity and found that their contribution to plasma protein variability was higher than age and sex. Moreover, our results suggest that blood-cell fractions may act as important confounders of genetic associations with specific plasma protein levels. In addition to non-genetic factors, we identified 100 and 12 pQTLs acting in *cis* and *trans*, respectively, associated with 87 plasma proteins. However, the inclusion of cellular covariates in the assessment of genetic associations led to the identification of three novel proteins with pQTLs, while abrogated the signal for 10 proteins which would have otherwise led to positive hits. This could potentially be explained by the fact that all 13 proteins are expressed by specific circulating immune cell populations [73–82]. However, the interactions between these proteins, blood-cell populations, and genetic variants are less obvious to interpret, as both direct and indirect effects or co-occurring mechanisms could be involved. Some limitations of our study include the relatively modest sample size, and the focus on two age groups to identify age associations. This approach could be improved by including donors from more extreme ages of life such as neonates, pediatrics, and greater than 80 years when age may be expected to have stronger effects on physiological processes. An additional limitation is the analysis of a single homogeneous population that can be addressed through inclusion of populations from other ethnicities and backgrounds in future studies.

Although our study replicated a large number of previously reported genetic associations with plasma proteins [22–24, 44], it also identified 19 novel pQTLs associated with 15 proteins. This may stem from the well-defined healthy nature of our study population, which may reduce potential confounding lifestyle or medical factors, or from the use of quantitative values as provided by Luminex assays as compared to alternative assessment methods [83]. Interestingly, of the newly identified associations, six include proteins encoded by MMDGs, 2 of which are known to cause primary immunodeficiencies, i.e., Ficolin-3 and FAS (Fig. 6). Primary immunodeficiencies are caused by rare variants leading either to loss- or gain-of-function consequences in the affected genes [84, 85]. However, such mutations are often not fully penetrant and the associated symptoms are heterogeneous between and within families. Among possible explanations for this heterogeneity, low to mild effect common variants, such as the pQTLs identified in this work, might act as modifier of the corresponding diseases, by increasing or decreasing the expression of the corresponding proteins, and consequently mitigating or aggravating the consequences of causal variants.

Thus, the common variant associated with Ficolin-3 plasma levels identified in this work, rs2504780 (AF = 10.7%, 1:27710876, T>A), is located 9.5kb upstream of *FCN3* and associated with a diminution of Ficolin-3 levels (effect size = − 3.79 μg/mL per alternative allele, Fig. 6A) of an order of magnitude comparable with heterozygous *FCN3* loss-of-function variants (effect size = − 13.4 μg/mL per loss-of-function allele) [86] causing immunodeficiency 41 with lymphoproliferation and autoimmunity (OMIM 606367) [87–92]. This variant could be a risk factor for Ficolin-3 deficiency and might play a role in the observed etiology of both complete Ficolin-3 deficiency or Ficolin-3 haploinsufficiency in the response to infection and autoimmunity. Future analysis of auto-antibodies in our cohort may allow us to directly test this hypothesis. Another example is the common variant associated in *trans* with FAS plasma levels, rs687621 (9:136137065, A>G, AF = 36%) which increases the expression of FAS (effect size = +2.79 ng/mL per alternative allele; Fig. 6B). This variant could contribute to a protective role against the haploinsufficient forms of autoimmune lymphoproliferative syndrome (ALPS, OMIM 601859) [93–98] by increasing the expression of FAS in heterozygous loss-of-function variant carriers. However, the rs687621 polymorphism is located at the *ABO* locus, which is known to associate with the expression of many plasma proteins [22, 24, 99, 100]. Such an association hotspot could be explained by the glycosyltransferase activity of ABO proteins [101], which by transferring glycosyl residuals on target proteins may potentially alter its binding affinity of the associated antibody in immunoassays [102], thus constituting a technical artifact. In light of these potential caveats, the biological relevance of the FAS-associated *trans*-pQTL identified should be taken with caution, prior to replication in other cohorts with complementary assays. The common genetic associations identified here for plasma protein levels of PID genes could be further characterized through genetic fine-mapping and functional characterization.

Plasma protein levels can be considered as end-of-chain signal integrators, and their levels are influenced by several molecular mechanisms (e.g., mRNA transcription, Kozak sequence affinity and other translation initiation mechanisms, codon usage, translation rate, post-transcriptional modifications [103–107]). A combination of targeted genome and transcriptome sequencing, ribosome occupancy assay, and intracellular protein assays in the cell type or tissue of interest would allow the identification of the causal variants and the molecular mechanisms mediating the observed associations. Finally, the phenotypic consequences of pQTLs associated to plasma levels coded by PID genes should be further characterized both in healthy and PID patients, where protective or modifier roles could be further established.

## Conclusions

Our study showed that, together with age and sex, circulatory blood-cell fractions are a major factor explaining the inter-individual heterogeneity of plasma protein levels in the general population. In addition, 100 cis- and 12 trans-pQTLs were identified, which explained the largest fraction of the variability of plasma protein levels as compared to non-genetic factors. Such pQTLs were significantly enriched in GWAS-based disease- or trait-associated SNPs. Furthermore, among 19 previously unreported genetic associations, 6 involved 5 known monogenic Mendelian disease genes, including 2 primary immunodeficiency genes. The potential use of the identified genetic and non-genetic factors associated to plasma protein levels as biomarkers to inform health and disease status would require further investigation.

## Supplementary Information

**Additional file 1: Table S1.** List of the 297 plasma proteins assayed.

**Additional file 2: Figure S1.** Principal component analysis of age and sex effects. **Figure S2.** Manhattan plots and allelic expression of levels of cis-pQTLs. **Figure S3.** Manhattan plots and allelic expression of levels of trans-pQTLs. **Figure S4.** Impact of blood-cell fractions on associated protein-SNP pairs statistics.

**Additional file 3: Table S2.** Proteins with analysis-specific significant genetic association.

**Additional file 4: Table S3.** Summary of previous plasma pQTL study design.

**Additional file 5: Table S4.** Description of the association between plasma protein levels, age, sex and blood-cell fractions.

**Additional file 6: Table S5.** Description of the significant protein-environmental variable pairs and variable contribution the variability of the protein expression levels.

**Additional file 7: Table S6.** Annotations of cis and trans pQTLs.

## Data Availability

The SNP array data that support the findings of this study have been deposited in the European Genome-Phenome Archive (EGA) with the accession code EGAS00001002460 [27, 29–32] (https://ega-archive.org/studies/EGAS00001002460). Access to individuals’ genotype, biological readouts (such as plasma protein levels), and phenotype data is provided for research use only after review and approval by the Milieu Intérieur data access committee, in line with patient privacy and confidentiality agreements. Requests can be sent to milieuinterieurdac@pasteur.fr. The 110 summary statistics files corresponding to the 87 proteins with identified pQTLs, containing positional and statistical information for all 5,201,092 evaluated SNPs, are openly available at the GWAS catalog [41] (https://www.ebi.ac.uk/gwas/), through single variant or gene query or through bulk download, with accession numbers ranging from GCST90085705 to GCST90085814. All other data are available in this published article and its supplementary information files.
