# Supplementary material for: Integrative genetic and immune cell analysis of plasma proteins in healthy donors identifies novel associations involving primary immune deficiency genes
Source: Genome Med. 2022 Mar 9;14:28. doi: 10.1186/s13073-022-01032-y (PMC8905727; doi:10.1186/s13073-022-01032-y)
Supplement: Supplementary file 2 — Additional file 2: Figure S1. Principal component analysis of age and sex effects. Figure S2. Manhattan plots and allelic expression of levels of cis-pQTLs. Figure S3. Manhattan plots and allelic expression of levels of trans-pQTLs. Figure S4. Impact of blood-cell fractions on associated protein-SNP pairs statistics. [file 13073_2022_1032_MOESM2_ESM.pdf]

Supplementary Figures

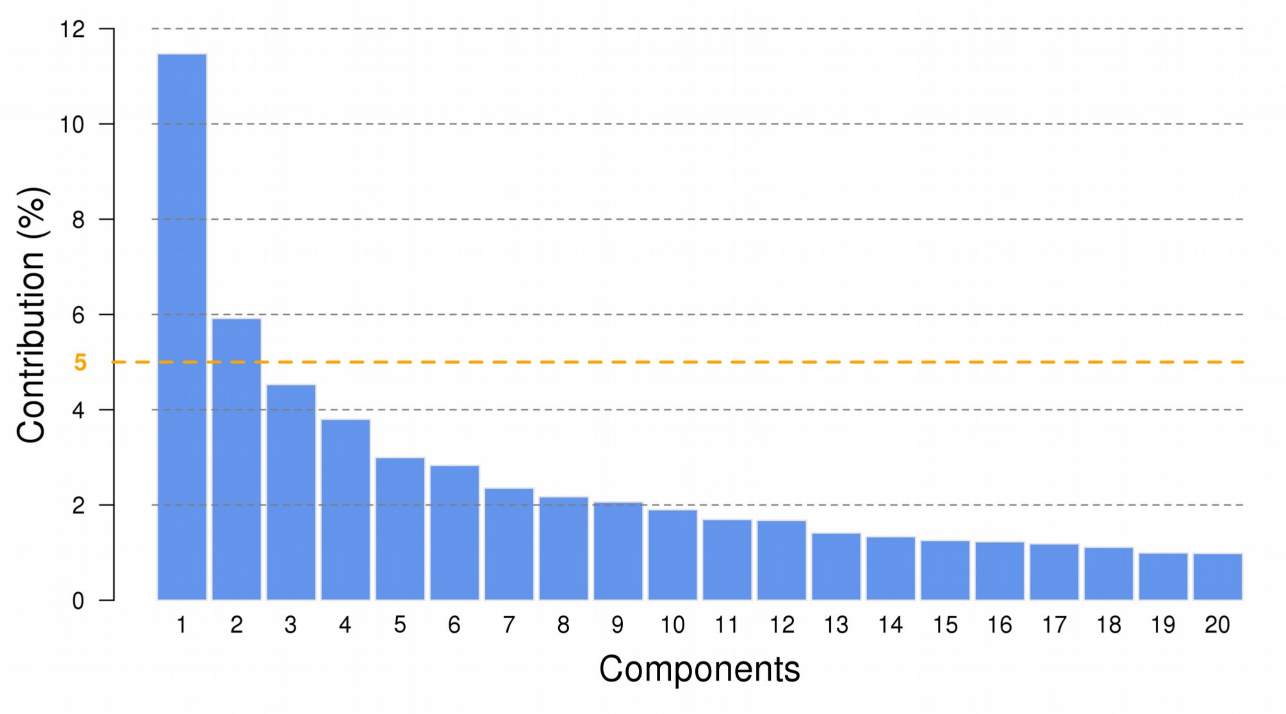

Figure S1: Principal component analysis of plasma proteins

Relative contribution of age, sex and cell fractions on plasma protein levels. The dashed orange line corresponds to 5% of contribution to the total variability.

Figure S2: Manhattan plots and allelic expression of levels of cis-pQTLs

Manhattan plots showing the distribution of genotyped and imputed SNPs in the vicinity of genes coding for proteins with cis-pQTLs identified in our study. The position of the transcript is represented by a box at the bottom of each plot, with the corresponding chromosomal coordinates. The y axis represent the -log10 p-value of associations, and each dot corresponds to a SNP. The sentinel SNP is colored in pink and is identified by its dbsnp id. The other SNPs are colored based on their linkage disequilibrium R^2 with the sentinel SNP, and the associated color scale is shown on the right of the plot. The horizontal blue line represent the p value threshold corresponding to the cis-FDR level. Additionally, the expression levels of the two homozygous states and the heterozygous state of the corresponding cis-pQTLs are represented on the right, each dot corresponding to the log transformed plasma levels of an individual. If a second cis-pQTL was found during the conditional analysis, it is represented as a second pair of Manhattan and allelic expression plots. The Manhattan plots representing conditional cis-pQTLs are showing the conditional sentinel SNP in pink, while the previously identified sentinel SNP is represented in green. Both are labeled with their dbsnp id.

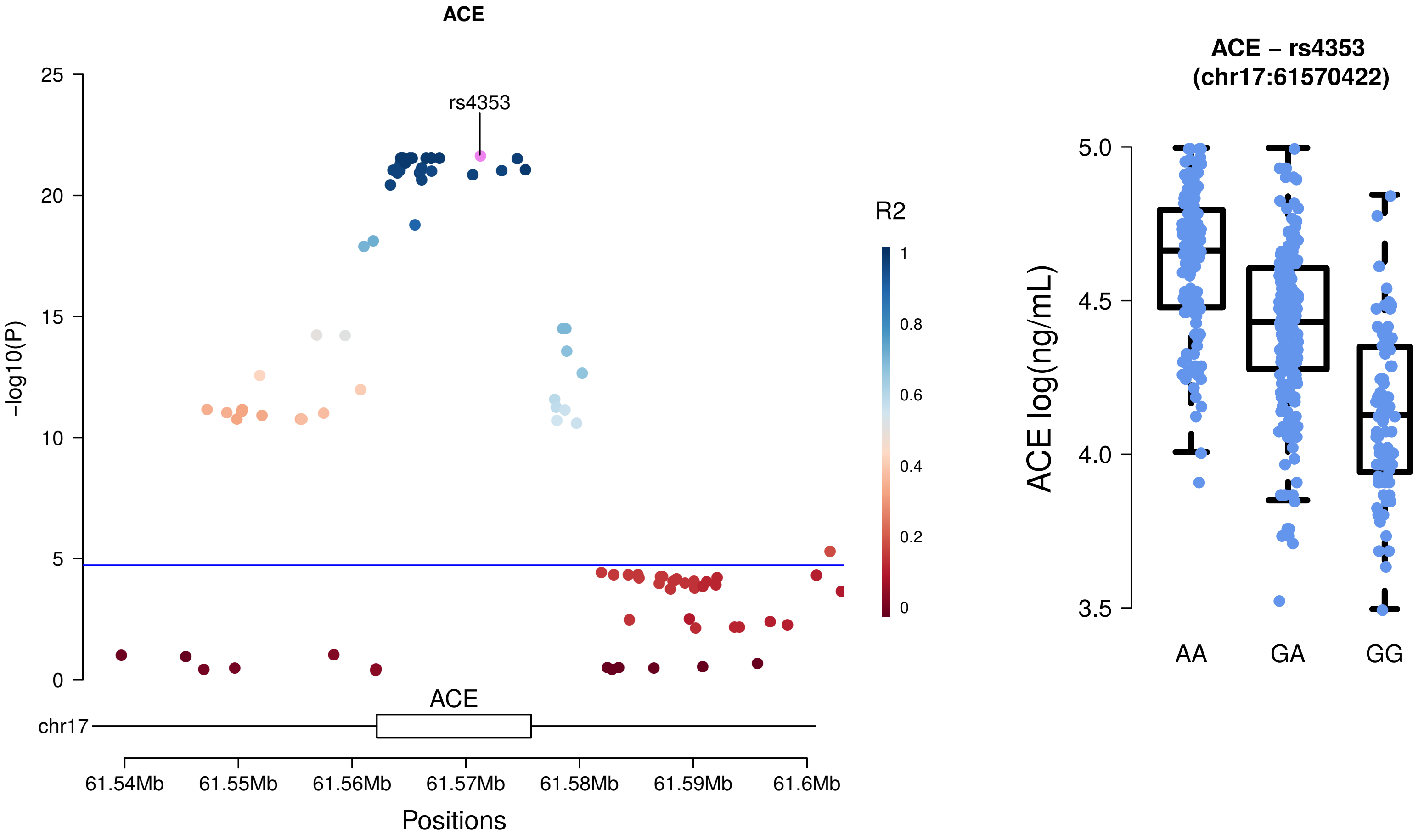

ACE
rs4353
25
20
15
10
5
0
-log10(P)
R2
1
0.8
0.6
0.4
0.2
0
ACE
chr17
61.54Mb
61.55Mb
61.56Mb
61.57Mb
61.58Mb
61.59Mb
61.6Mb
Positions
ACE – rs4353
(chr17:61570422)
5.0
4.5
4.0
3.5
ACE log(ng/mL)
AA
GA
GG

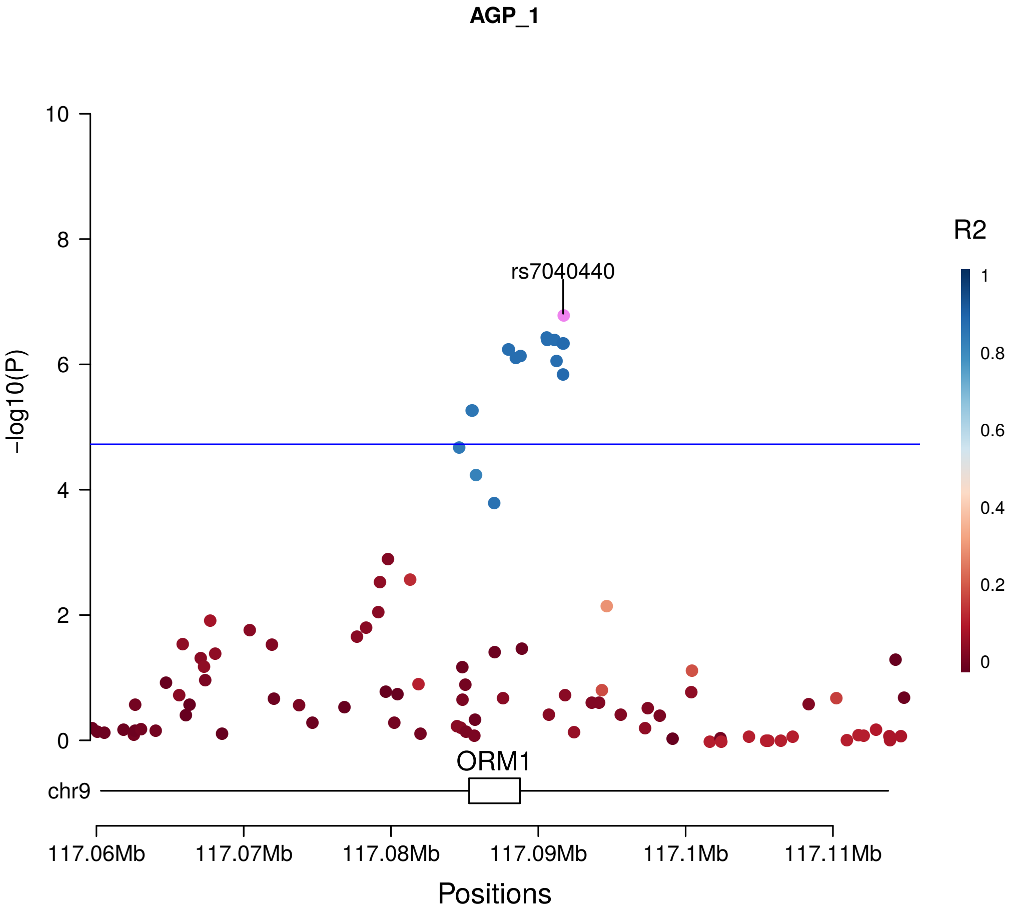
AGP_1
rs7040440
R2
1
0.8
0.6
0.4
0.2
0
10
8
6
4
2
0
-log10(P)
ORM1
chr9
117.06Mb
117.07Mb
117.08Mb
117.09Mb
117.1Mb
117.11Mb
Positions

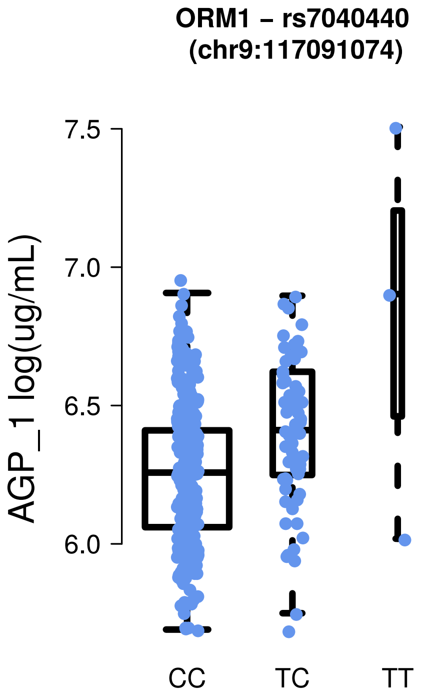
ORM1 – rs7040440
(chr9:117091074)
7.5
7.0
6.5
6.0
AGP_1 log(ug/mL)
CC
TC
TT

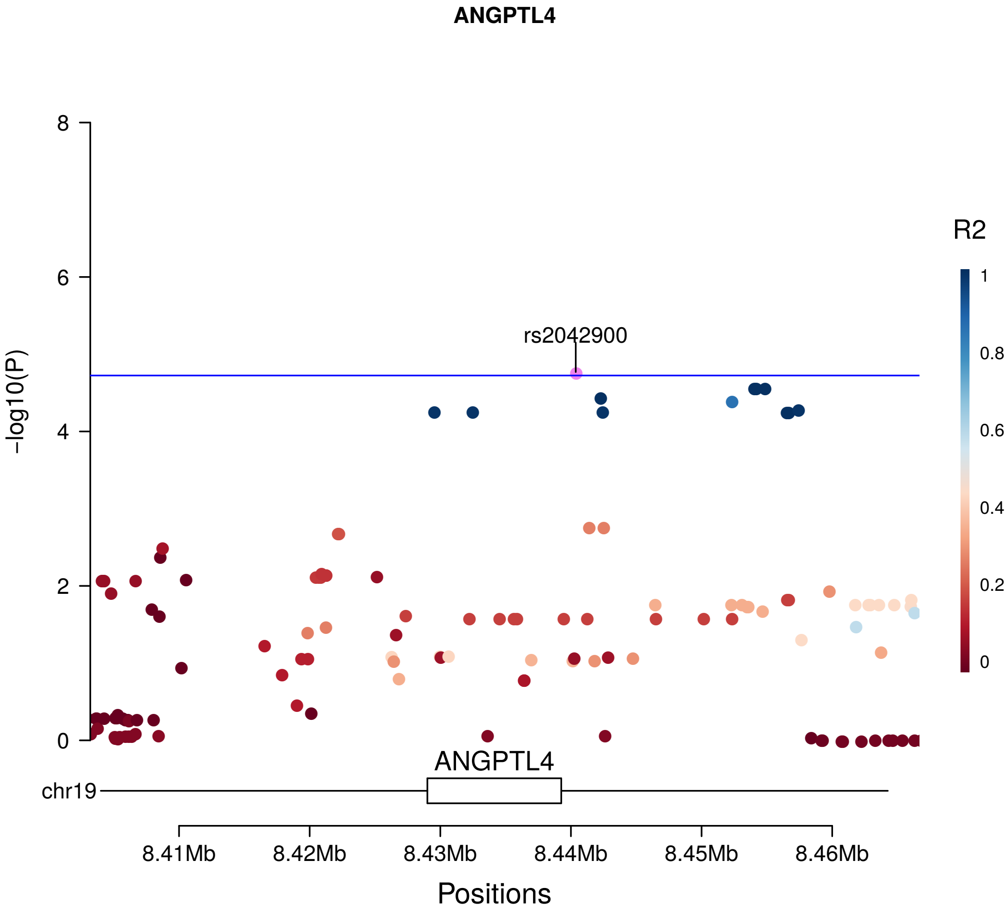
ANGPTL4
R2
1
0.8
0.6
0.4
0.2
0
rs2042900
-log10(P)
8
6
4
2
0
ANGPTL4
chr19
8.41Mb
8.42Mb
8.43Mb
8.44Mb
8.45Mb
8.46Mb
Positions

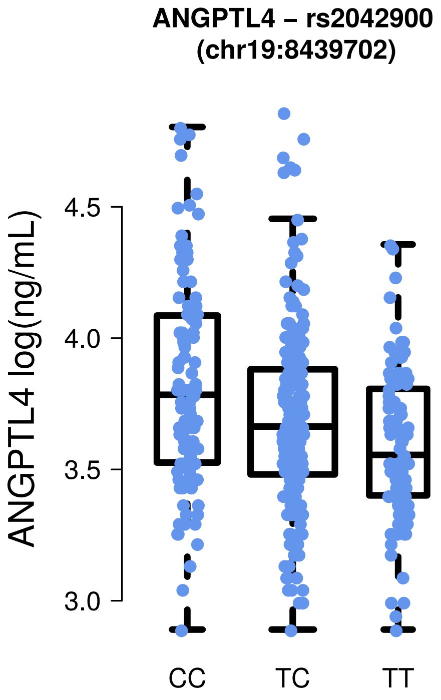
ANGPTL4 – rs2042900
(chr19:8439702)
ANGPTL4 log(ng/mL)
4.5
4.0
3.5
3.0
CC
TC
TT

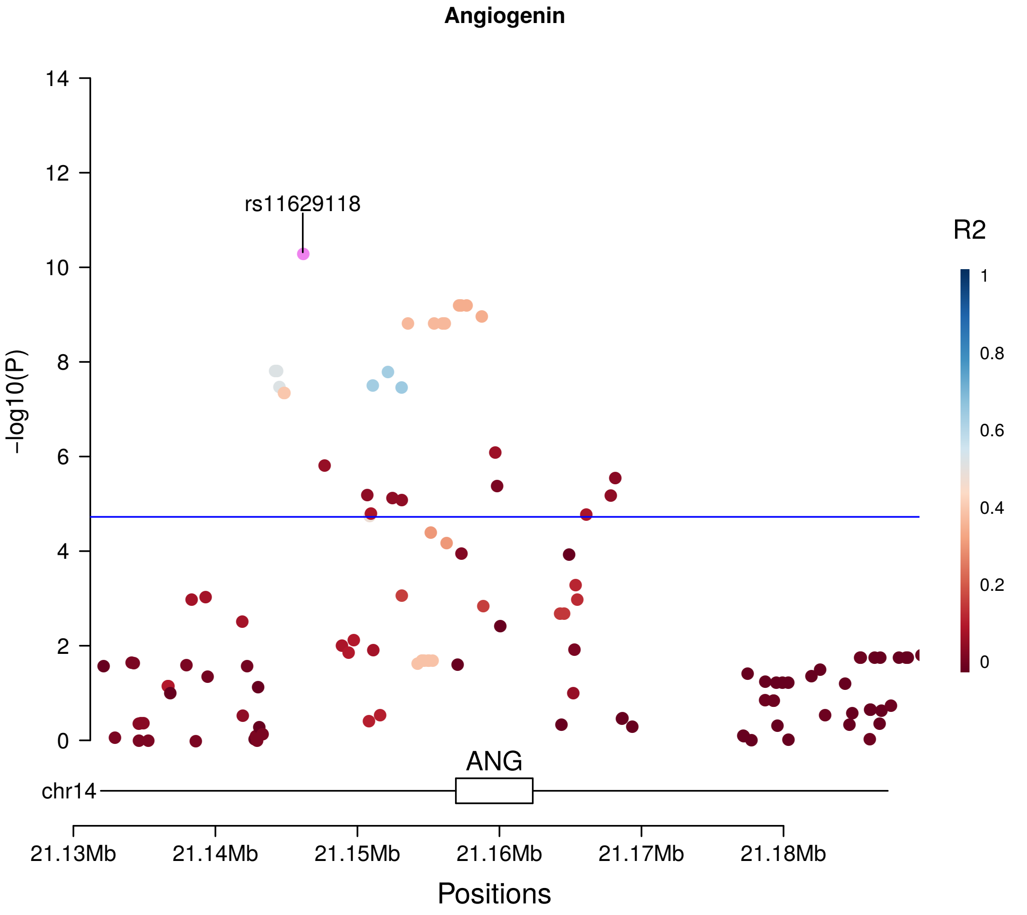
Angiogenin
rs11629118
R2
ANG
chr14
21.13Mb
21.14Mb
21.15Mb
21.16Mb
21.17Mb
21.18Mb
Positions
-log10(P)

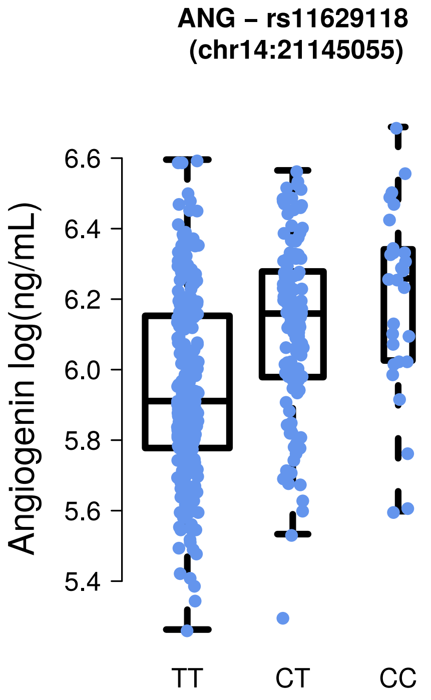
ANG – rs11629118
(chr14:21145055)
Angiogenin log(ng/mL)
TT
CT
CC

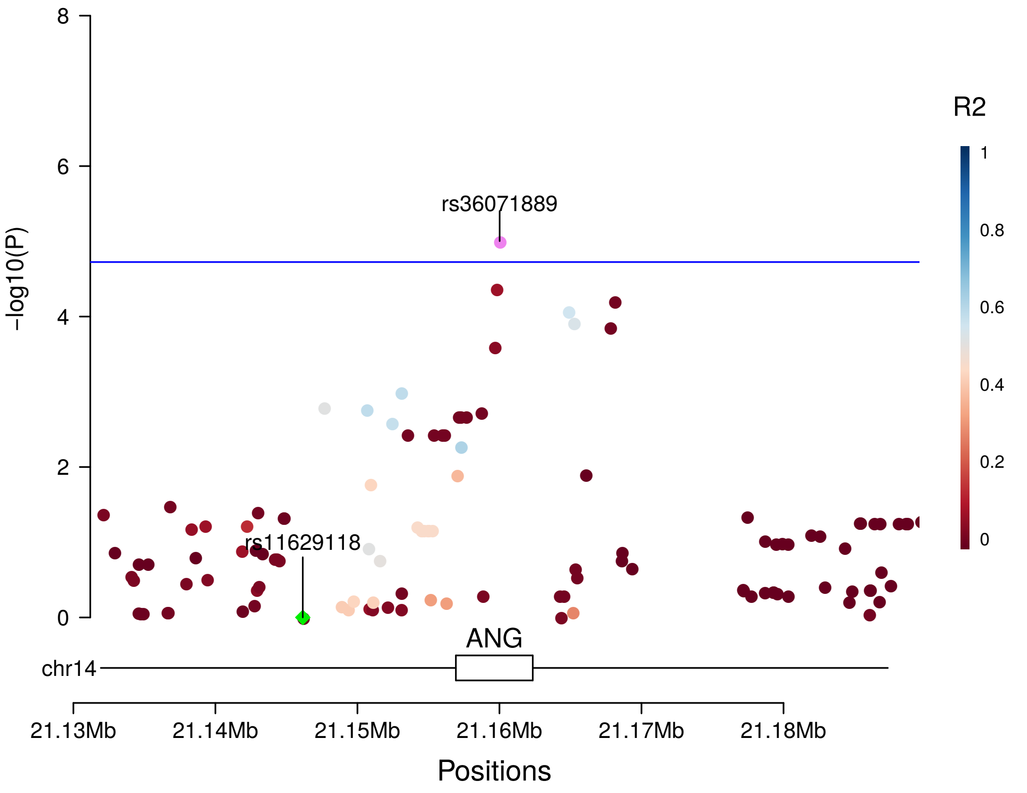
rs36071889
rs11629118
R2
ANG
chr14
21.13Mb
21.14Mb
21.15Mb
21.16Mb
21.17Mb
21.18Mb
Positions
-log10(P)

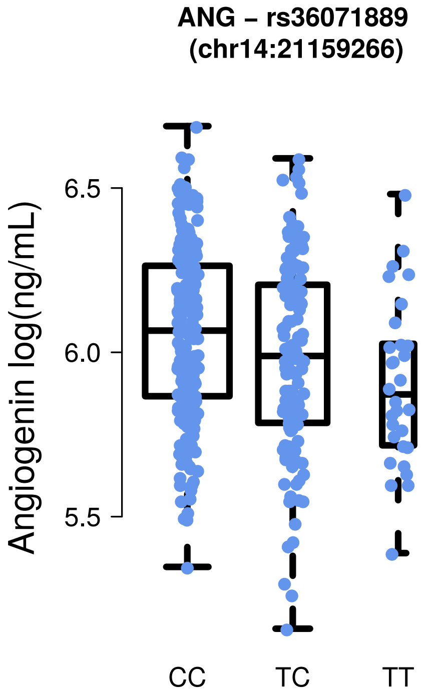
ANG – rs36071889
(chr14:21159266)
Angiogenin log(ng/mL)
CC
TC
TT

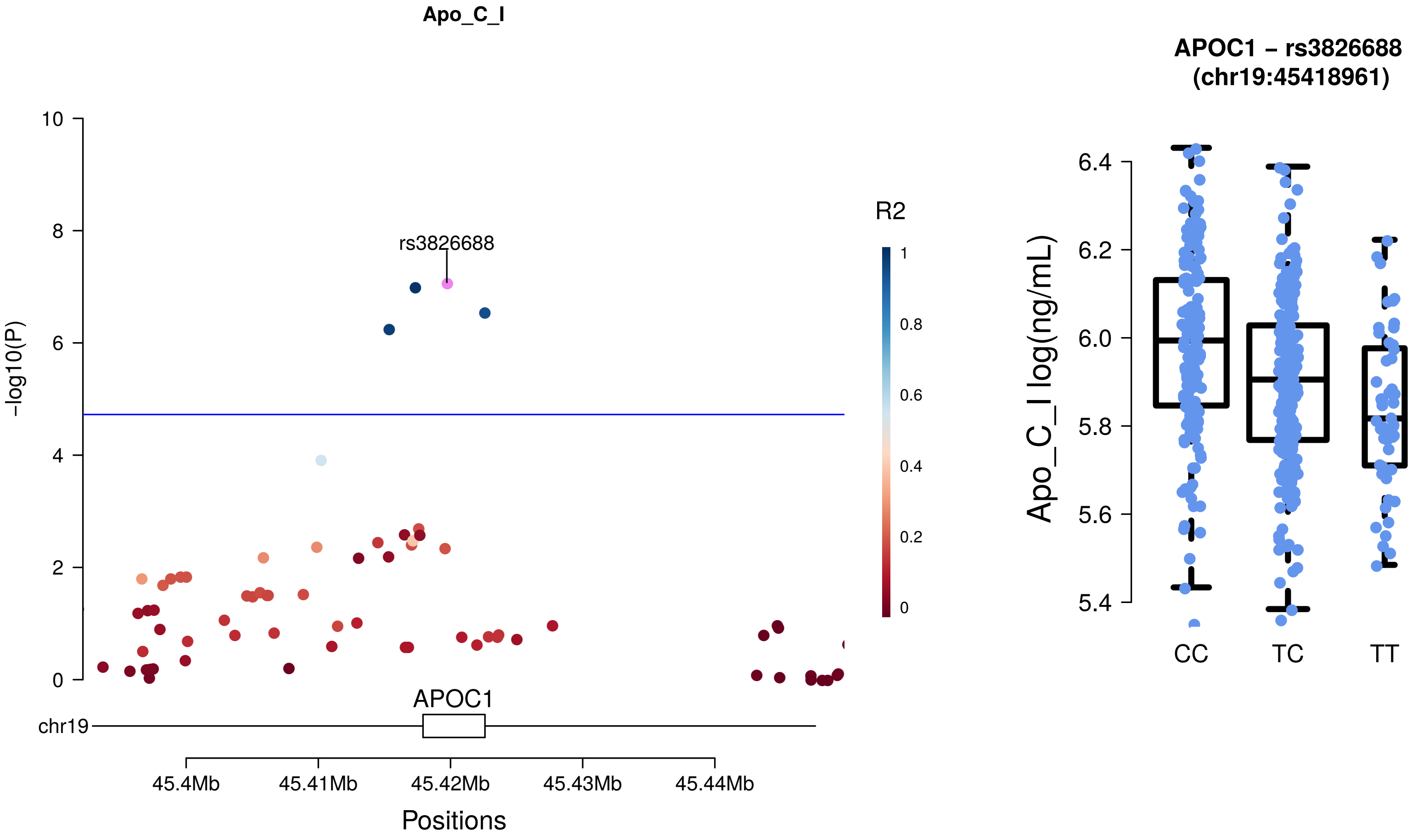
Apo_C_I
-log10(P)
10
8
6
4
2
0
rs3826688
R2
1
0.8
0.6
0.4
0.2
0
APOC1
chr19
45.4Mb
45.41Mb
45.42Mb
45.43Mb
45.44Mb
Positions
APOC1 – rs3826688
(chr19:45418961)
Apo_C_I log(ng/mL)
6.4
6.2
6.0
5.8
5.6
5.4
CC
TC
TT

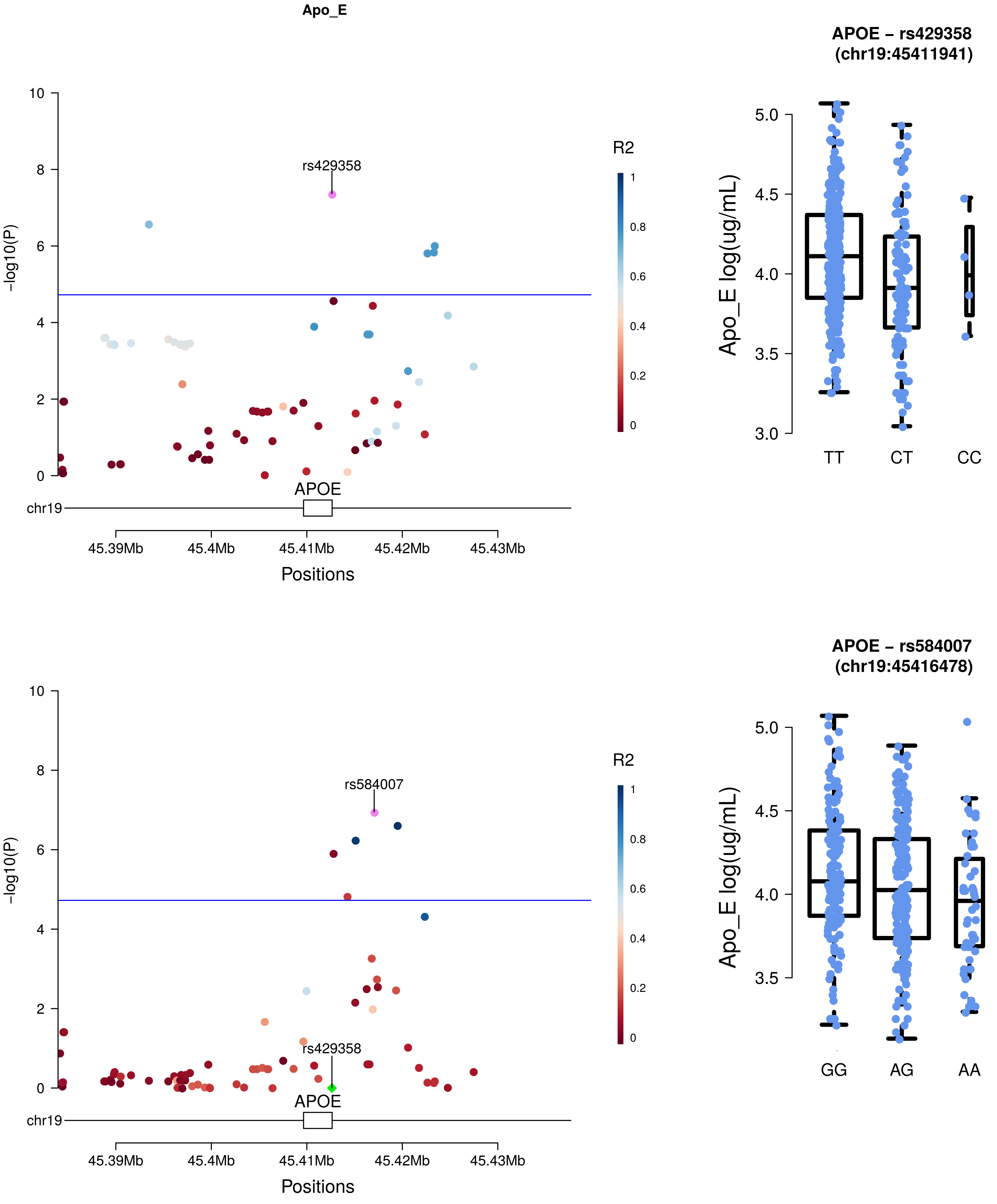
Apo_E
rs429358
R2
1
0.8
0.6
0.4
0.2
0
-log10(P)
10
8
6
4
2
0
APOE
chr19
45.39Mb
45.4Mb
45.41Mb
45.42Mb
45.43Mb
Positions
APOE – rs429358
(chr19:45411941)
Apo_E log(ug/mL)
5.0
4.5
4.0
3.5
3.0
TT
CT
CC
rs584007
rs429358
APOE – rs584007
(chr19:45416478)
GG
AG
AA

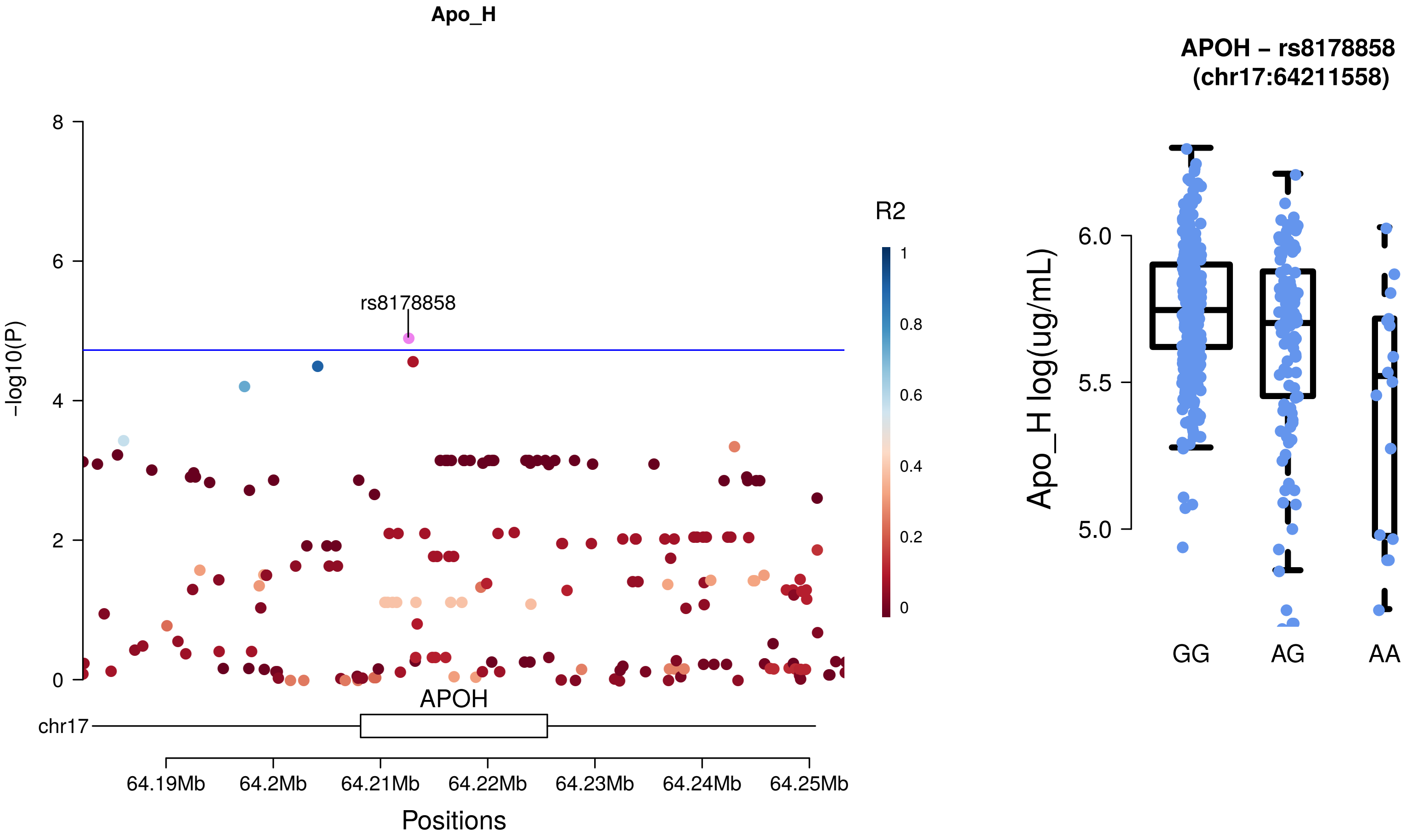

Apo_H
rs8178858
-log10(P)
R2
1
0.8
0.6
0.4
0.2
0
APOH
chr17
64.19Mb
64.2Mb
64.21Mb
64.22Mb
64.23Mb
64.24Mb
64.25Mb
Positions
APOH – rs8178858
(chr17:64211558)
Apo_H log(ug/mL)
6.0
5.5
5.0
GG
AG
AA

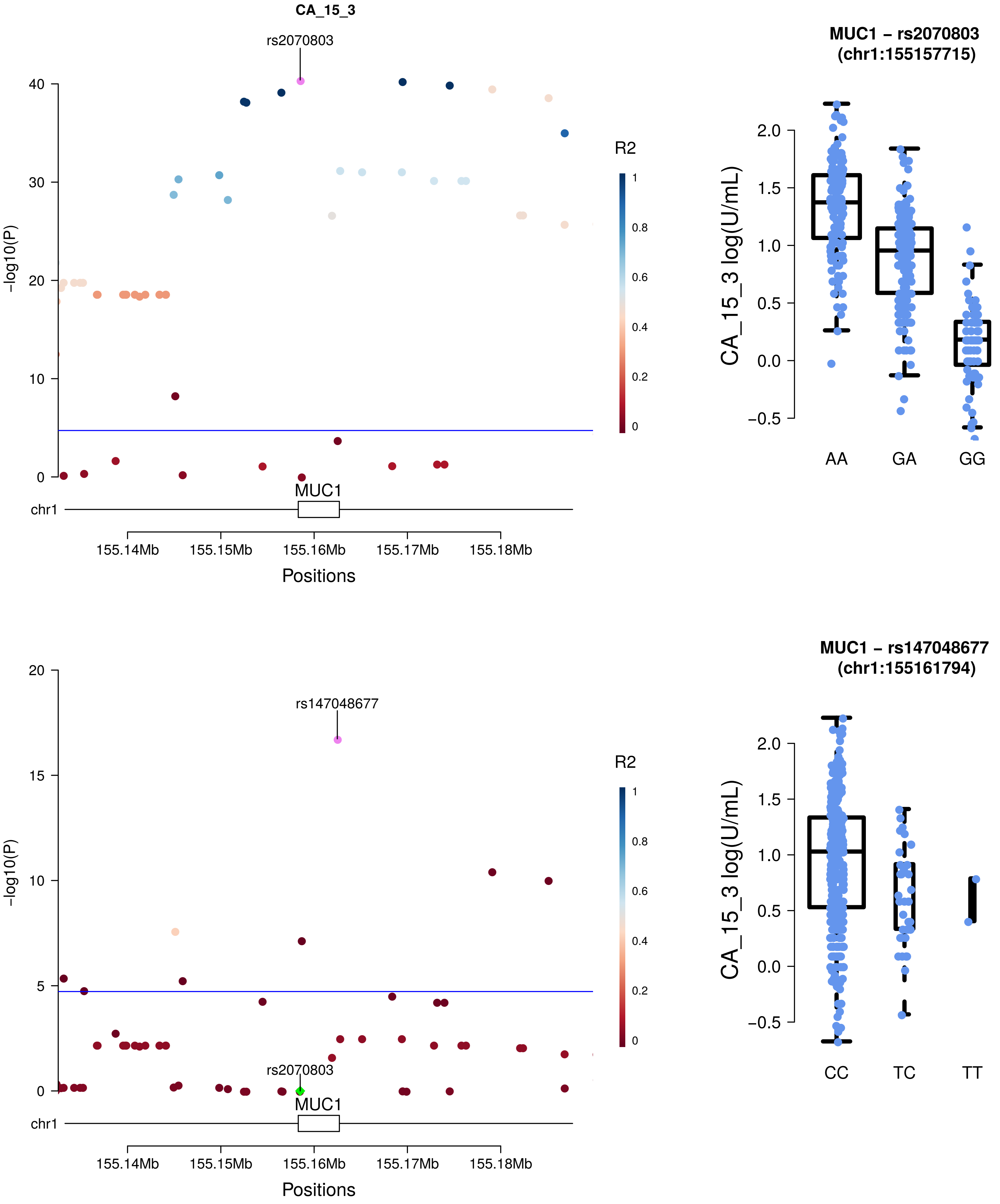
CA_15_3
rs2070803
-log10(P)
40
30
20
10
0
R2
1
0.8
0.6
0.4
0.2
0
MUC1
chr1
155.14Mb
155.15Mb
155.16Mb
155.17Mb
155.18Mb
Positions
MUC1 – rs2070803
(chr1:155157715)
CA_15_3 log(U/mL)
2.0
1.5
1.0
0.5
0.0
−0.5
AA
GA
GG
rs147048677
-log10(P)
20
15
10
5
0
R2
1
0.8
0.6
0.4
0.2
0
rs2070803
MUC1
chr1
155.14Mb
155.15Mb
155.16Mb
155.17Mb
155.18Mb
Positions
MUC1 – rs147048677
(chr1:155161794)
CA_15_3 log(U/mL)
2.0
1.5
1.0
0.5
0.0
−0.5
CC
TC
TT

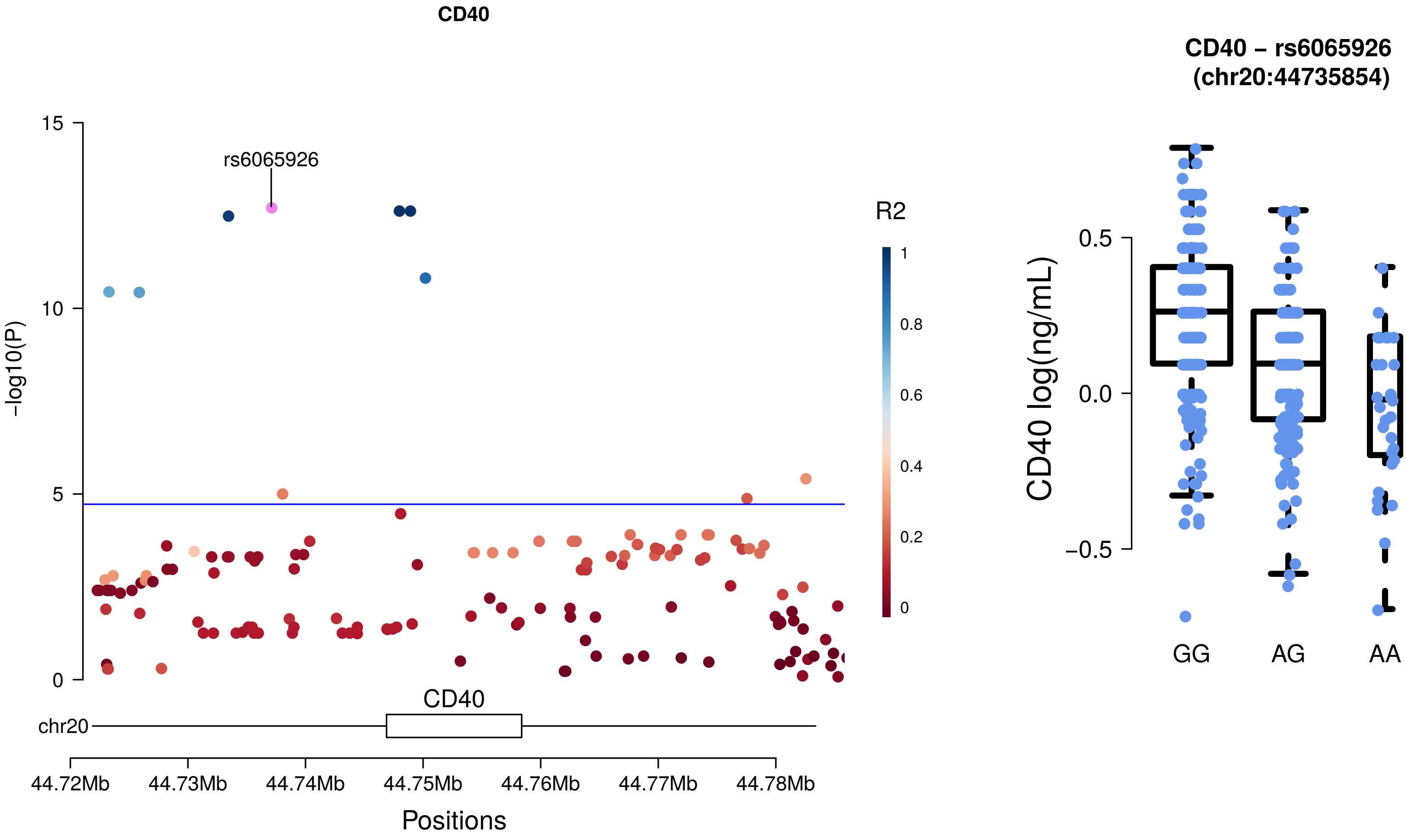
CD40
rs6065926
15
10
5
0
-log10(P)
R2
1
0.8
0.6
0.4
0.2
0
CD40
chr20
44.72Mb
44.73Mb
44.74Mb
44.75Mb
44.76Mb
44.77Mb
44.78Mb
Positions
CD40 – rs6065926
(chr20:44735854)
CD40 log(ng/mL)
0.5
0.0
−0.5
GG
AG
AA

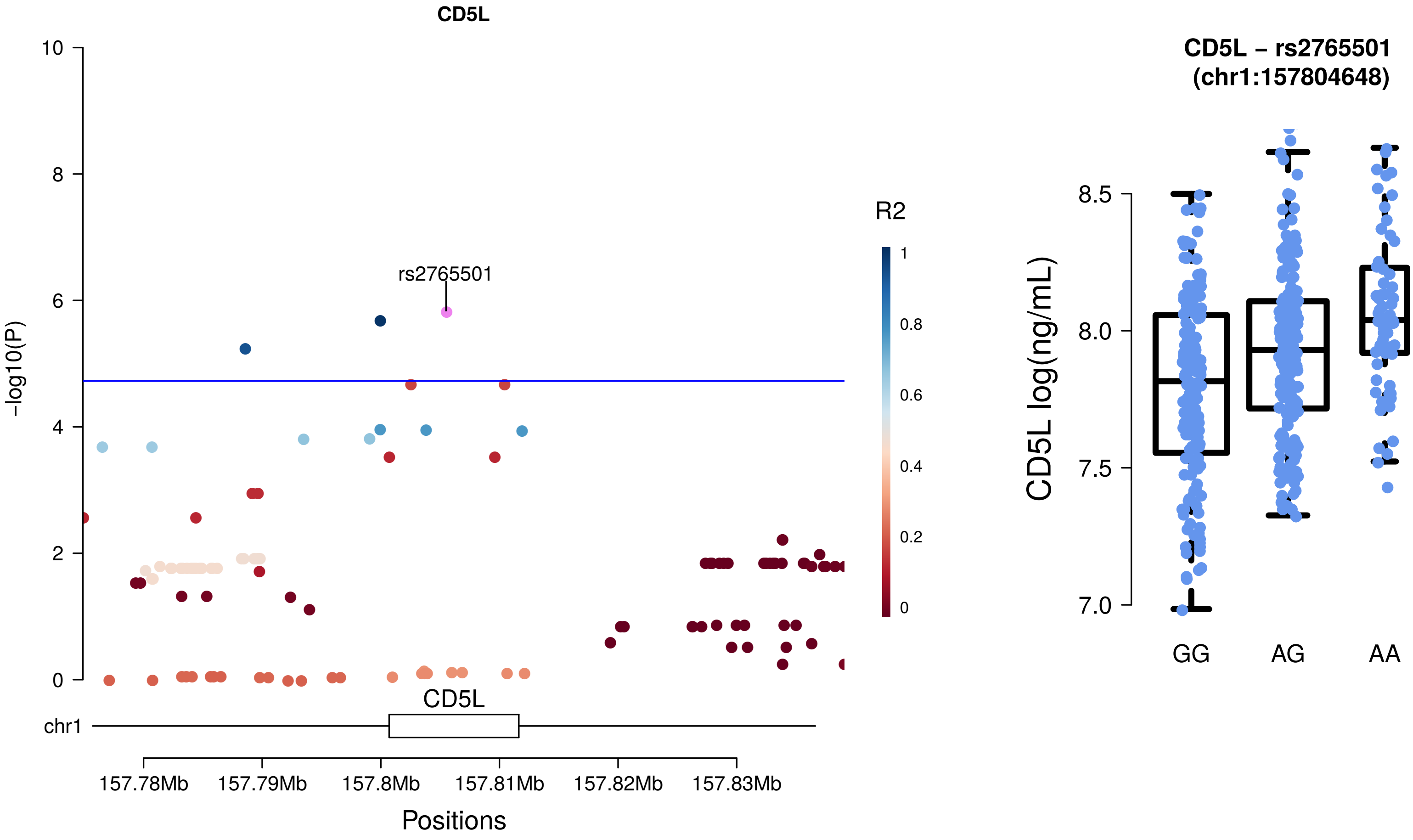

CD5L
10
8
6
4
2
0
-log10(P)
rs2765501
R2
1
0.8
0.6
0.4
0.2
0
CD5L
chr1
157.78Mb
157.79Mb
157.8Mb
157.81Mb
157.82Mb
157.83Mb
Positions
CD5L – rs2765501
(chr1:157804648)
8.5
8.0
7.5
7.0
CD5L log(ng/mL)
GG
AG
AA

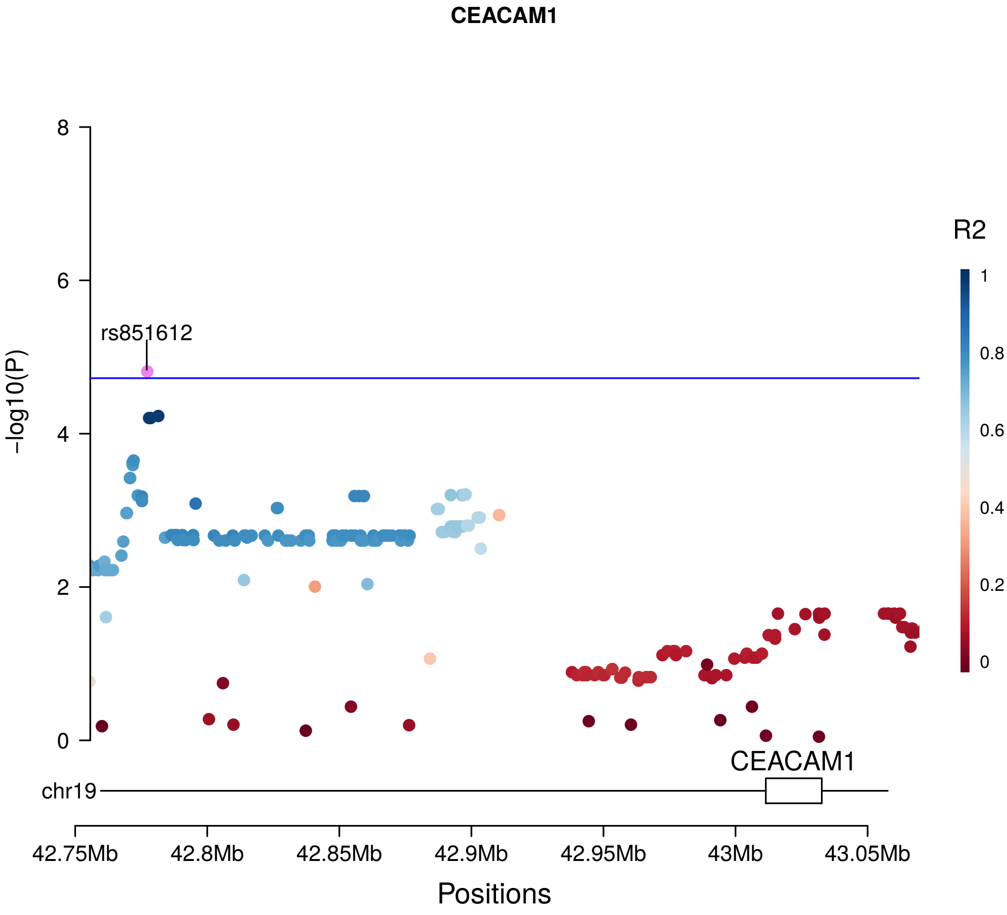
CEACAM1
8
6
4
2
0
-log10(P)
rs851612
R2
1
0.8
0.6
0.4
0.2
0
CEACAM1
chr19
42.75Mb
42.8Mb
42.85Mb
42.9Mb
42.95Mb
43Mb
43.05Mb
Positions

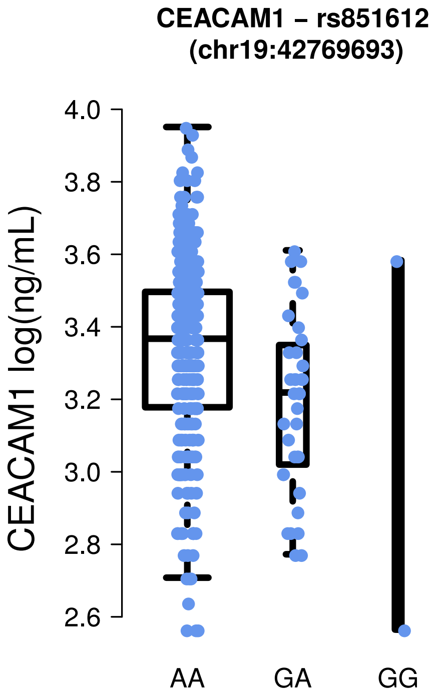
CEACAM1 – rs851612
(chr19:42769693)
CEACAM1 log(ng/mL)
4.0
3.8
3.6
3.4
3.2
3.0
2.8
2.6
AA
GA
GG

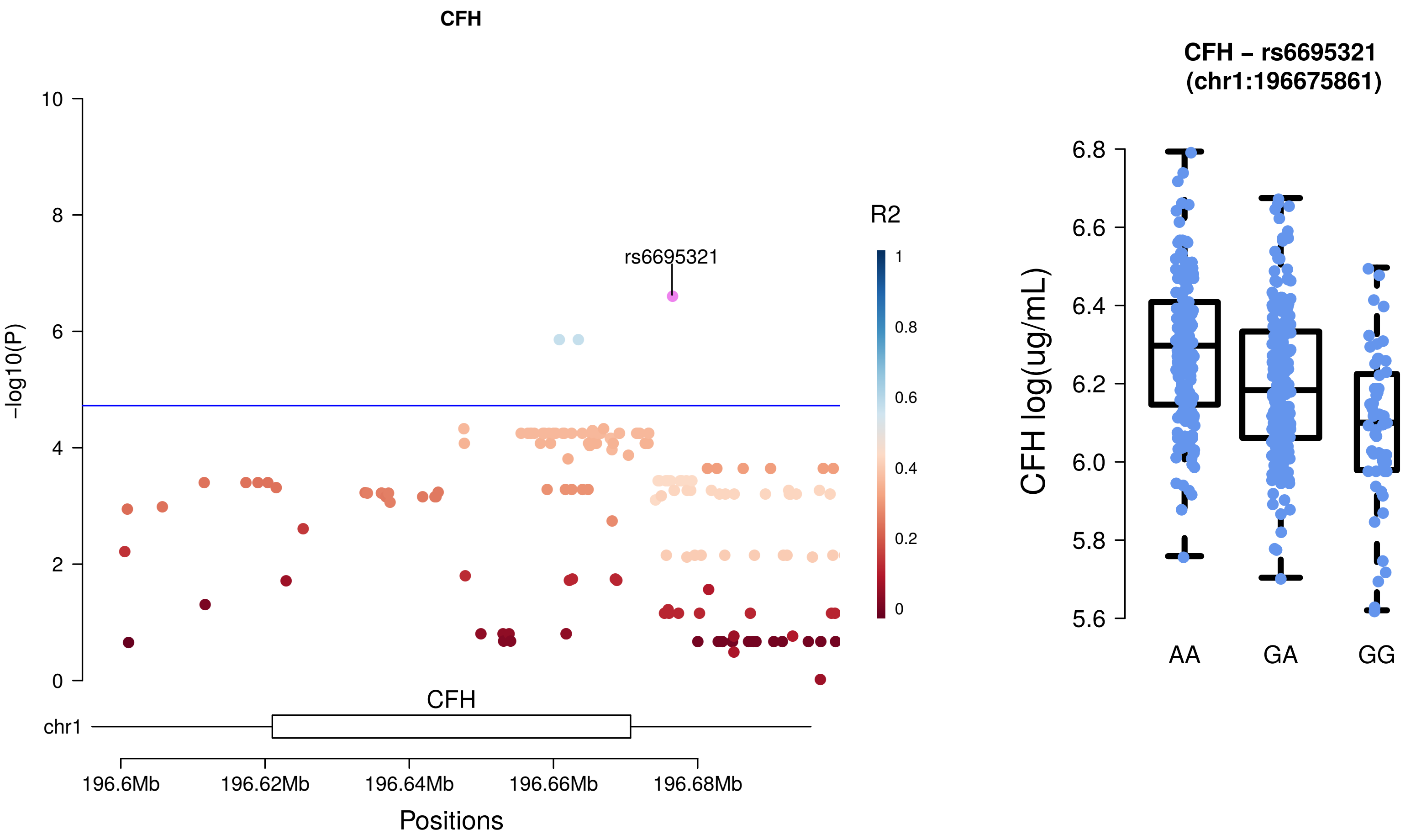

CFH
rs6695321
R2
1
0.8
0.6
0.4
0.2
0
10
8
6
4
2
0
-log10(P)
CFH
chr1
196.6Mb
196.62Mb
196.64Mb
196.66Mb
196.68Mb
Positions
CFH – rs6695321
(chr1:196675861)
6.8
6.6
6.4
6.2
6.0
5.8
5.6
CFH log(ug/mL)
AA
GA
GG

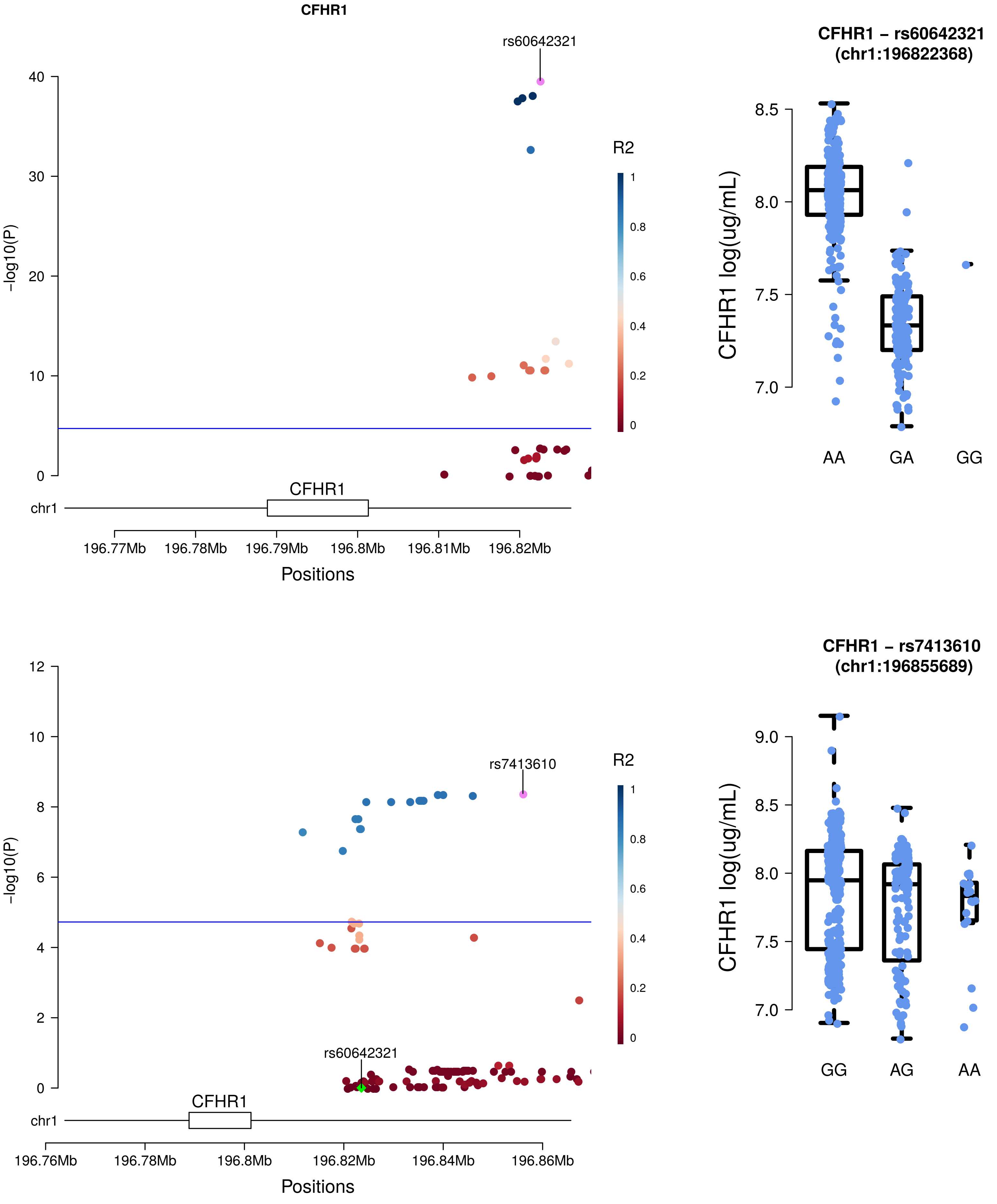
CFHR1
rs60642321
-log10(P)
R2
CFHR1
chr1
196.77Mb
196.78Mb
196.79Mb
196.8Mb
196.81Mb
196.82Mb
Positions
CFHR1 – rs60642321
(chr1:196822368)
CFHR1 log(ug/mL)
AA
GA
GG
rs7413610
rs60642321
196.76Mb
196.78Mb
196.8Mb
196.82Mb
196.84Mb
196.86Mb
CFHR1 – rs7413610
(chr1:196855689)
GG
AG
AA

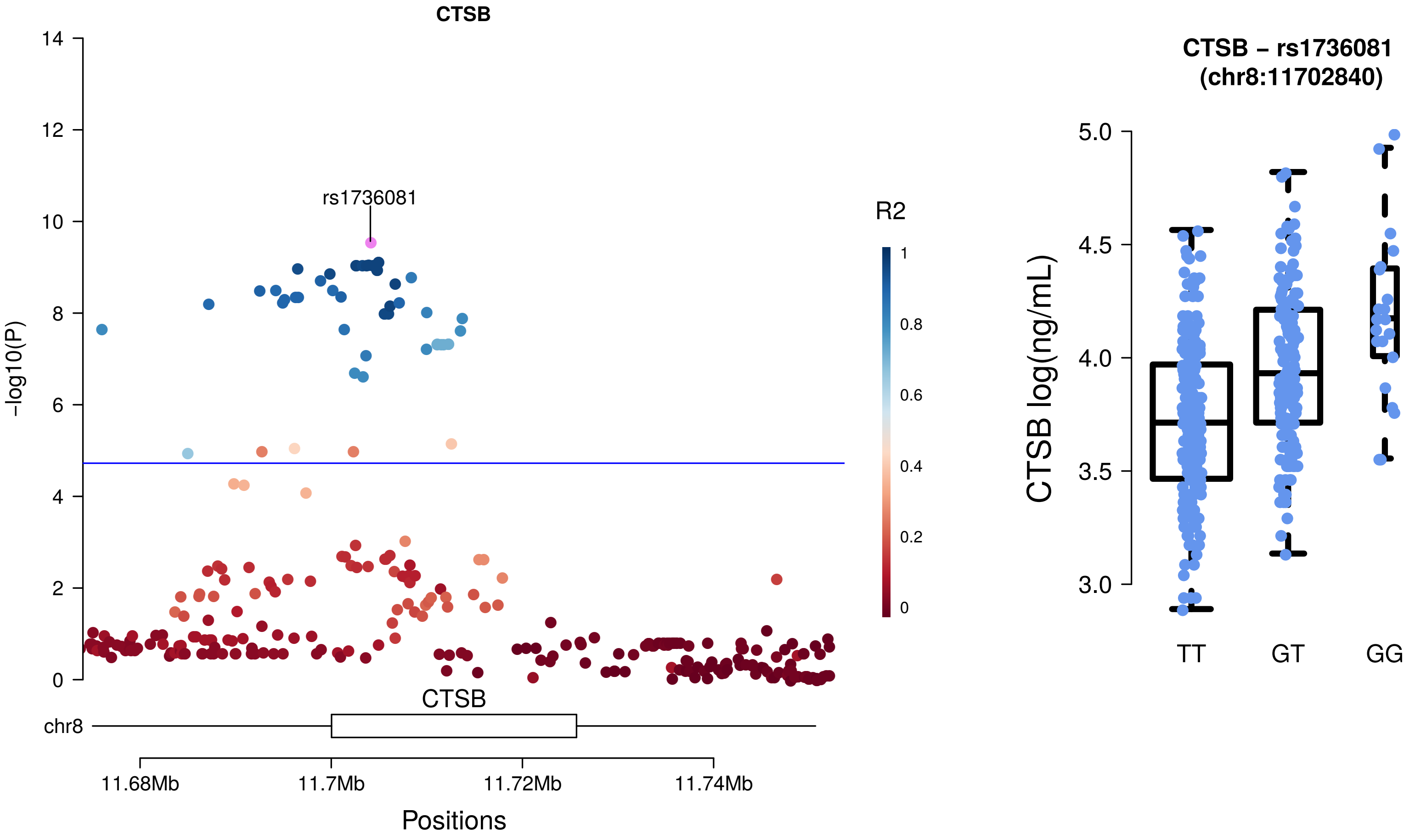
CTSB
rs1736081
R2
-log10(P)
CTSB
chr8
11.68Mb
11.7Mb
11.72Mb
11.74Mb
Positions
CTSB – rs1736081
(chr8:11702840)
CTSB log(ng/mL)
TT
GT
GG

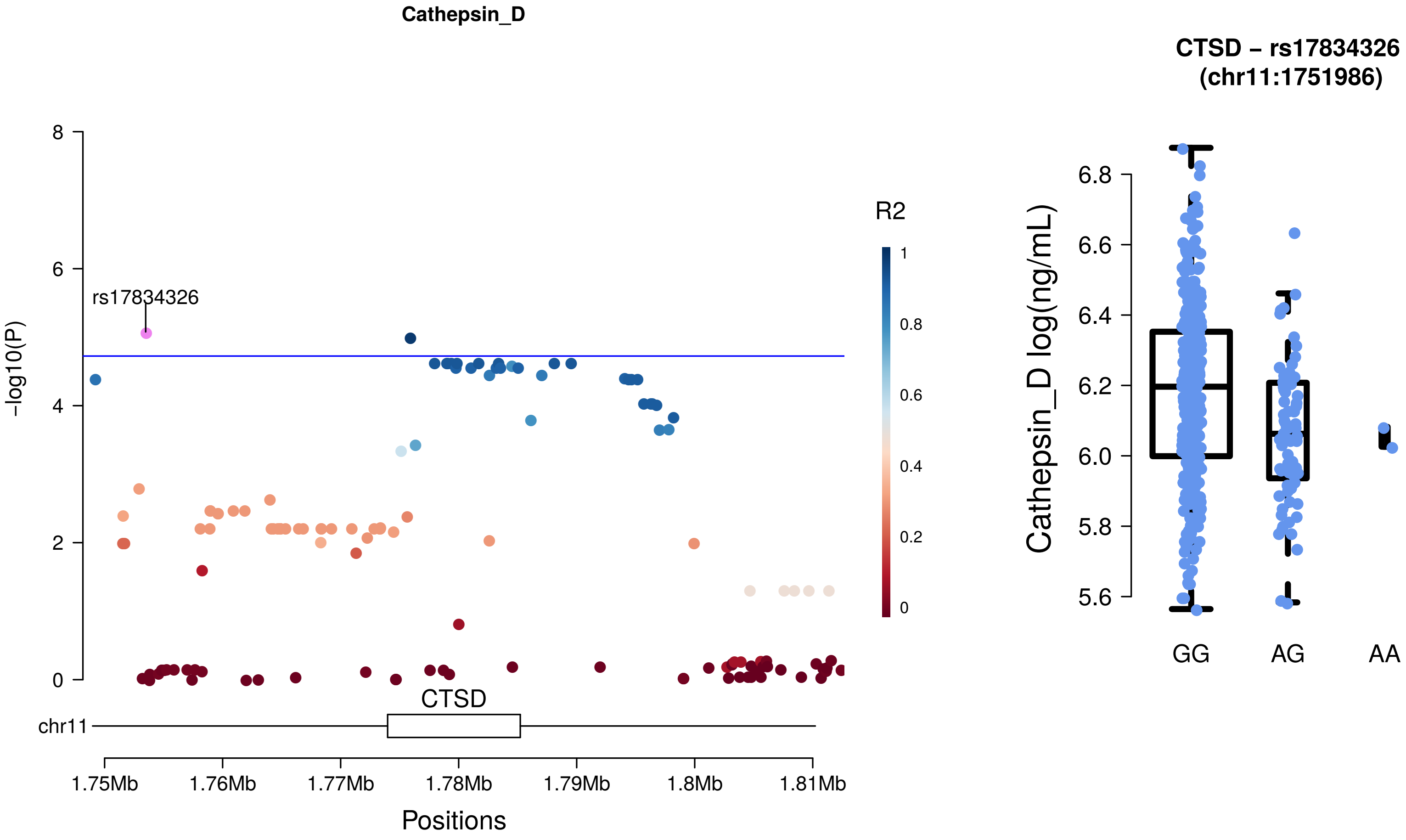

Cathepsin_D
rs17834326
-log10(P)
R2
1
0.8
0.6
0.4
0.2
0
8
6
4
2
0
CTSD
chr11
1.75Mb
1.76Mb
1.77Mb
1.78Mb
1.79Mb
1.8Mb
1.81Mb
Positions
CTSD – rs17834326
(chr11:1751986)
Cathepsin_D log(ng/mL)
6.8
6.6
6.4
6.2
6.0
5.8
5.6
GG
AG
AA

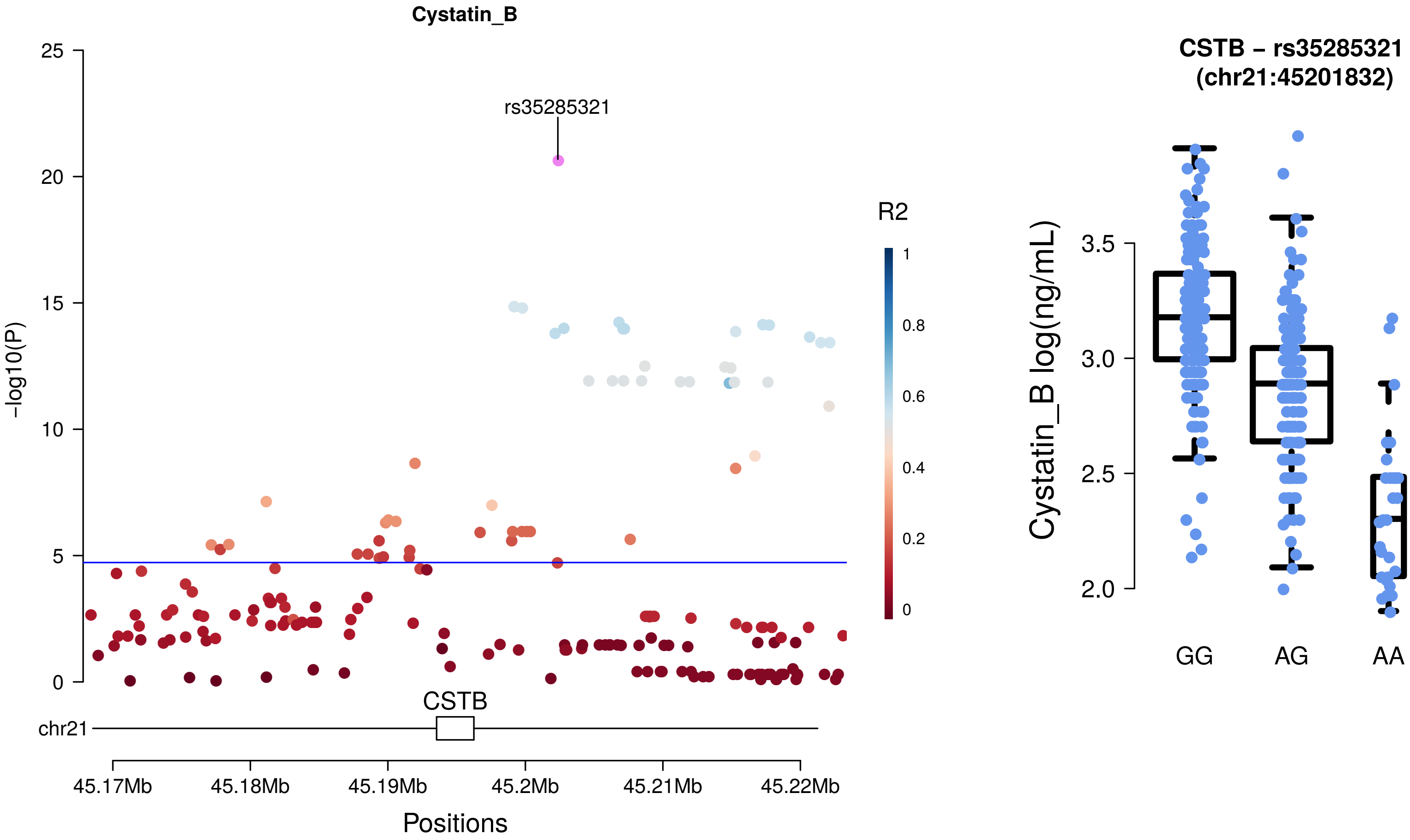

Cystatin_B
rs35285321
-log10(P)
25
20
15
10
5
0
R2
1
0.8
0.6
0.4
0.2
0
CSTB
chr21
45.17Mb
45.18Mb
45.19Mb
45.2Mb
45.21Mb
45.22Mb
Positions
CSTB – rs35285321
(chr21:45201832)
Cystatin_B log(ng/mL)
3.5
3.0
2.5
2.0
GG
AG
AA

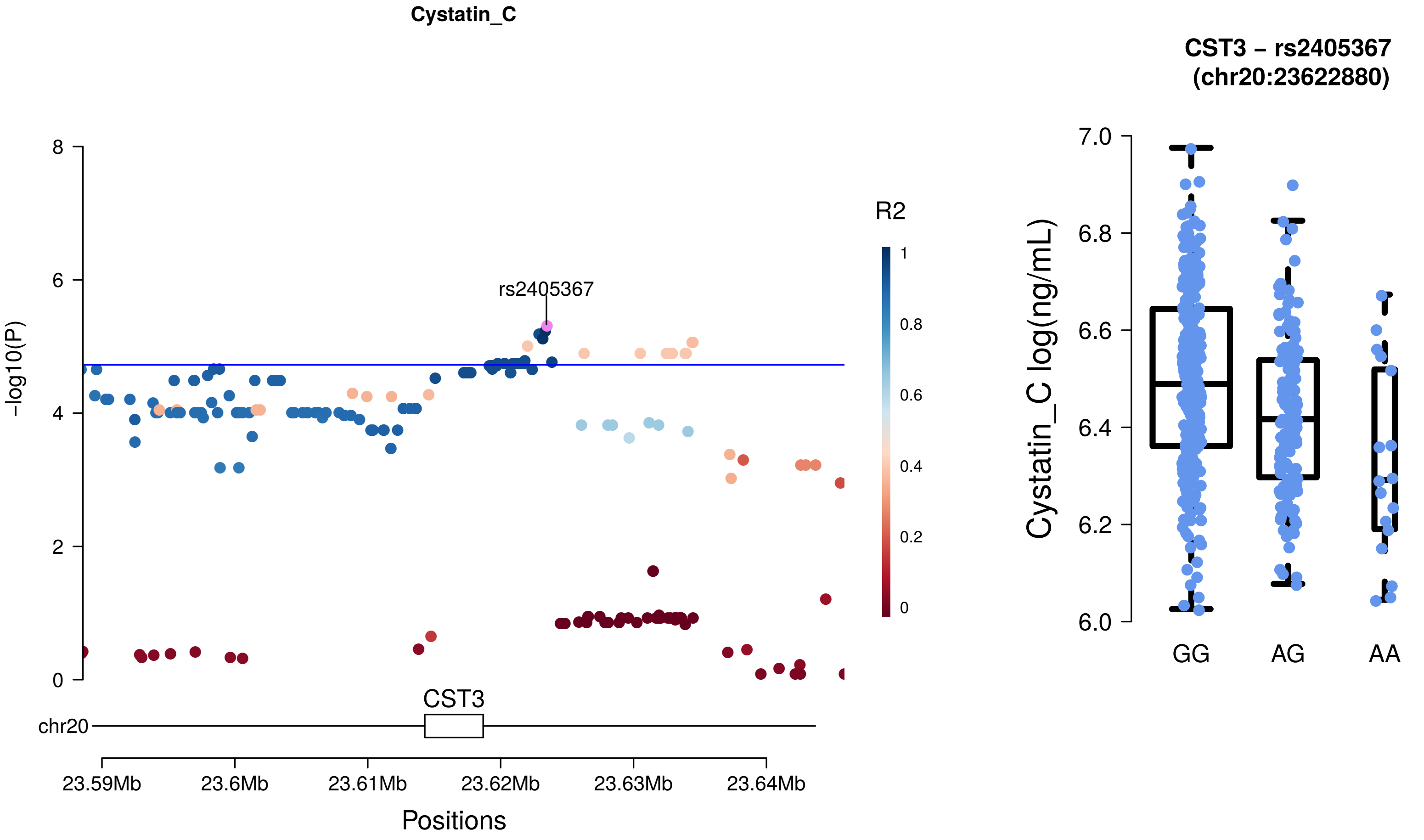

Cystatin_C
-log10(P)
8
6
4
2
0
rs2405367
R2
1
0.8
0.6
0.4
0.2
0
CST3
chr20
23.59Mb
23.6Mb
23.61Mb
23.62Mb
23.63Mb
23.64Mb
Positions
CST3 – rs2405367
(chr20:23622880)
Cystatin_C log(ng/mL)
7.0
6.8
6.6
6.4
6.2
6.0
GG
AG
AA

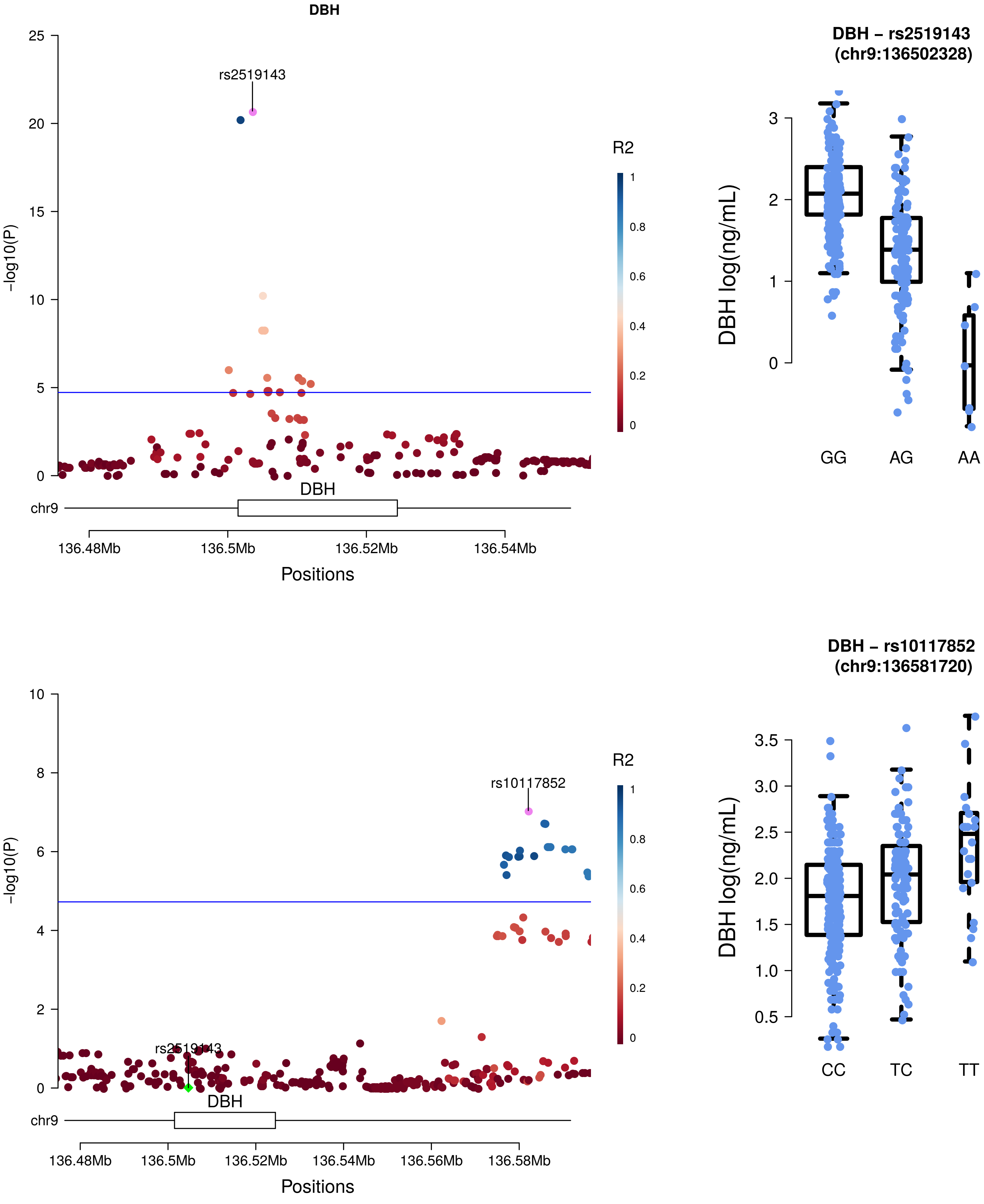
DBH
rs2519143
-log10(P)
R2
DBH
chr9
136.48Mb
136.5Mb
136.52Mb
136.54Mb
Positions
DBH – rs2519143
(chr9:136502328)
DBH log(ng/mL)
GG
AG
AA
rs10117852
rs2519143
DBH
136.56Mb
136.58Mb
DBH – rs10117852
(chr9:136581720)
CC
TC
TT

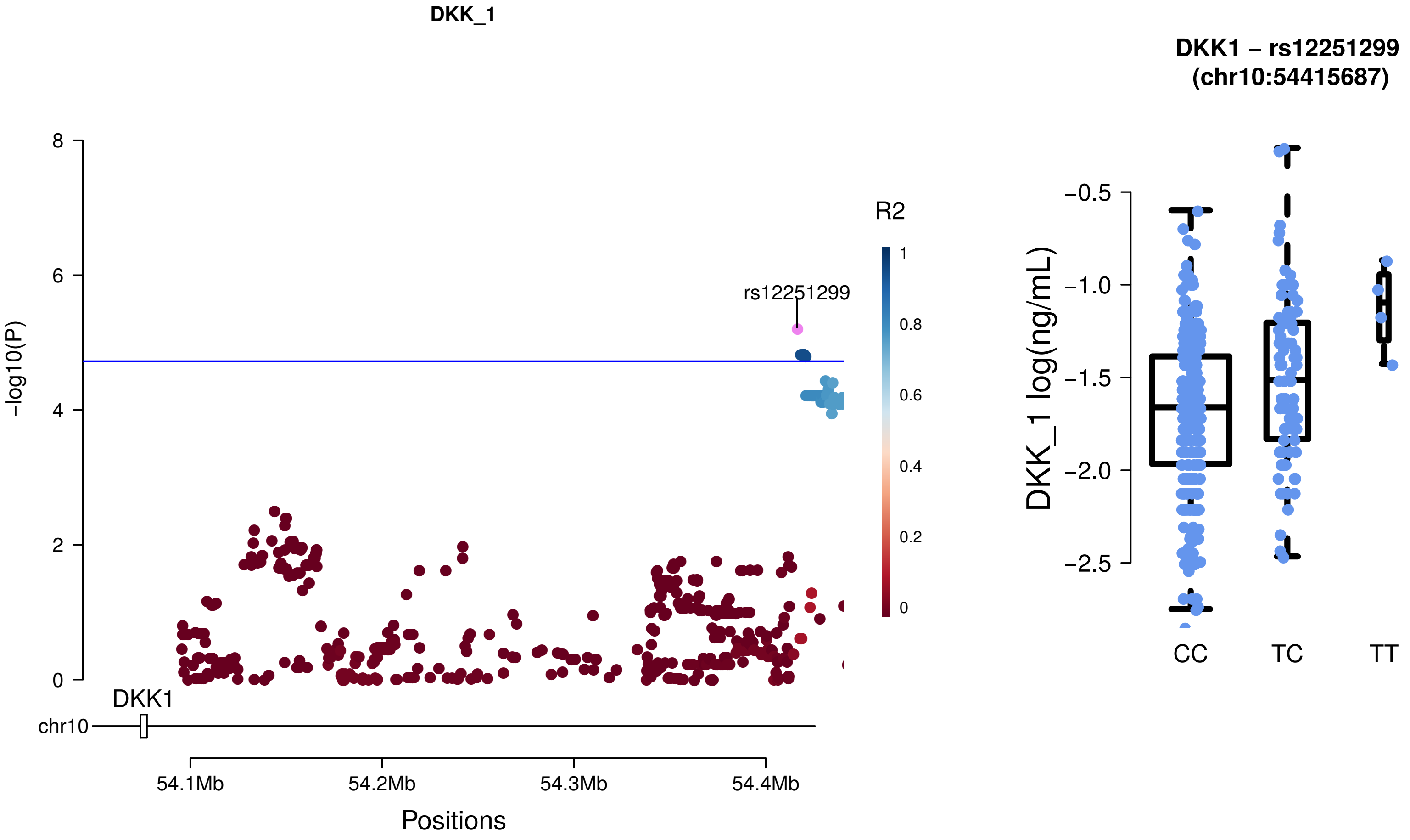

DKK_1
R2
rs12251299
-log10(P)
DKK1
chr10
54.1Mb
54.2Mb
54.3Mb
54.4Mb
Positions
DKK1 – rs12251299
(chr10:54415687)
DKK_1 log(ng/mL)
CC
TC
TT

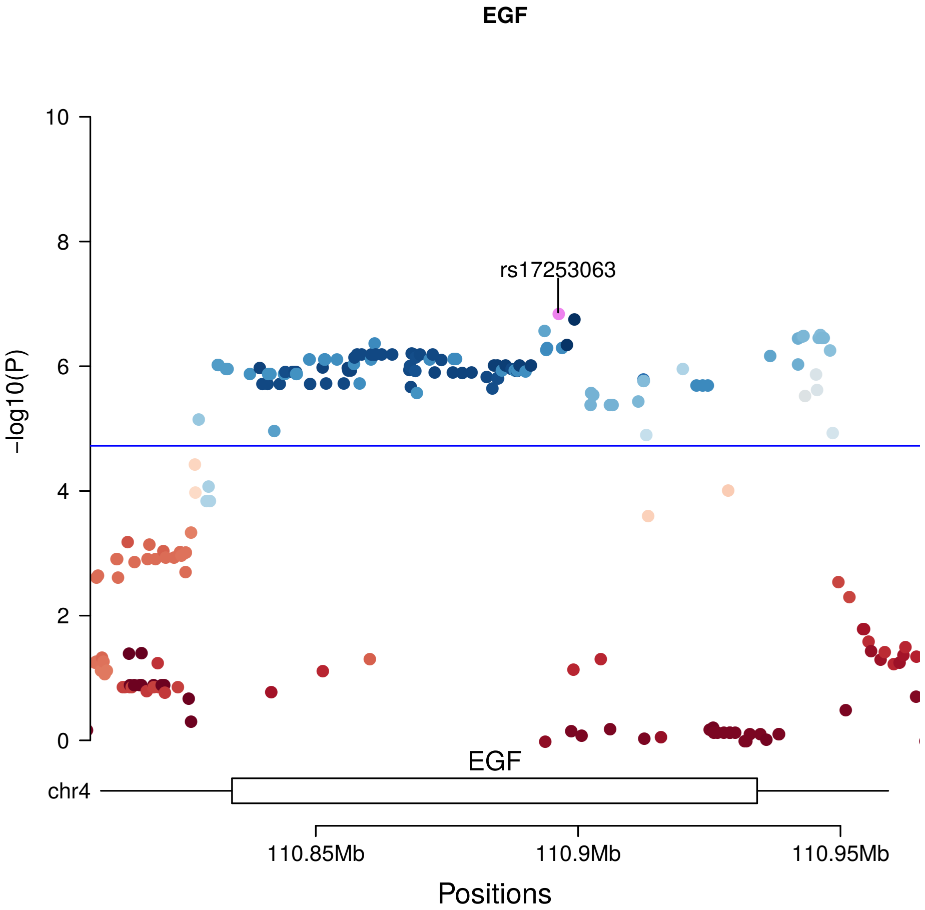
EGF
rs17253063
R2
1
0.8
0.6
0.4
0.2
0
10
8
6
4
2
0
-log10(P)
EGF
chr4
110.85Mb
110.9Mb
110.95Mb
Positions

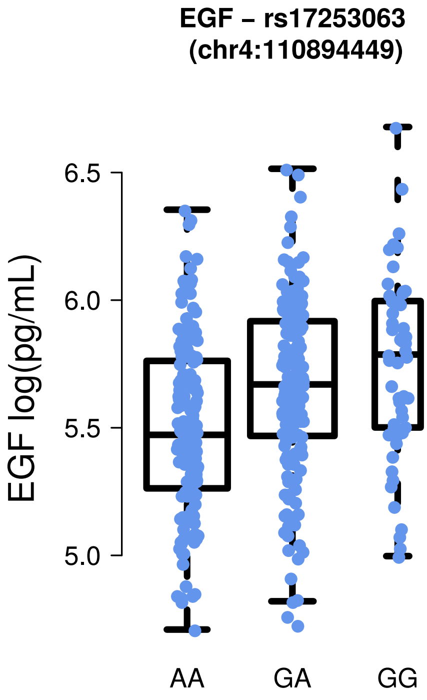
EGF – rs17253063
(chr4:110894449)
EGF log(pg/mL)
6.5
6.0
5.5
5.0
AA
GA
GG

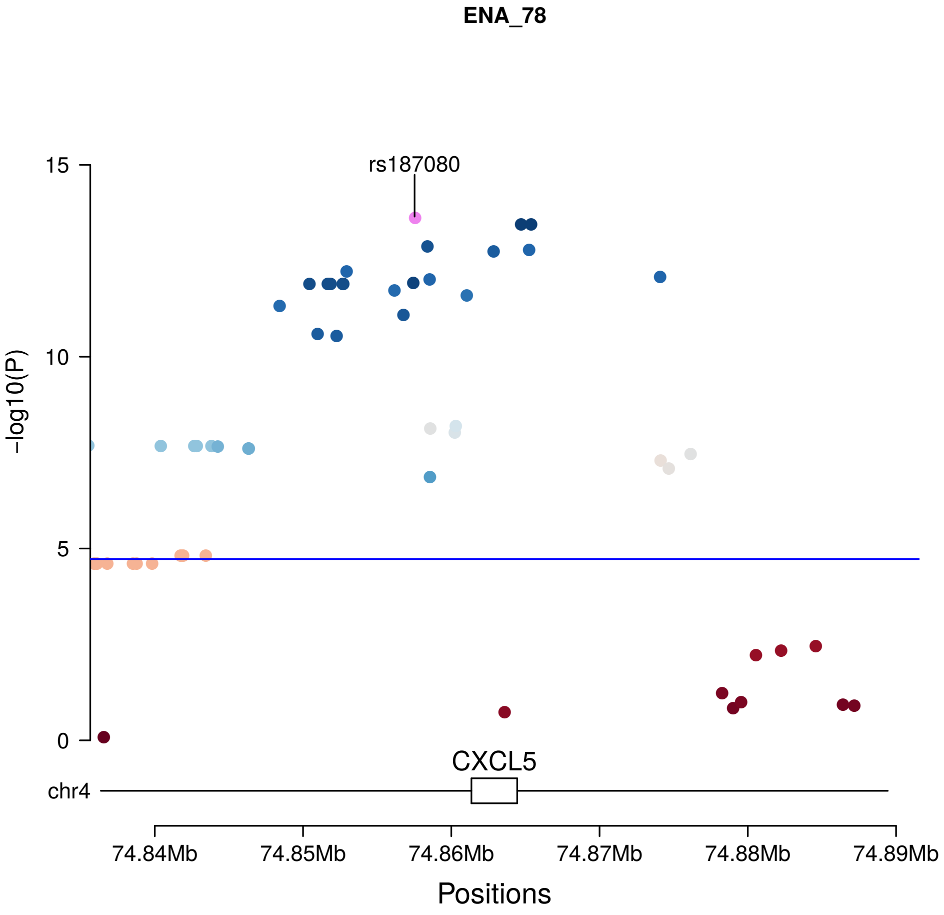
ENA_78
rs187080
15
10
5
0
-log10(P)
R2
1
0.8
0.6
0.4
0.2
0
CXCL5
chr4
74.84Mb
74.85Mb
74.86Mb
74.87Mb
74.88Mb
74.89Mb
Positions

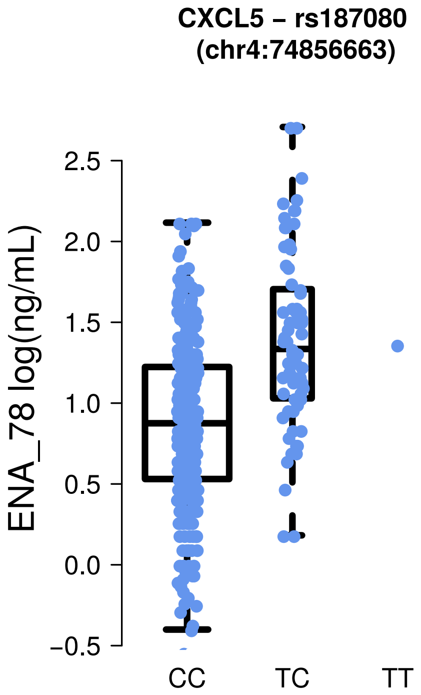
CXCL5 – rs187080
(chr4:74856663)
ENA_78 log(ng/mL)
2.5
2.0
1.5
1.0
0.5
0.0
−0.5
CC
TC
TT

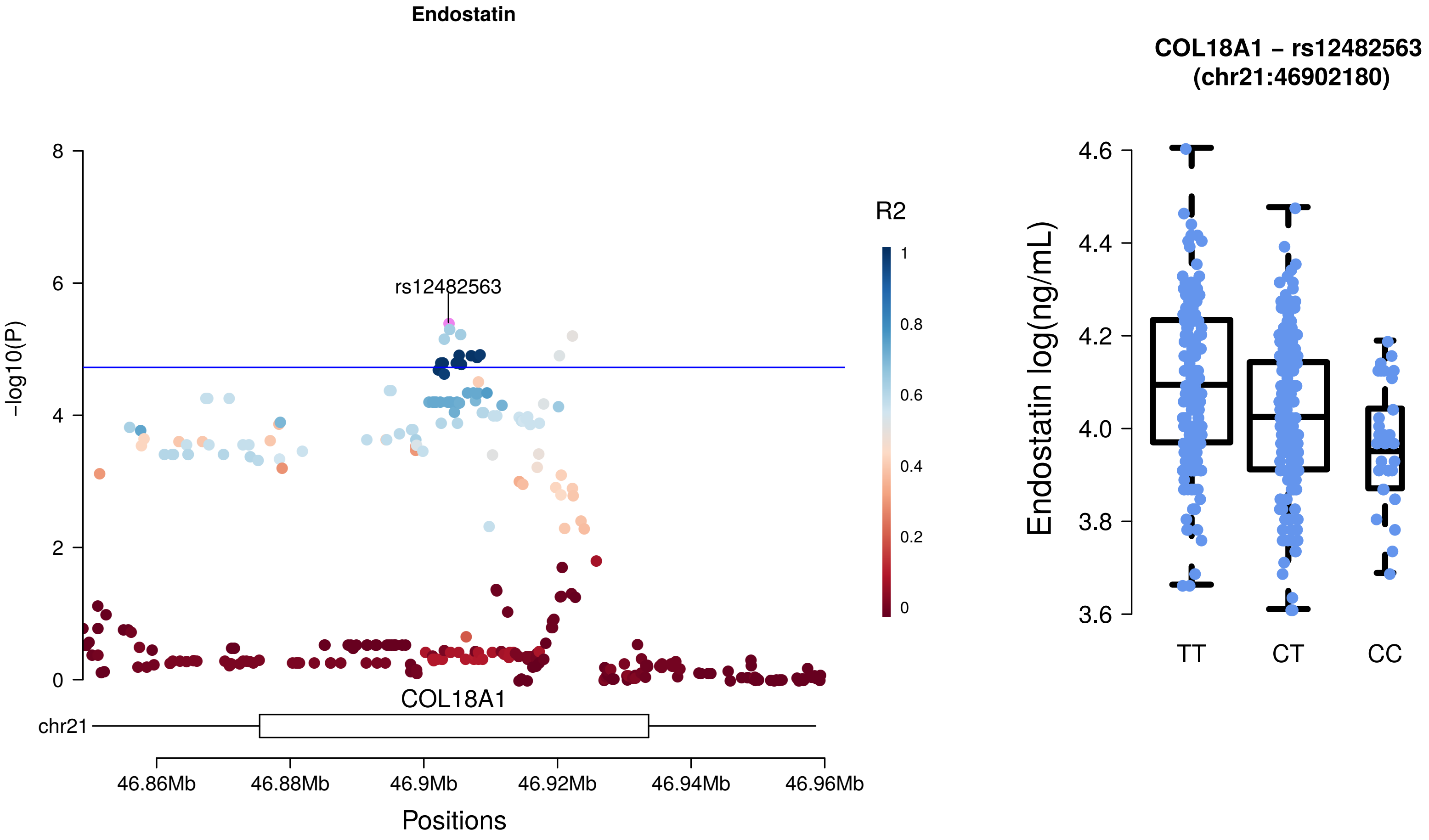

Endostatin
rs12482563
R2
1
0.8
0.6
0.4
0.2
0
8
6
4
2
0
-log10(P)
COL18A1
chr21
46.86Mb
46.88Mb
46.9Mb
46.92Mb
46.94Mb
46.96Mb
Positions
COL18A1 – rs12482563
(chr21:46902180)
Endostatin log(ng/mL)
4.6
4.4
4.2
4.0
3.8
3.6
TT
CT
CC

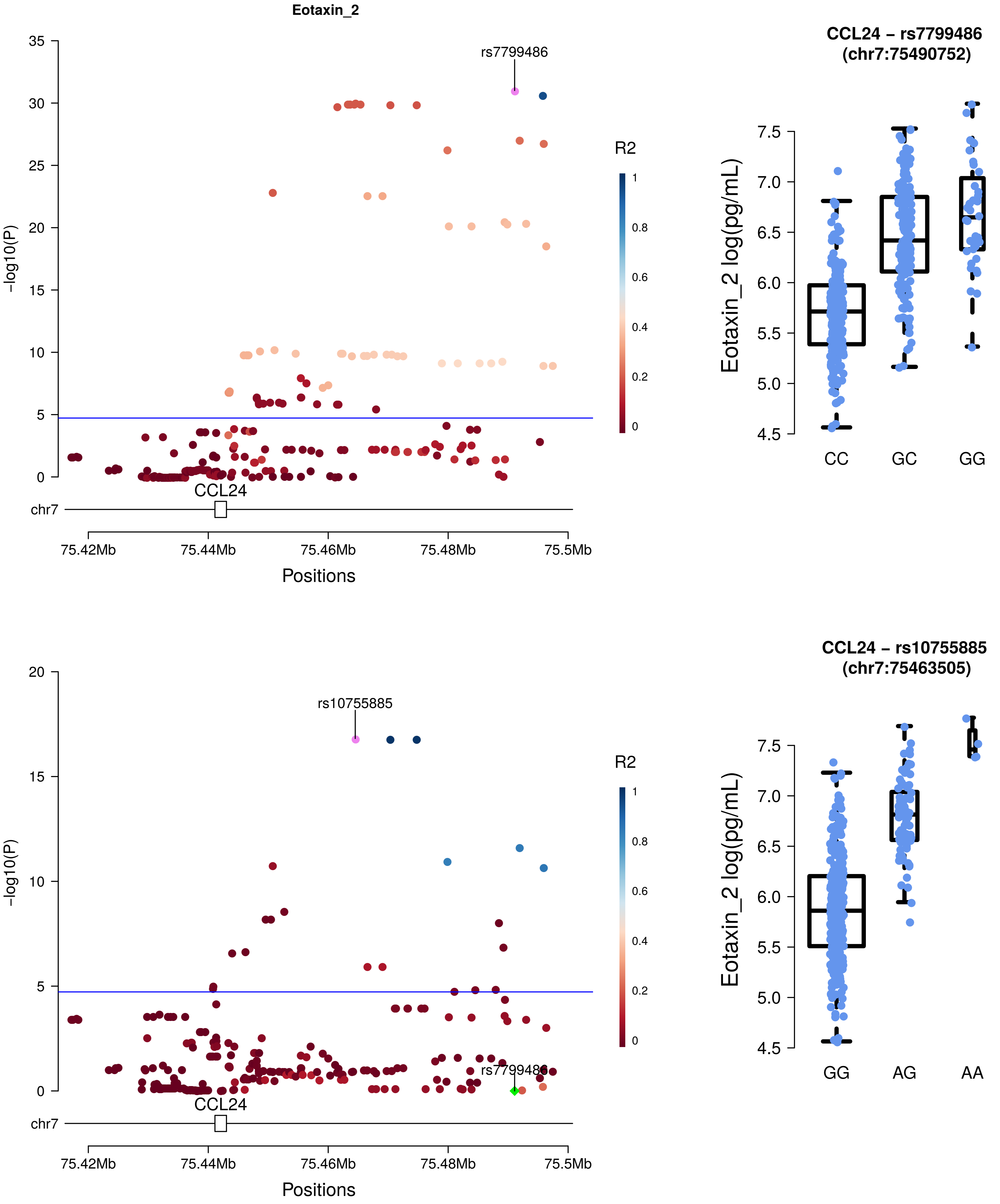

Eotaxin_2
rs7799486
R2
-log10(P)
CCL24
chr7
75.42Mb
75.44Mb
75.46Mb
75.48Mb
75.5Mb
Positions
CCL24 – rs7799486
(chr7:75490752)
Eotaxin_2 log(pg/mL)
CC
GC
GG
rs10755885
CCL24 – rs10755885
(chr7:75463505)
GG
AG
AA

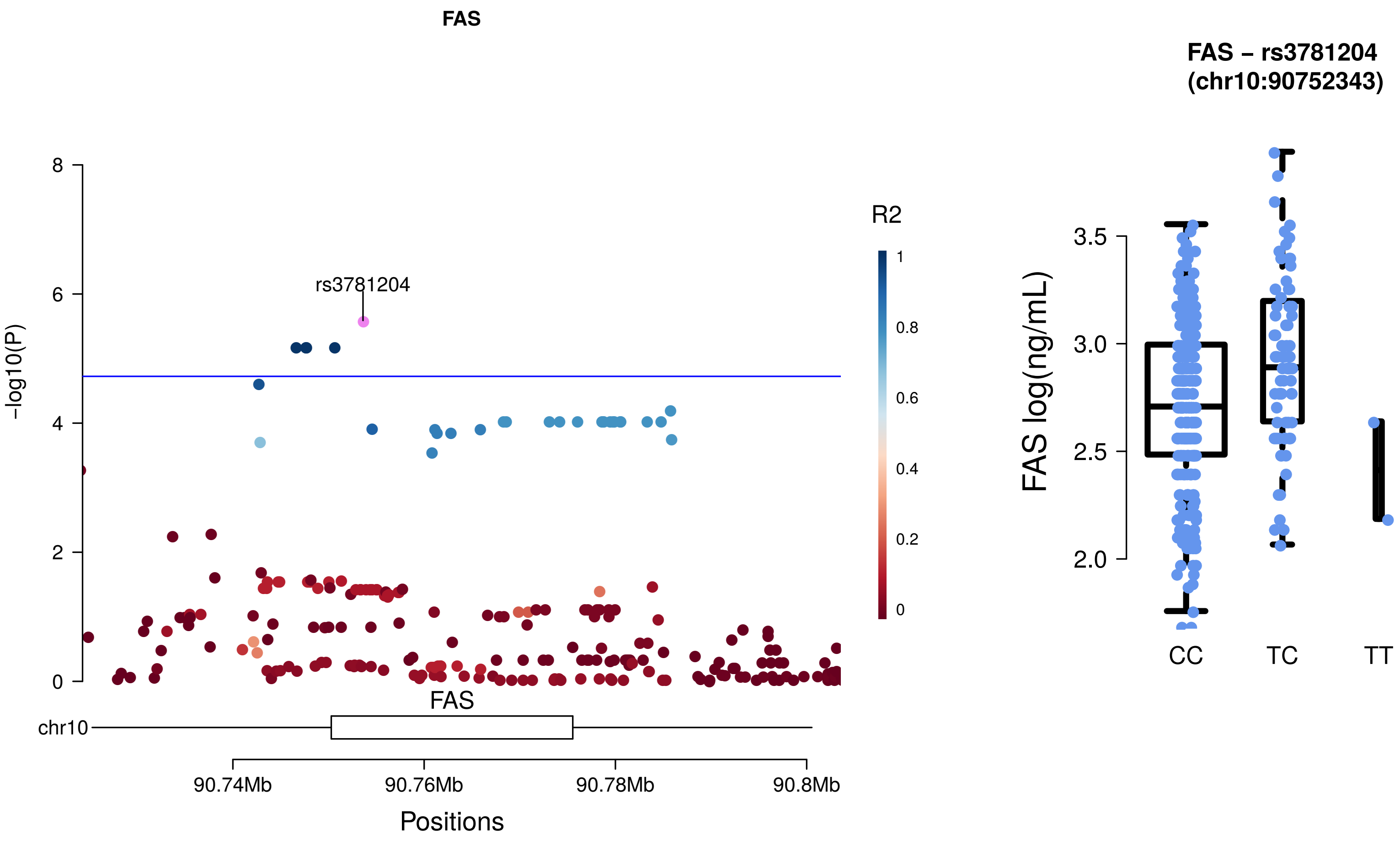

FAS
rs3781204
R2
1
0.8
0.6
0.4
0.2
0
-log10(P)
8
6
4
2
0
chr10
90.74Mb
90.76Mb
90.78Mb
90.8Mb
Positions
FAS – rs3781204
(chr10:90752343)
FAS log(ng/mL)
3.5
3.0
2.5
2.0
CC
TC
TT

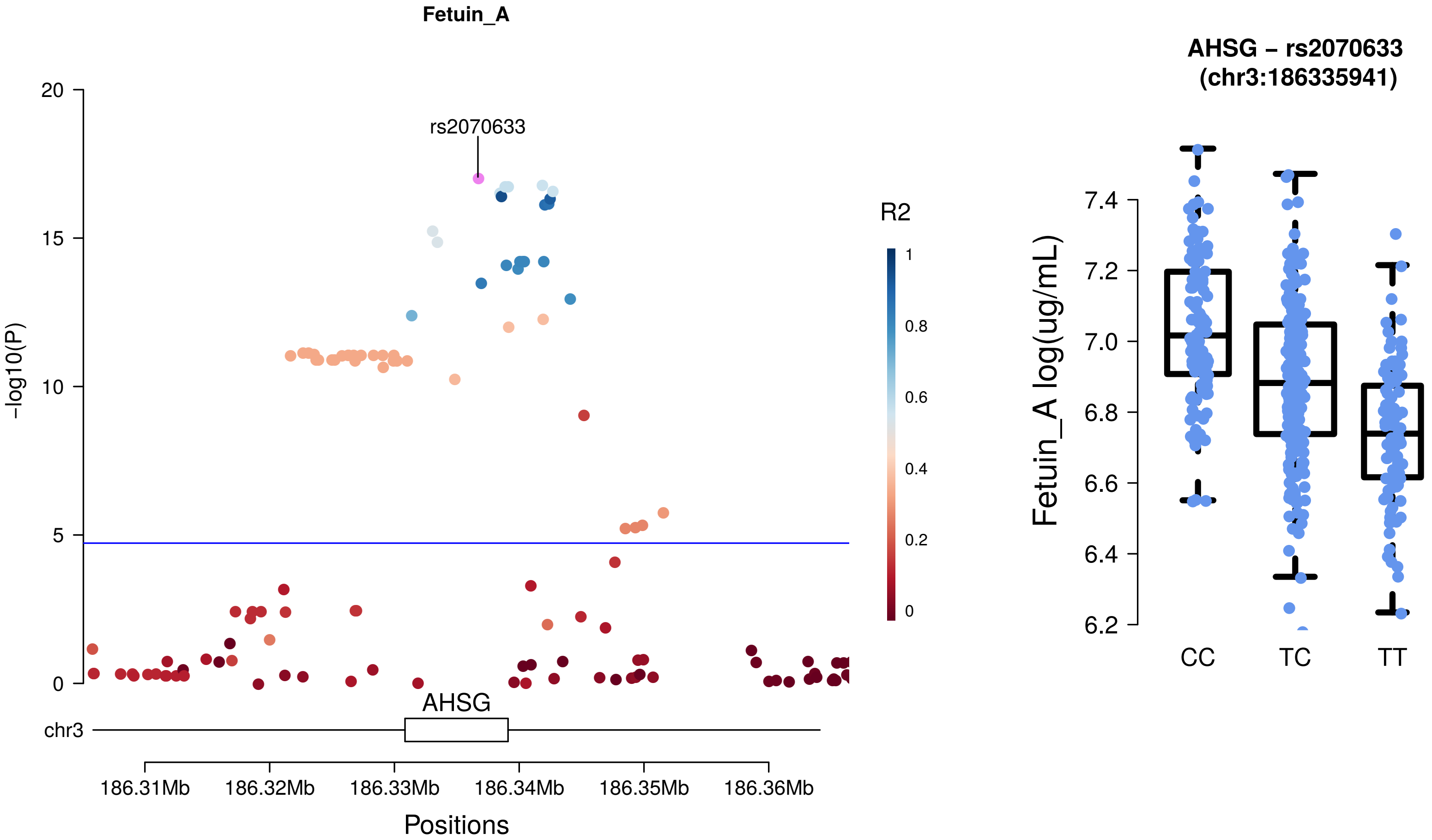

Fetuin_A
rs2070633
R2
1
0.8
0.6
0.4
0.2
0
20
15
10
5
0
-log10(P)
AHSG
chr3
186.31Mb
186.32Mb
186.33Mb
186.34Mb
186.35Mb
186.36Mb
Positions
AHSG – rs2070633
(chr3:186335941)
Fetuin_A log(ug/mL)
7.4
7.2
7.0
6.8
6.6
6.4
6.2
CC
TC
TT

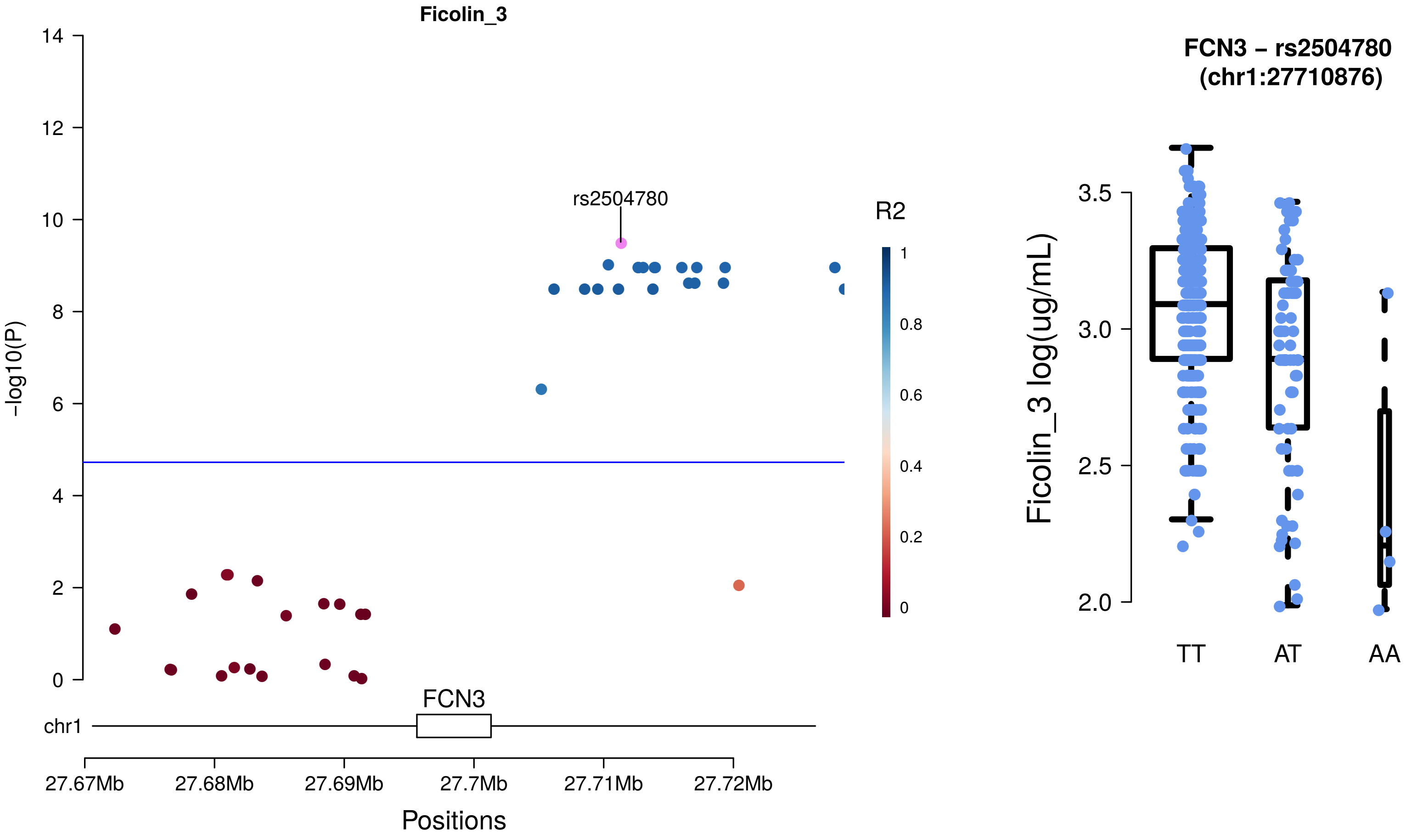
Ficolin_3
rs2504780
R2
-log10(P)
FCN3
chr1
27.67Mb
27.68Mb
27.69Mb
27.7Mb
27.71Mb
27.72Mb
Positions
FCN3 – rs2504780
(chr1:27710876)
Ficolin_3 log(ug/mL)
TT
AT
AA

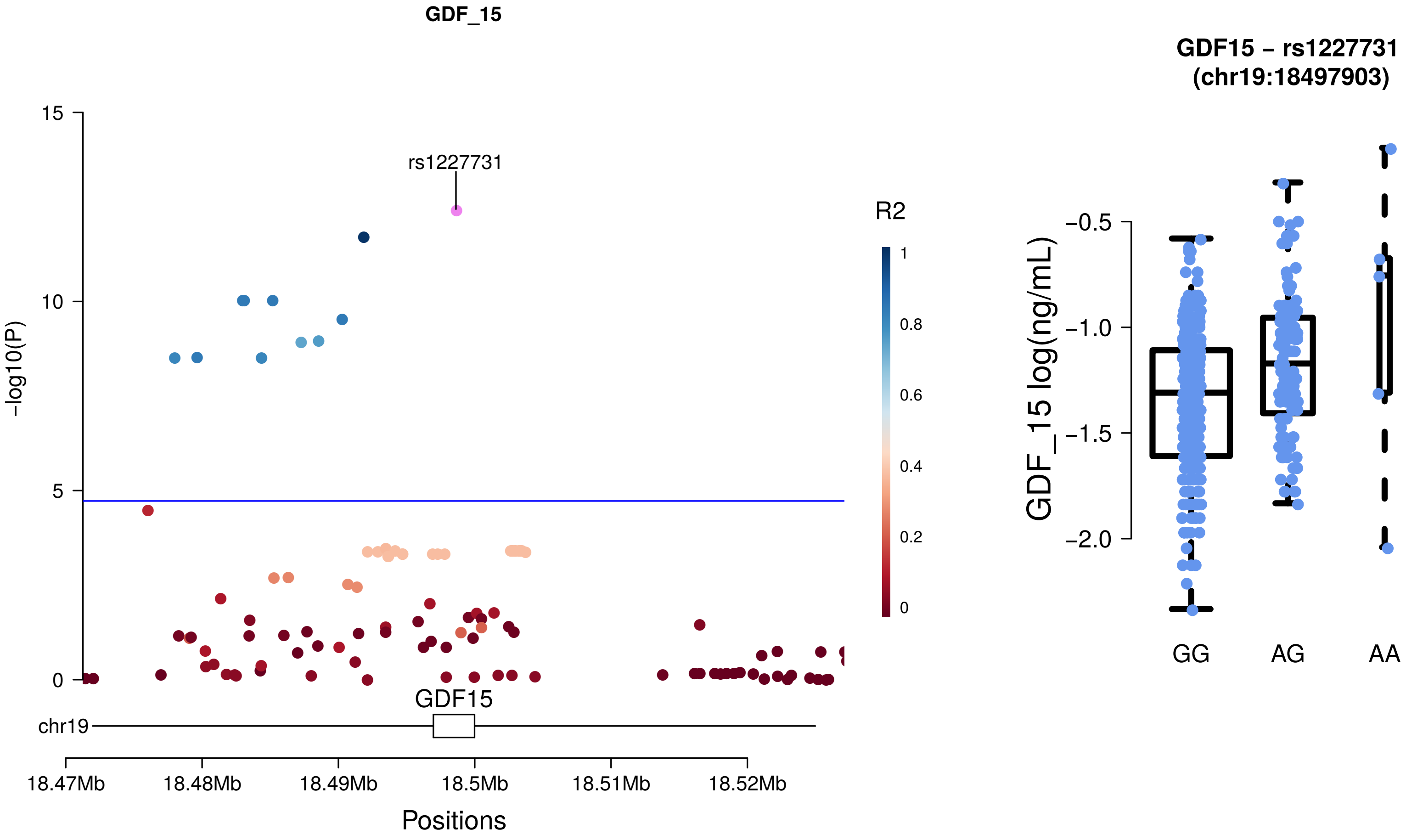
GDF_15
rs1227731
-log10(P)
15
10
5
0
R2
1
0.8
0.6
0.4
0.2
0
GDF15
chr19
18.47Mb
18.48Mb
18.49Mb
18.5Mb
18.51Mb
18.52Mb
Positions
GDF15 – rs1227731
(chr19:18497903)
GDF_15 log(ng/mL)
−0.5
−1.0
−1.5
−2.0
GG
AG
AA

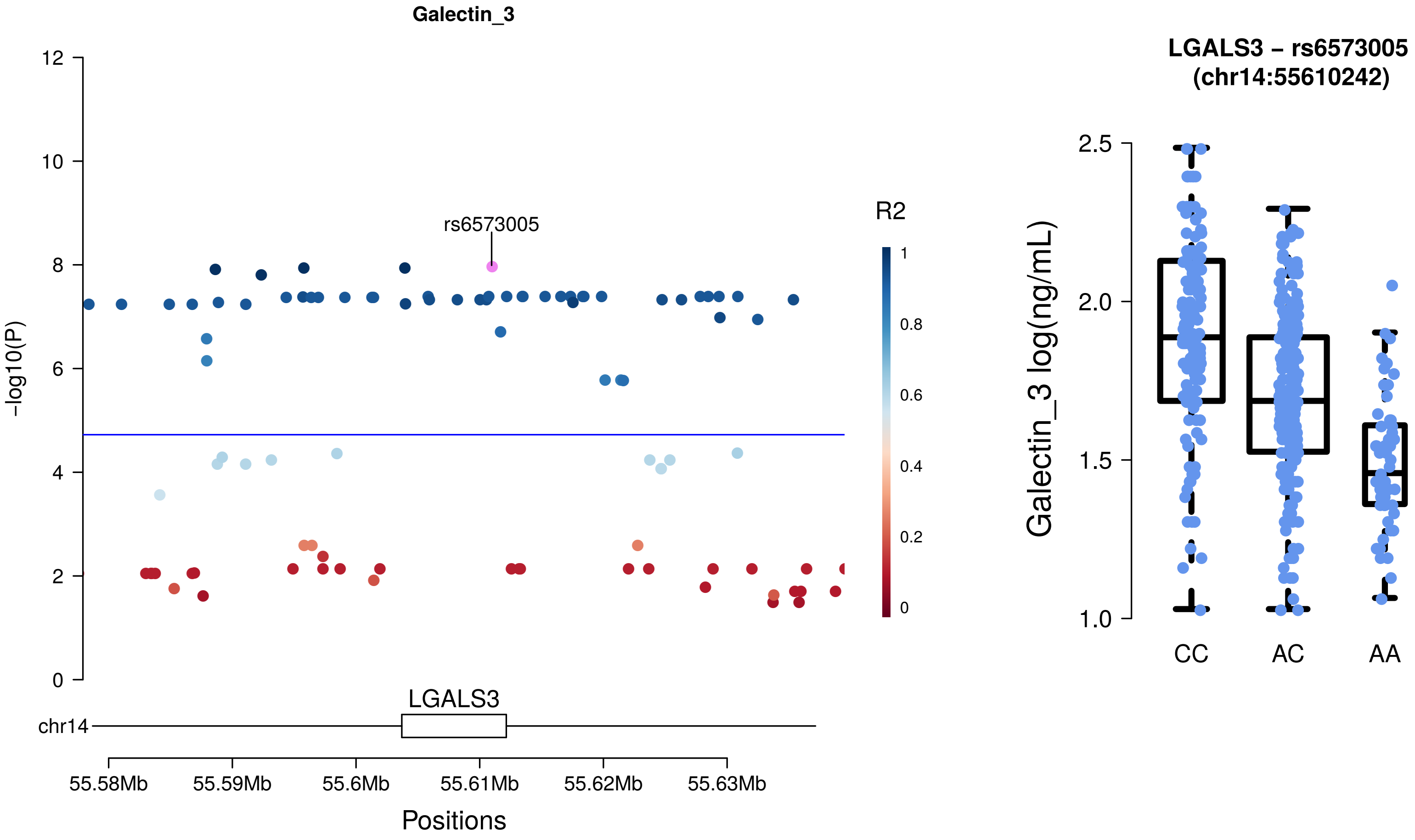
Galectin_3
rs6573005
R2
-log10(P)
LGALS3
chr14
55.58Mb
55.59Mb
55.6Mb
55.61Mb
55.62Mb
55.63Mb
Positions
LGALS3 – rs6573005
(chr14:55610242)
Galectin_3 log(ng/mL)
CC
AC
AA

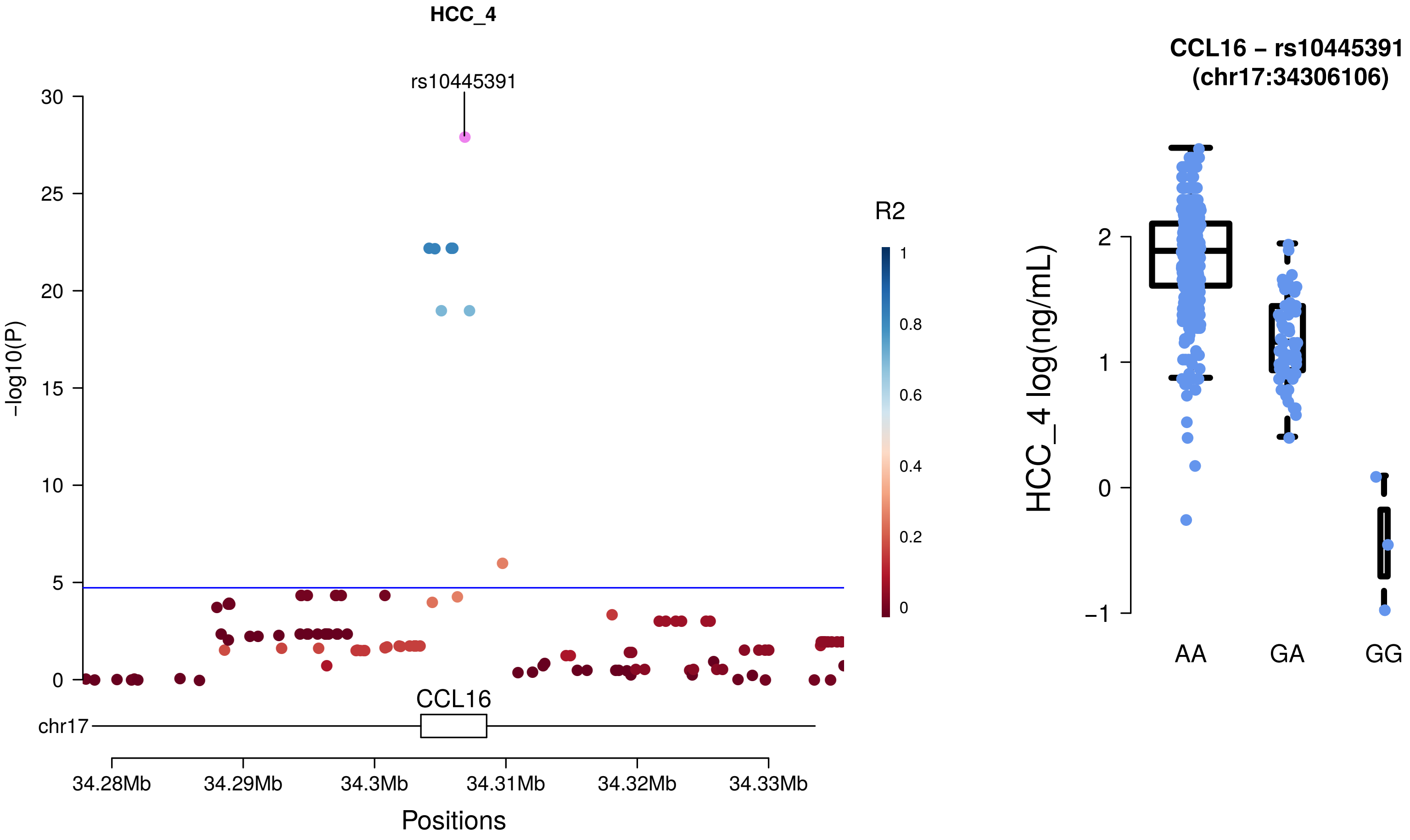
HCC_4
rs10445391
-log10(P)
30
25
20
15
10
5
0
R2
1
0.8
0.6
0.4
0.2
0
CCL16
chr17
34.28Mb
34.29Mb
34.3Mb
34.31Mb
34.32Mb
34.33Mb
Positions
CCL16 – rs10445391
(chr17:34306106)
HCC_4 log(ng/mL)
2
1
0
−1
AA
GA
GG

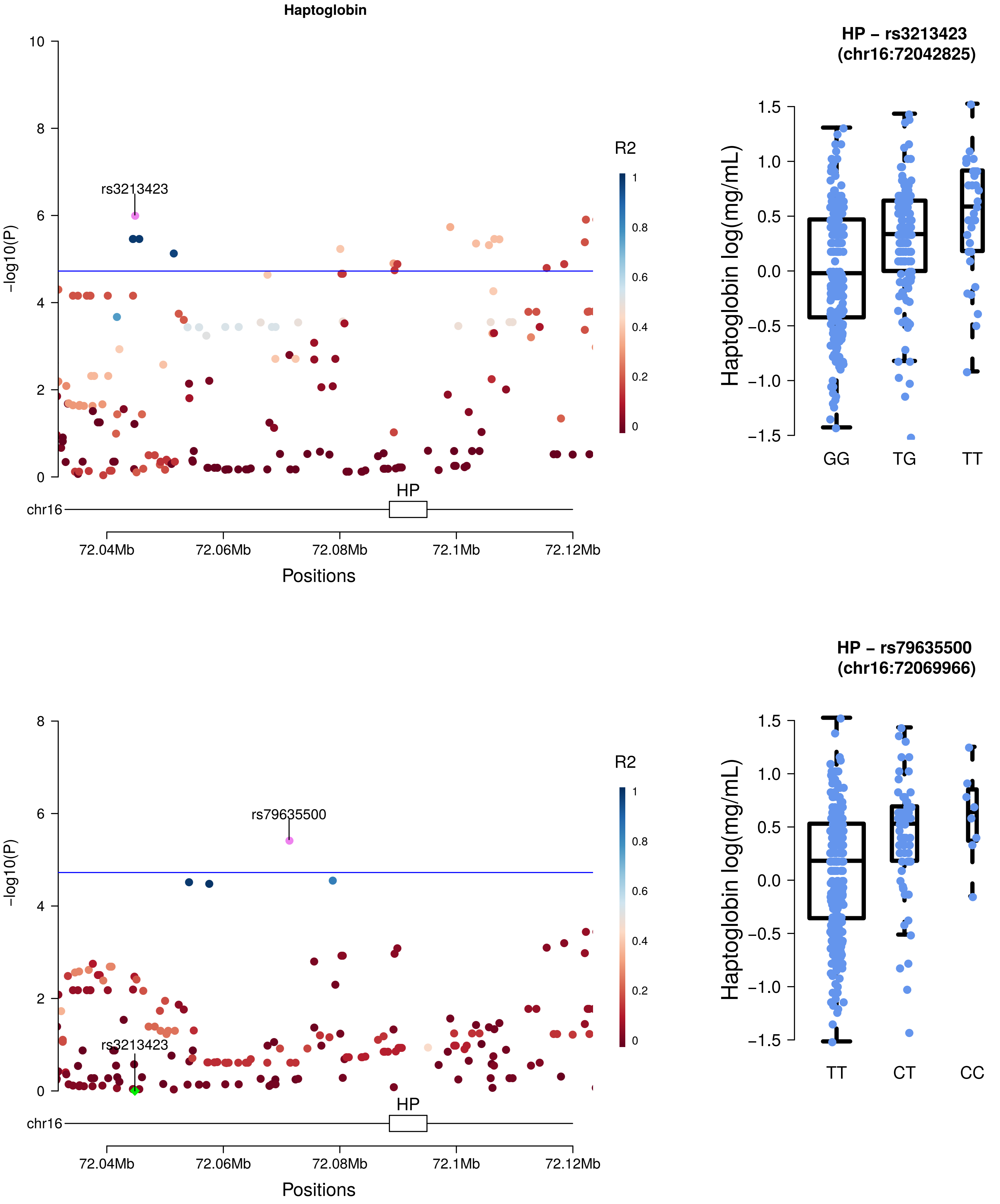
Haptoglobin
rs3213423
-log10(P)
R2
HP
chr16
72.04Mb
72.06Mb
72.08Mb
72.1Mb
72.12Mb
Positions
HP – rs3213423
(chr16:72042825)
Haptoglobin log(mg/mL)
GG
TG
TT
rs79635500
HP – rs79635500
(chr16:72069966)
TT
CT
CC

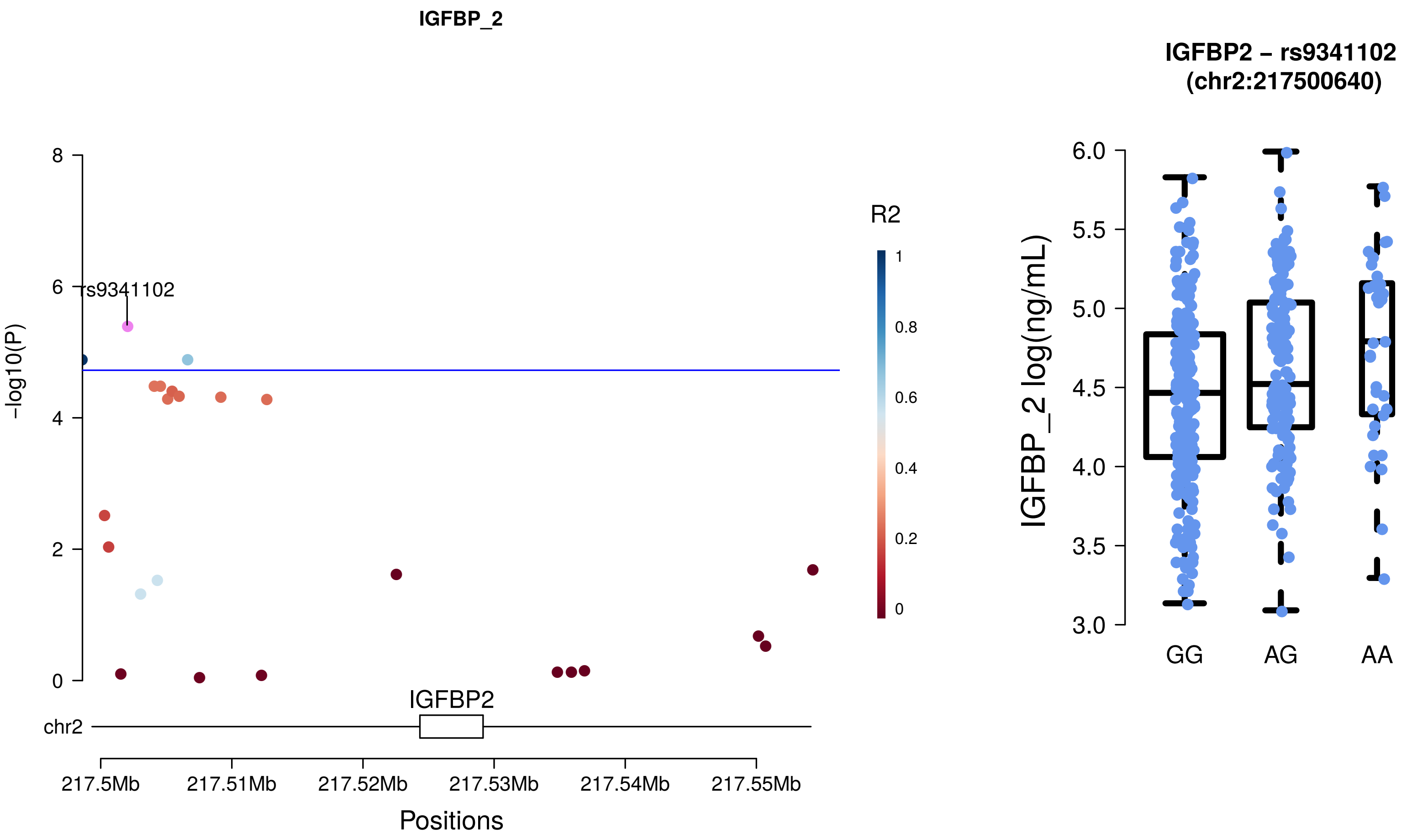
IGFBP_2
IGFBP2 – rs9341102
(chr2:217500640)
-log10(P)
rs9341102
R2
IGFBP2
chr2
217.5Mb
217.51Mb
217.52Mb
217.53Mb
217.54Mb
217.55Mb
Positions
IGFBP_2 log(ng/mL)
GG
AG
AA

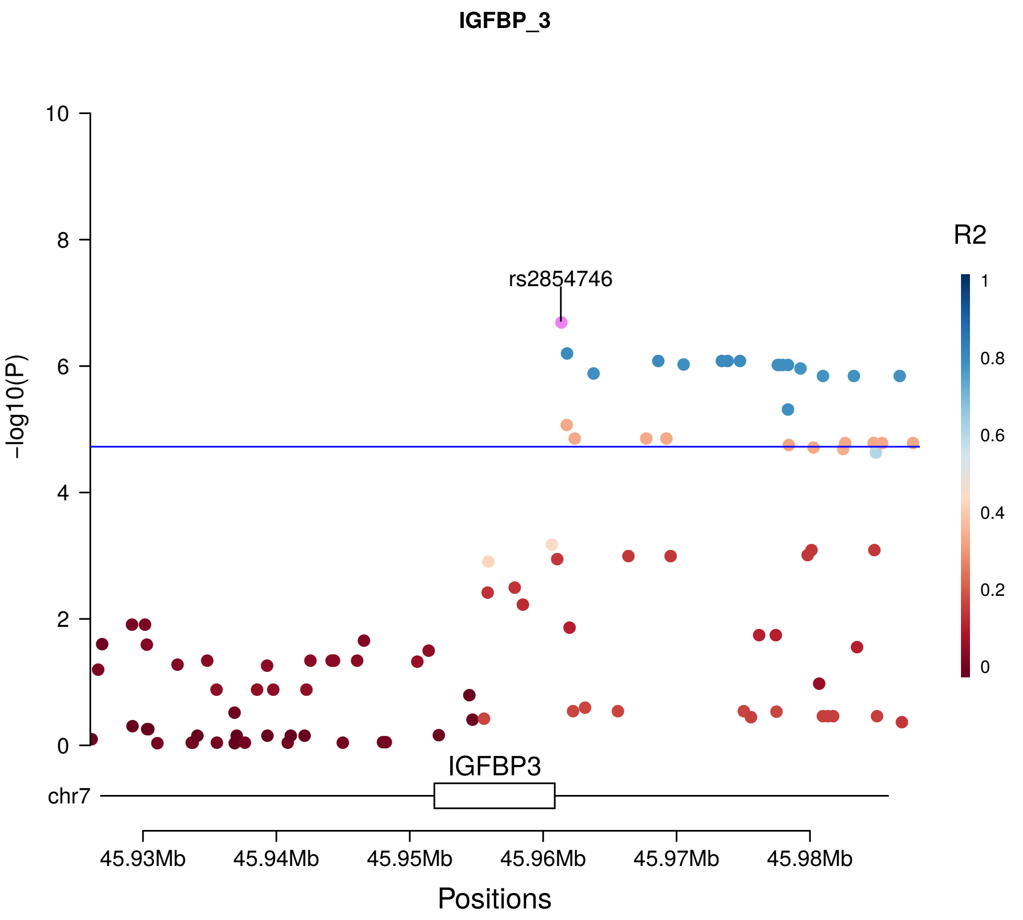

IGFBP_3
rs2854746
R2
-log10(P)
IGFBP3
chr7
45.93Mb
45.94Mb
45.95Mb
45.96Mb
45.97Mb
45.98Mb
Positions

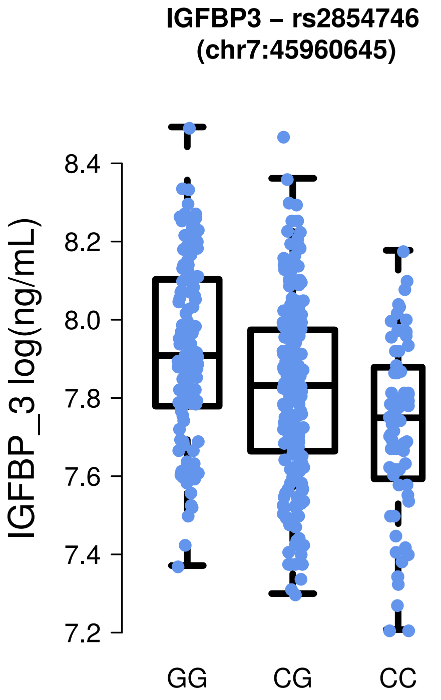

IGFBP3 – rs2854746
(chr7:45960645)
IGFBP_3 log(ng/mL)
GG
CG
CC

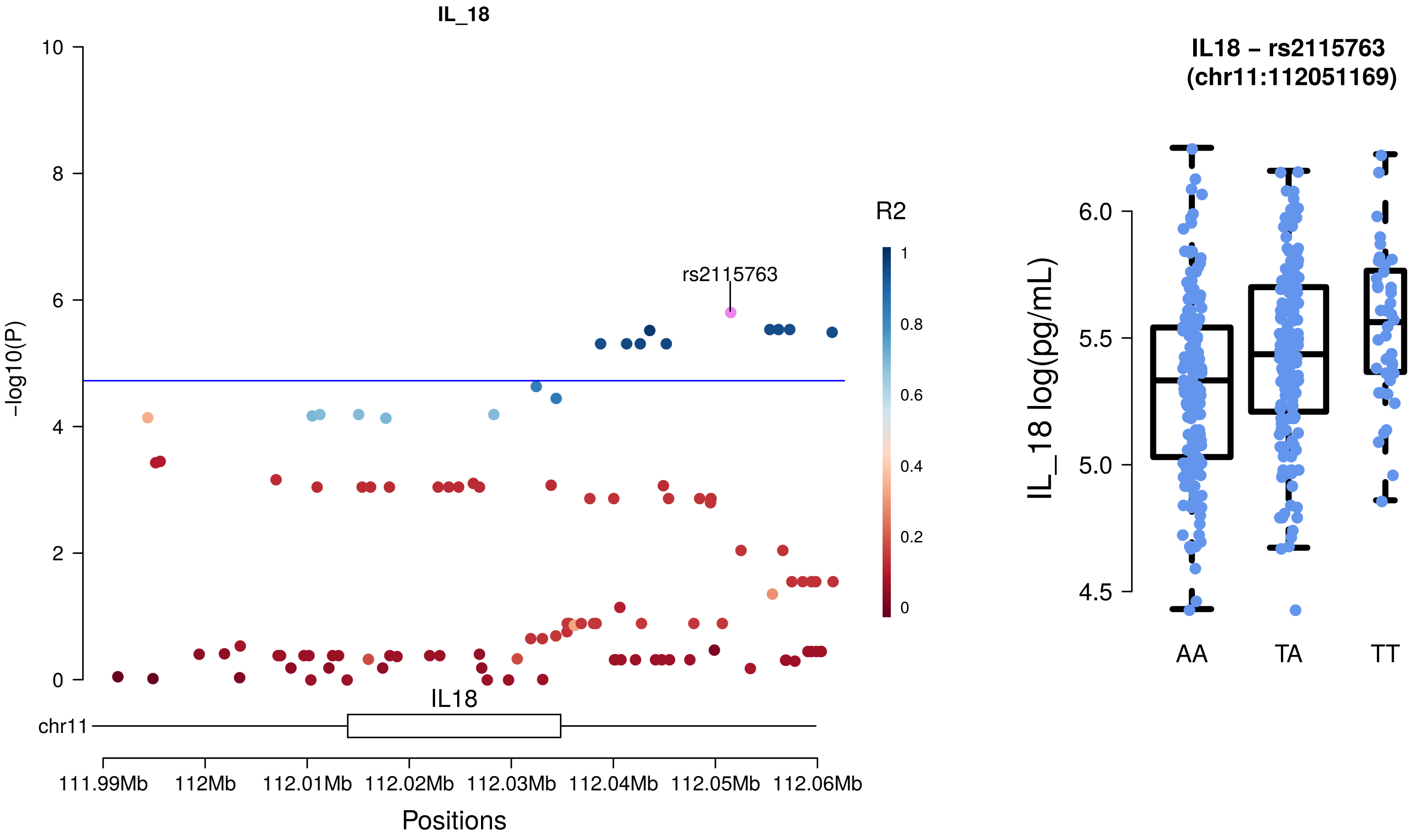
IL_18
-log10(P)
10
8
6
4
2
0
rs2115763
R2
1
0.8
0.6
0.4
0.2
0
IL18
chr11
111.99Mb
112Mb
112.01Mb
112.02Mb
112.03Mb
112.04Mb
112.05Mb
112.06Mb
Positions
IL18 – rs2115763
(chr11:112051169)
IL_18 log(pg/mL)
6.0
5.5
5.0
4.5
AA
TA
TT

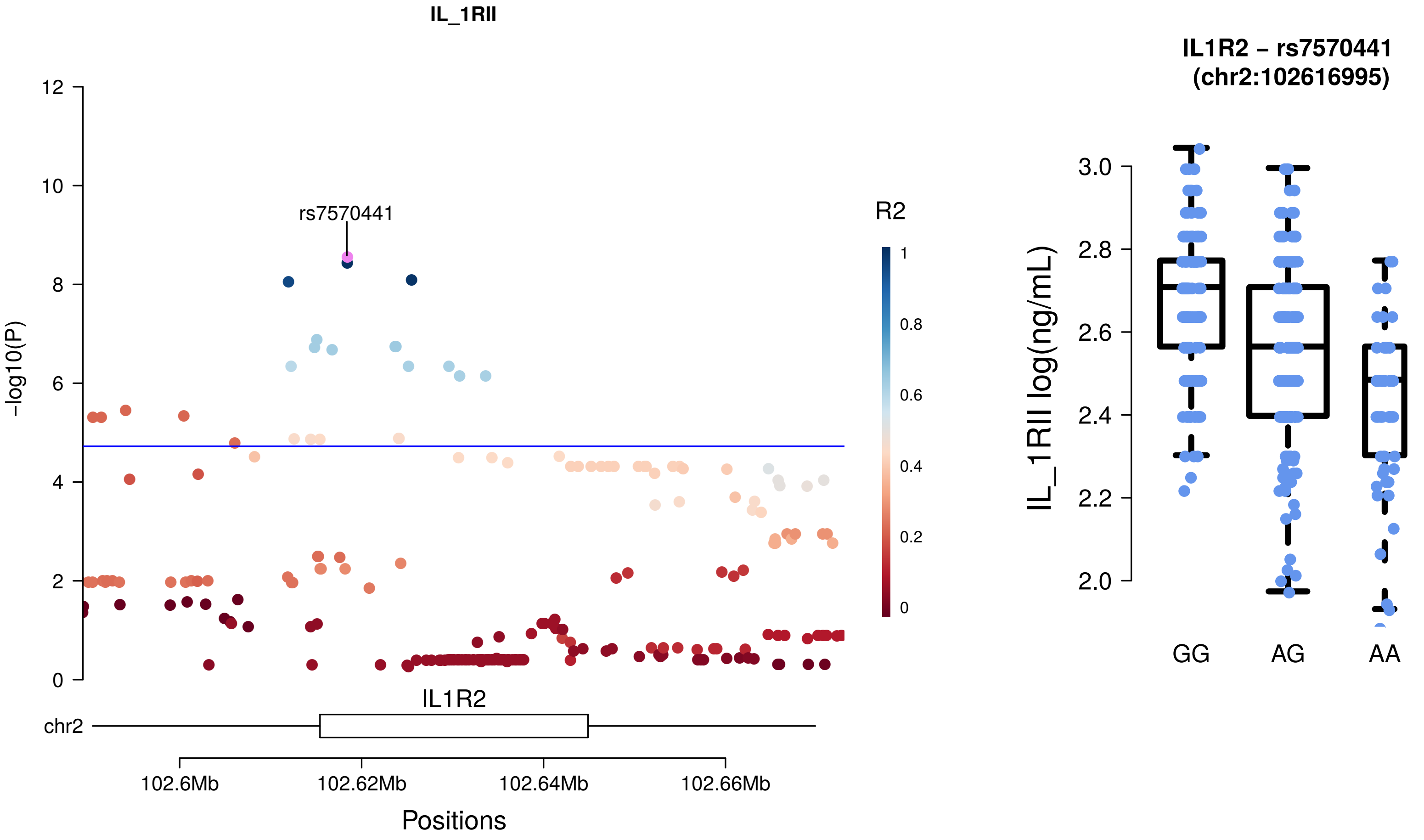
IL_1RII
rs7570441
R2
1
0.8
0.6
0.4
0.2
0
12
10
8
6
4
2
0
-log10(P)
IL1R2
chr2
102.6Mb
102.62Mb
102.64Mb
102.66Mb
Positions
IL1R2 – rs7570441
(chr2:102616995)
IL_1RII log(ng/mL)
3.0
2.8
2.6
2.4
2.2
2.0
GG
AG
AA

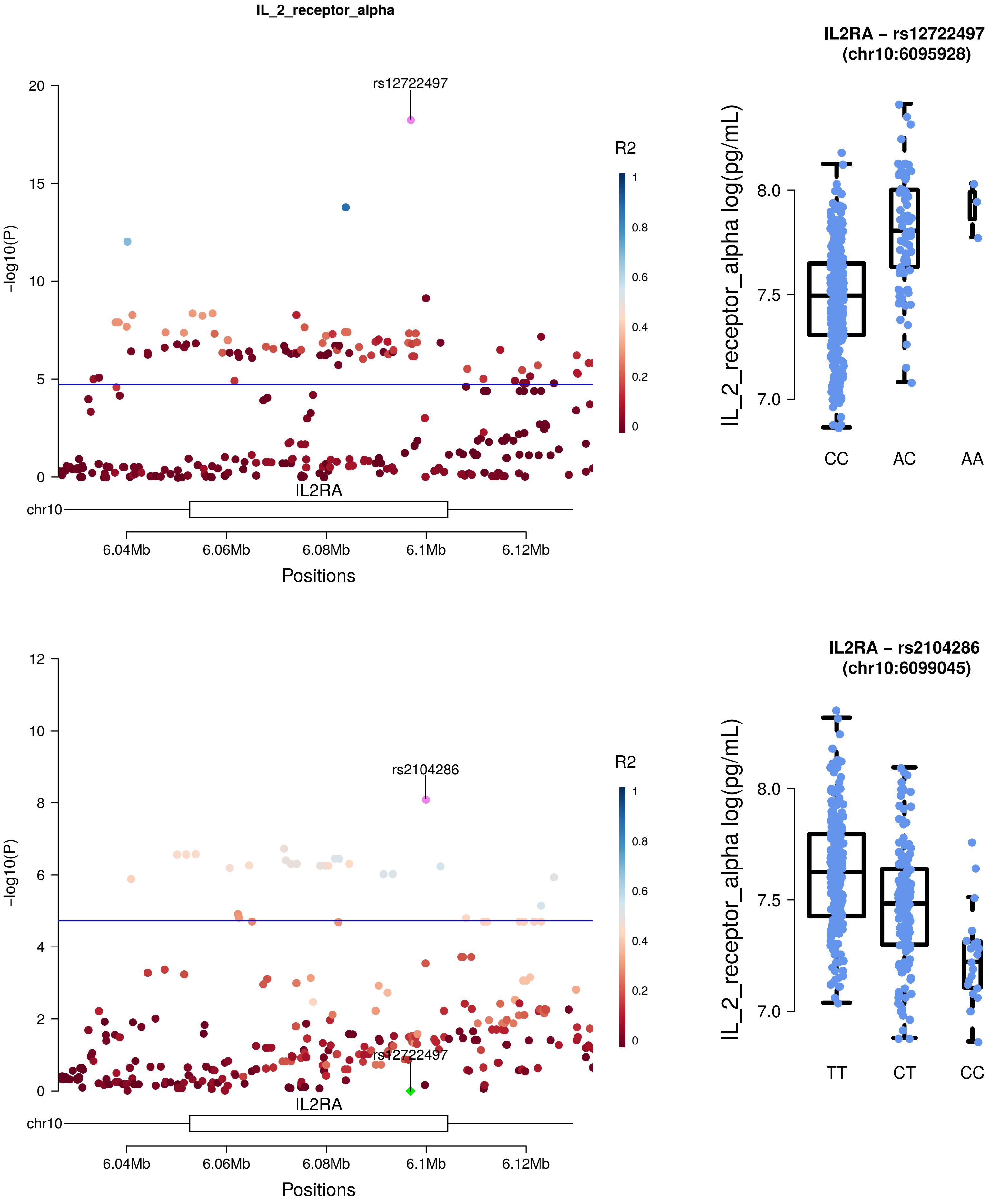

IL_2_receptor_alpha
rs12722497
-log10(P)
R2
IL2RA
chr10
6.04Mb
6.06Mb
6.08Mb
6.1Mb
6.12Mb
Positions
IL2RA – rs12722497
(chr10:6095928)
IL_2_receptor_alpha log(pg/mL)
CC
AC
AA
rs2104286
rs12722497
IL2RA – rs2104286
(chr10:6099045)
TT
CT
CC

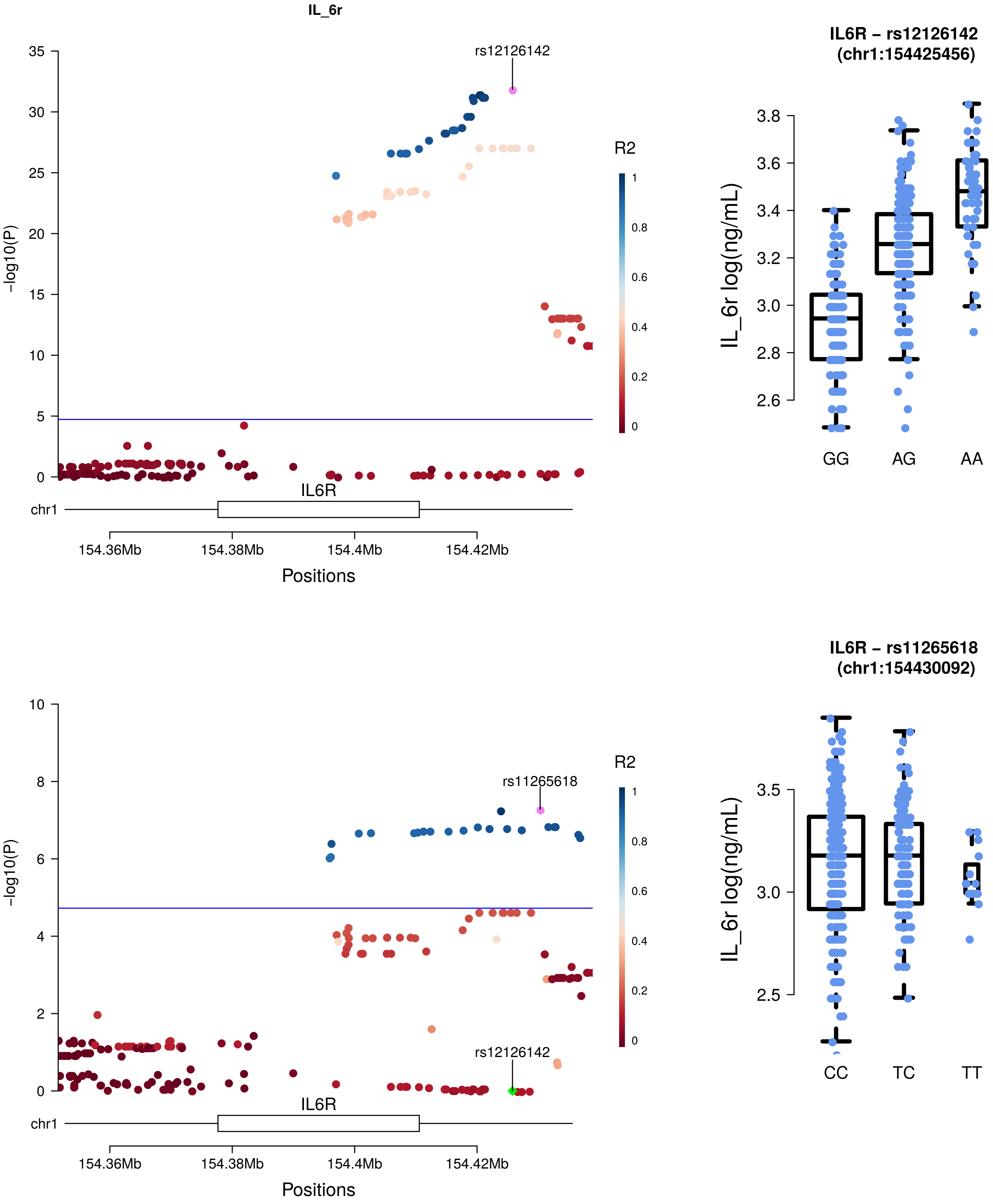
IL_6r
rs12126142
R2
-log10(P)
IL6R
chr1
154.36Mb
154.38Mb
154.4Mb
154.42Mb
Positions
IL6R – rs12126142
(chr1:154425456)
IL_6r log(ng/mL)
GG
AG
AA
rs11265618
rs12126142
IL6R – rs11265618
(chr1:154430092)
CC
TC
TT

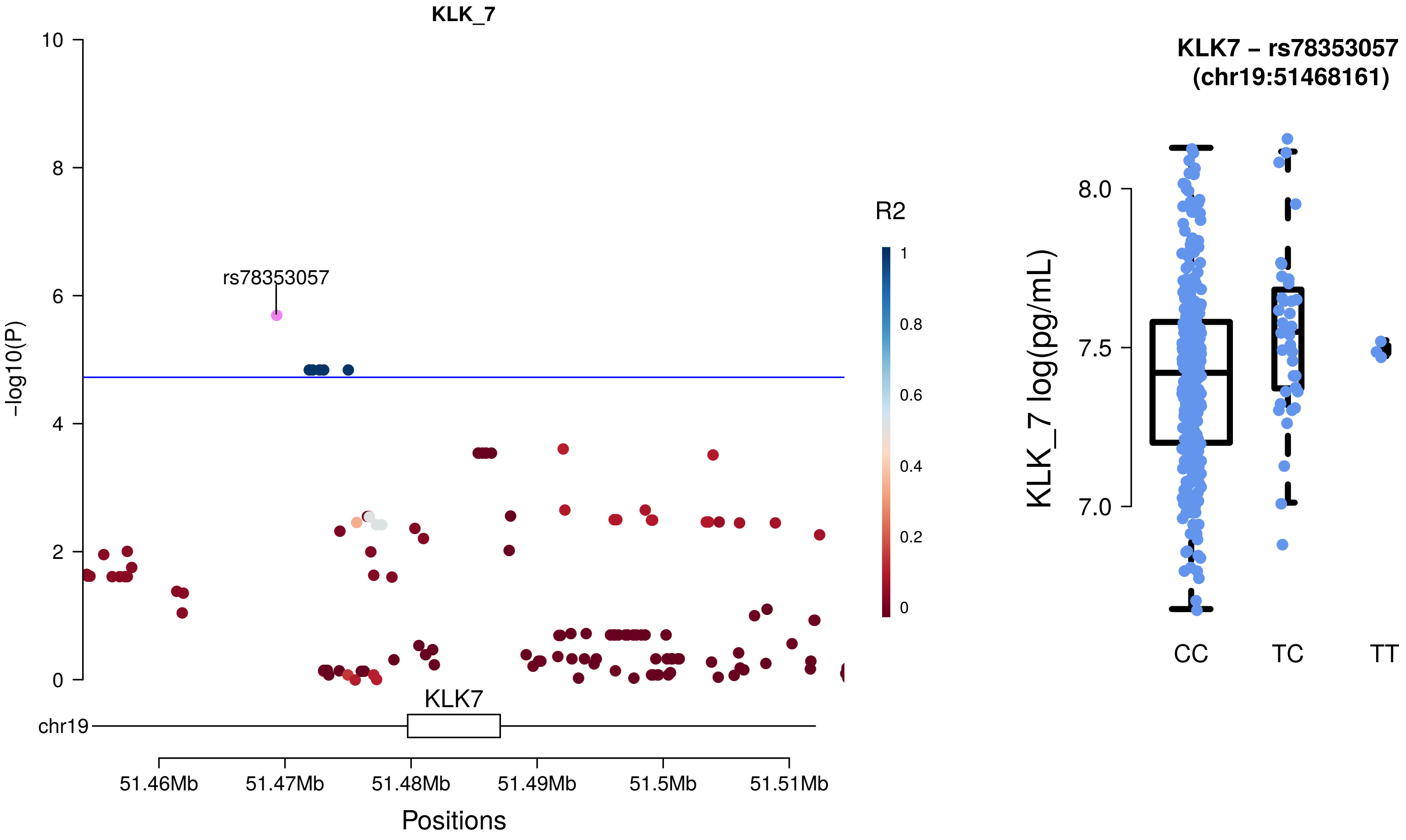

KLK_7
rs78353057
-log10(P)
0
2
4
6
8
10
R2
1
0.8
0.6
0.4
0.2
0
KLK7
chr19
51.46Mb
51.47Mb
51.48Mb
51.49Mb
51.5Mb
51.51Mb
Positions
KLK7 – rs78353057
(chr19:51468161)
KLK_7 log(pg/mL)
8.0
7.5
7.0
CC
TC
TT

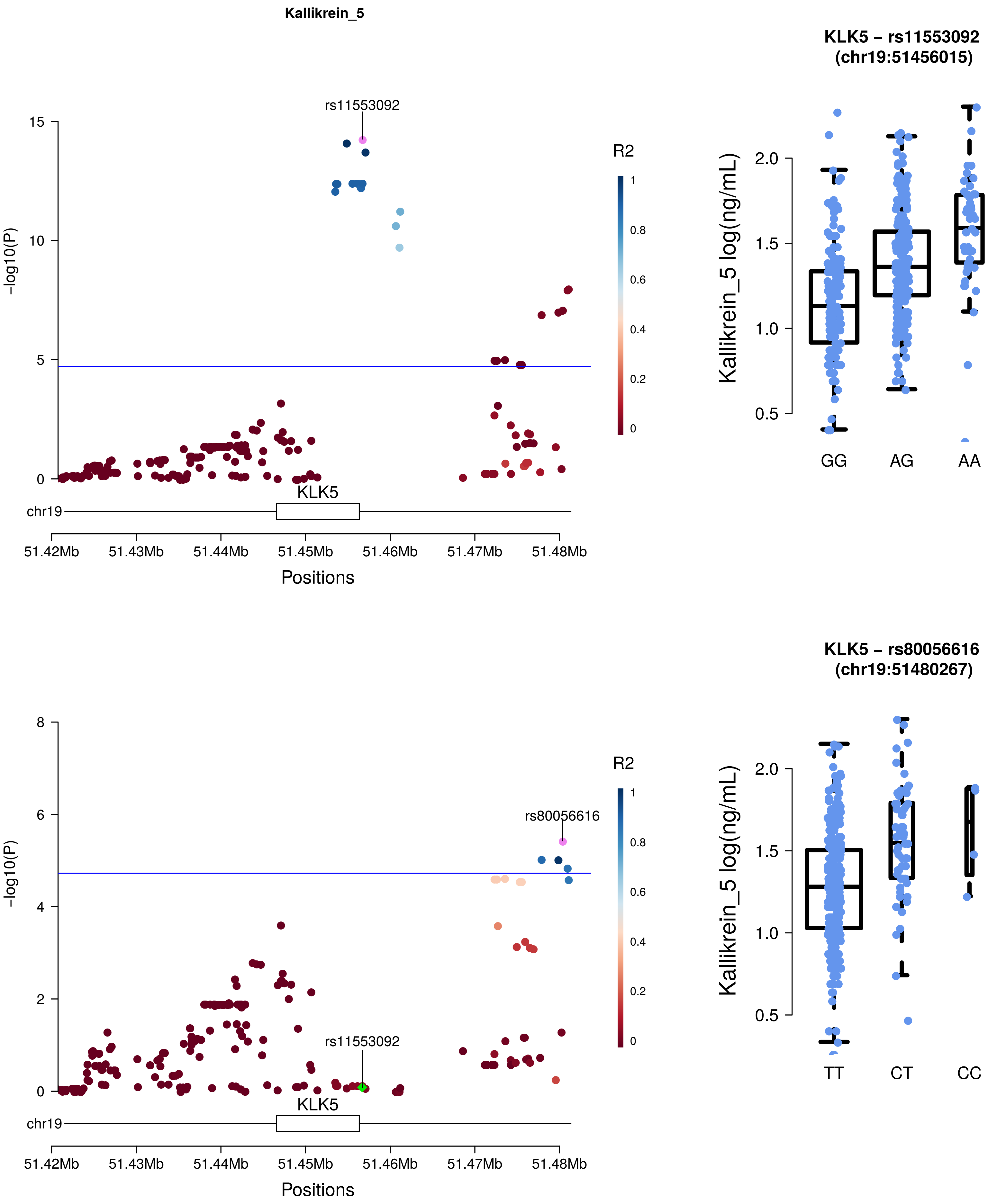

Kallikrein_5
rs11553092
-log10(P)
R2
KLK5
chr19
51.42Mb
51.43Mb
51.44Mb
51.45Mb
51.46Mb
51.47Mb
51.48Mb
Positions
KLK5 – rs11553092 (chr19:51456015)
Kallikrein_5 log(ng/mL)
GG
AG
AA
rs80056616
rs11553092
KLK5 – rs80056616 (chr19:51480267)
TT
CT
CC

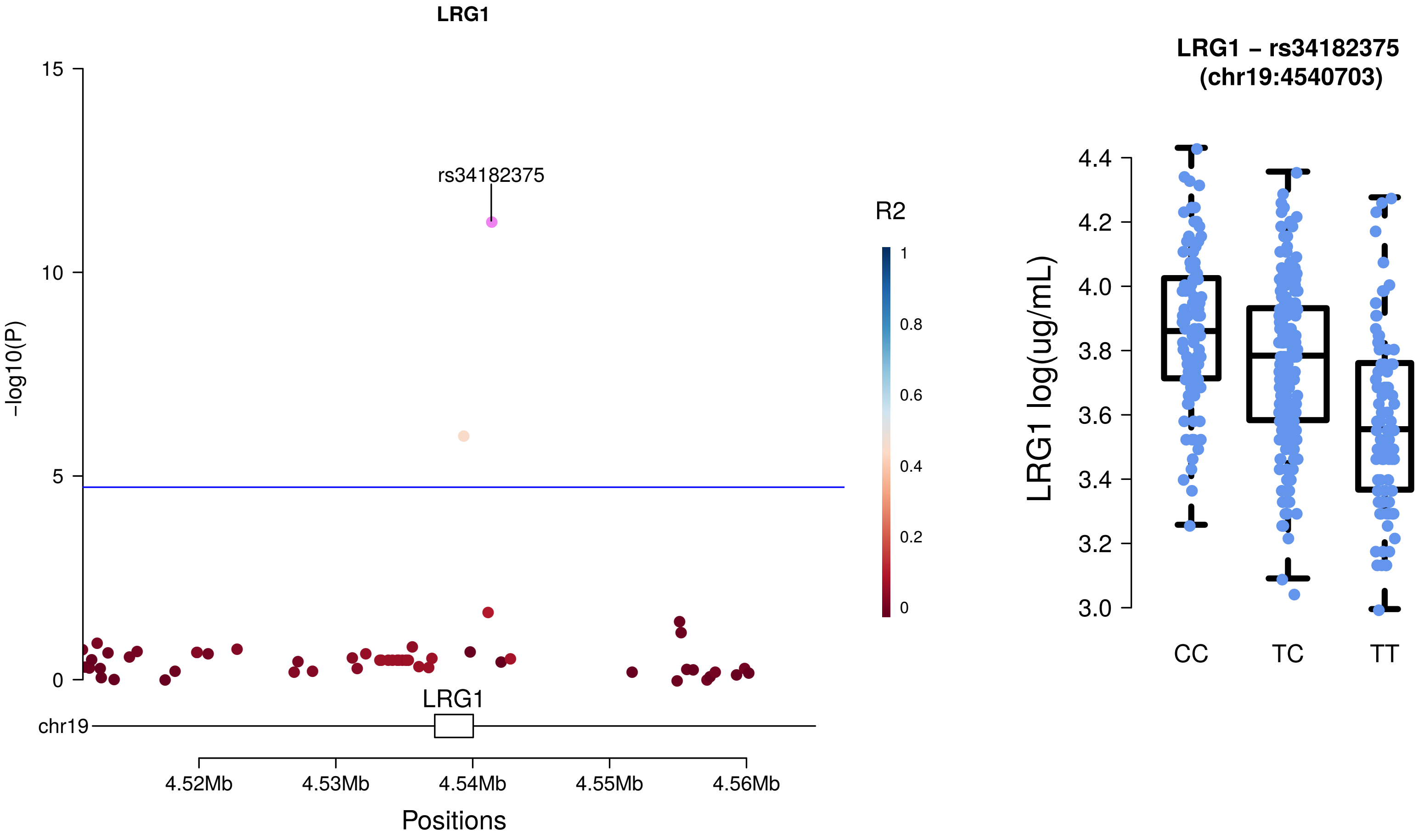
LRG1
15
10
5
0
-log10(P)
rs34182375
R2
1
0.8
0.6
0.4
0.2
0
LRG1
chr19
4.52Mb
4.53Mb
4.54Mb
4.55Mb
4.56Mb
Positions
LRG1 – rs34182375
(chr19:4540703)
LRG1 log(ug/mL)
4.4
4.2
4.0
3.8
3.6
3.4
3.2
3.0
CC
TC
TT

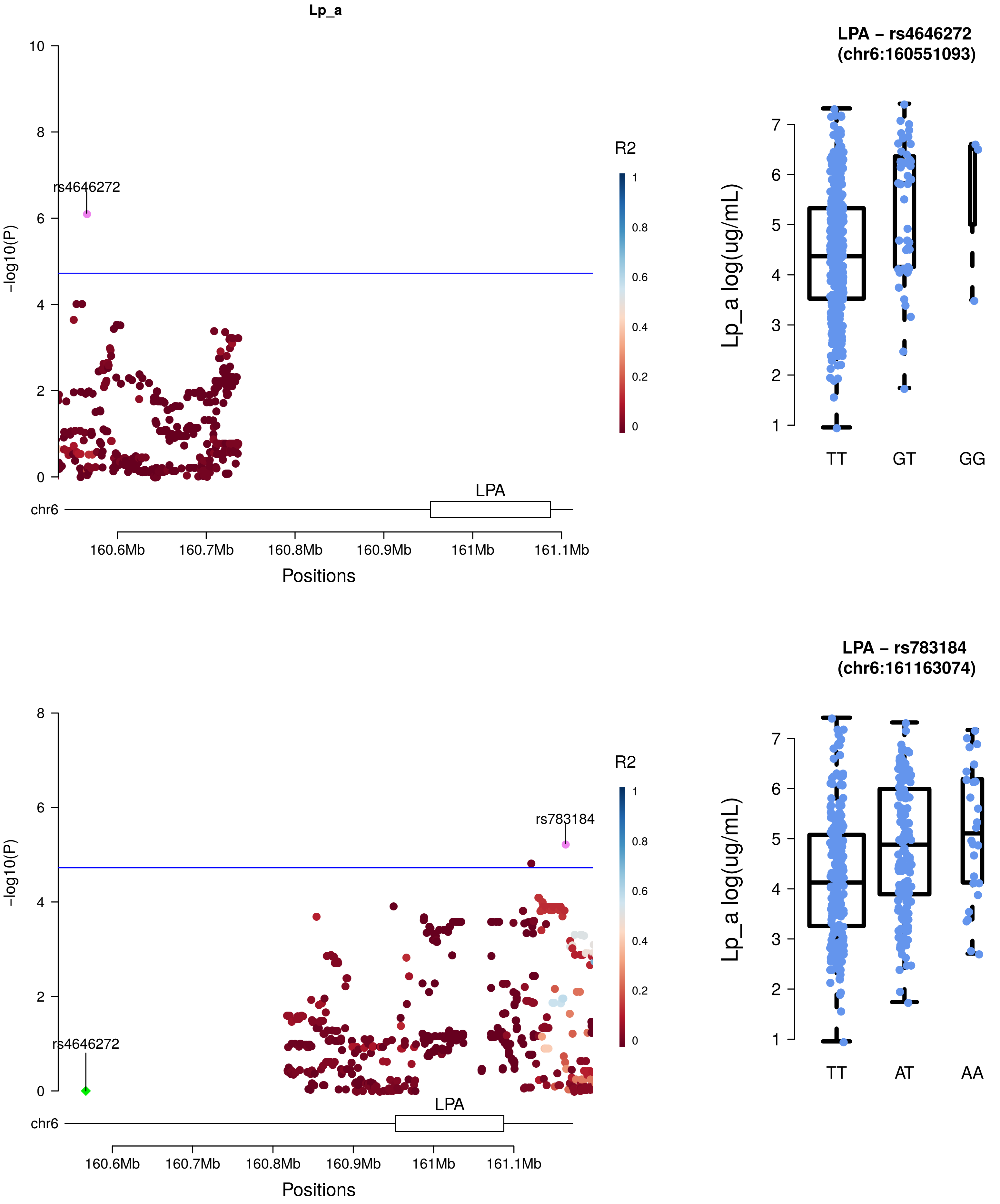

Lp_a
-log10(P)
rs4646272
R2
chr6
LPA
160.6Mb
160.7Mb
160.8Mb
160.9Mb
161Mb
161.1Mb
Positions
LPA – rs4646272
(chr6:160551093)
Lp_a log(ug/mL)
TT
GT
GG
rs783184
LPA – rs783184
(chr6:161163074)
TT
AT
AA

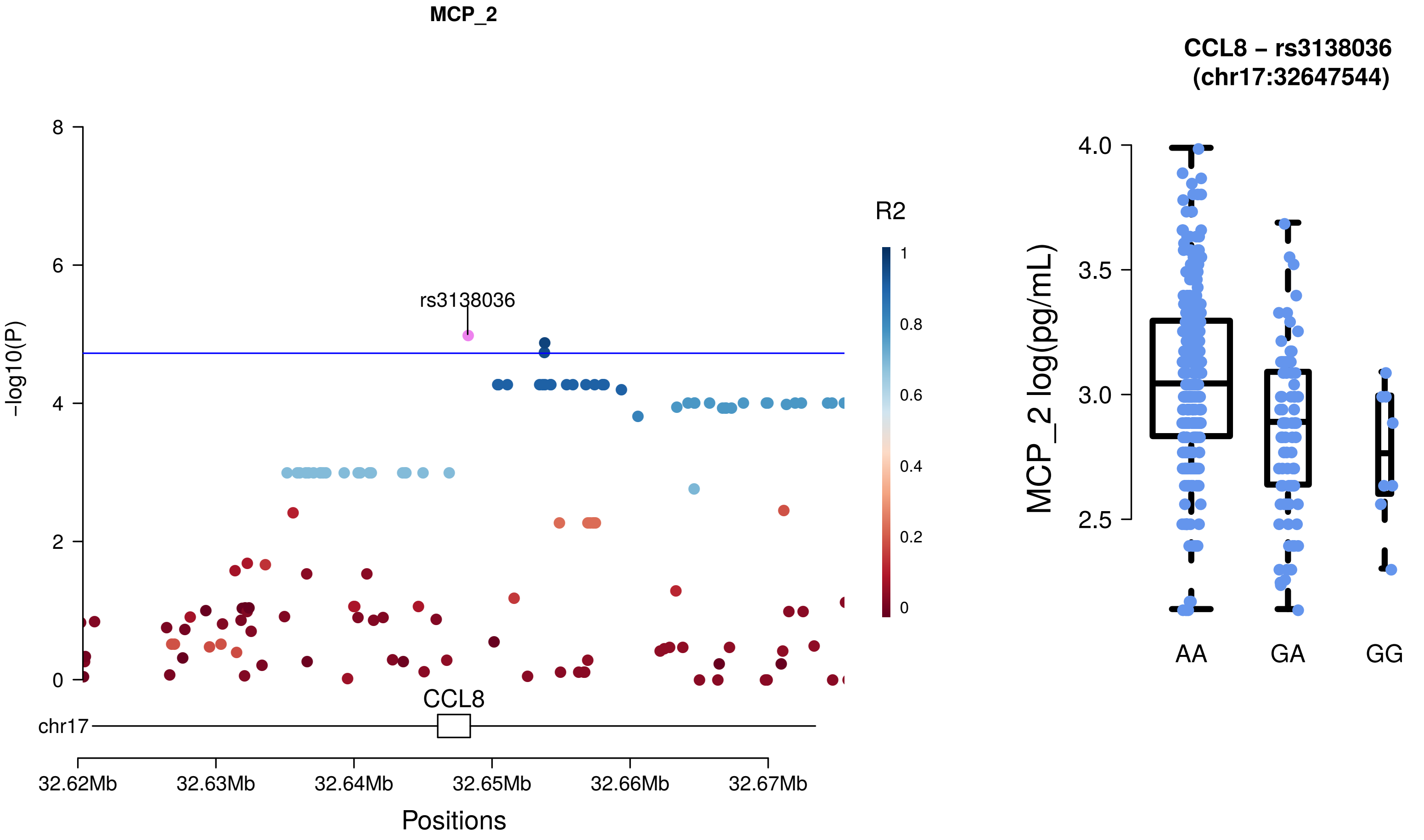
MCP_2
rs3138036
R2
1
0.8
0.6
0.4
0.2
0
8
6
4
2
0
−log10(P)
CCL8
chr17
32.62Mb
32.63Mb
32.64Mb
32.65Mb
32.66Mb
32.67Mb
Positions
CCL8 − rs3138036
(chr17:32647544)
4.0
3.5
3.0
2.5
MCP_2 log(pg/mL)
AA
GA
GG

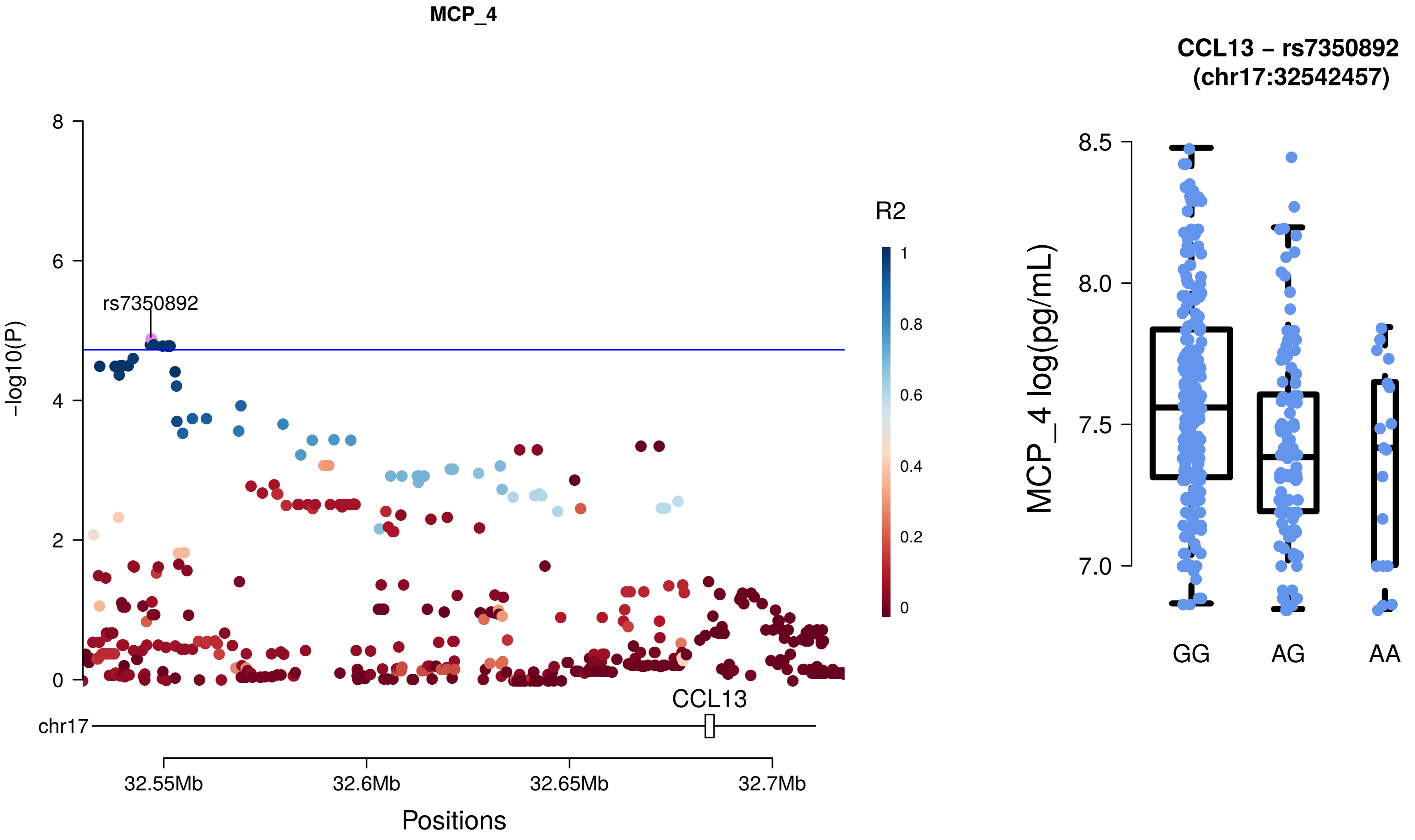

MCP_4
rs7350892
−log10(P)
8
6
4
2
0
R2
1
0.8
0.6
0.4
0.2
0
CCL13
chr17
32.55Mb
32.6Mb
32.65Mb
32.7Mb
Positions
CCL13 − rs7350892
(chr17:32542457)
MCP_4 log(pg/mL)
8.5
8.0
7.5
7.0
GG
AG
AA

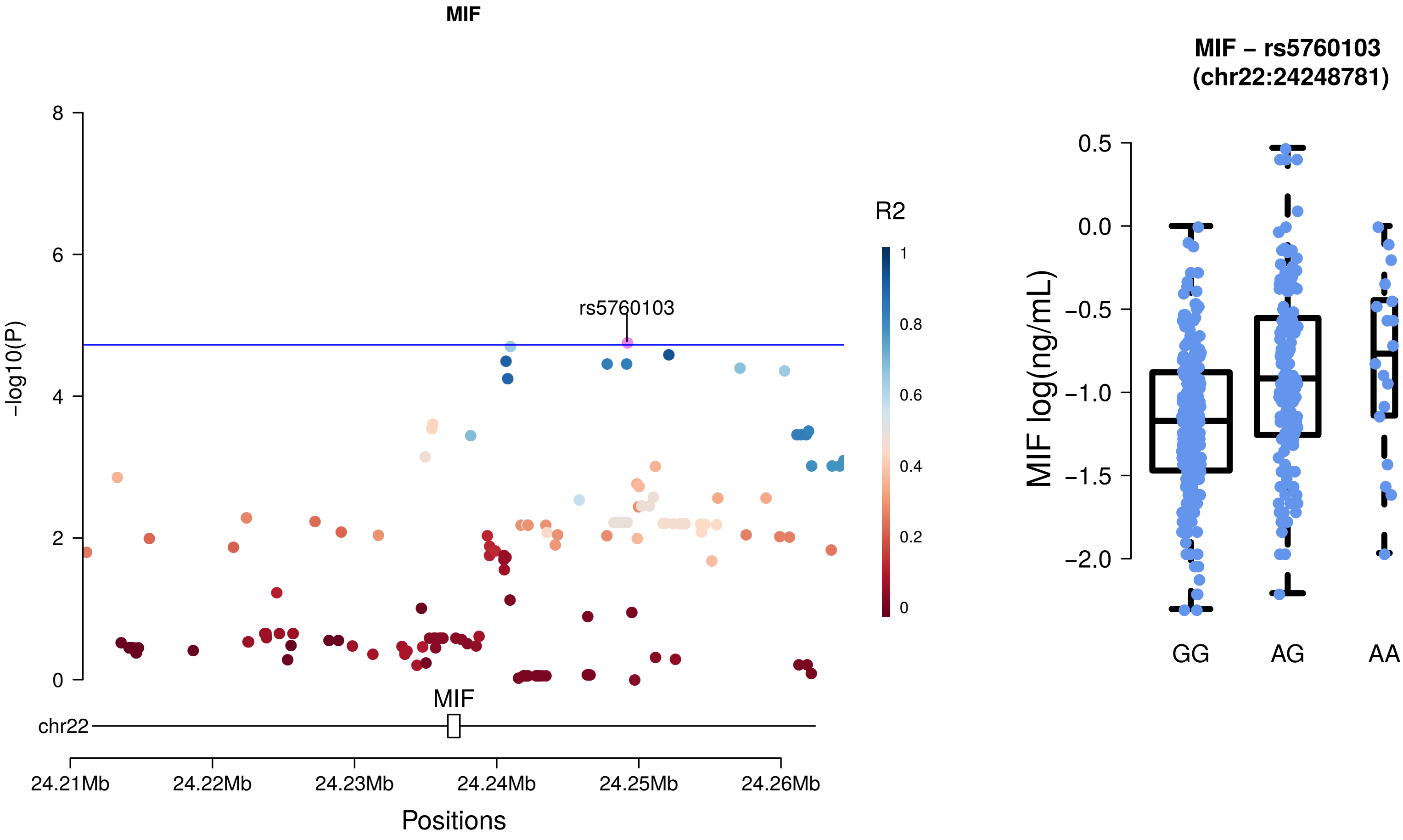

MIF
MIF − rs5760103
(chr22:24248781)
R2
1
0.8
0.6
0.4
0.2
0
−log10(P)
8
6
4
2
0
rs5760103
MIF
chr22
24.21Mb
24.22Mb
24.23Mb
24.24Mb
24.25Mb
24.26Mb
Positions
MIF log(ng/mL)
0.5
0.0
−0.5
−1.0
−1.5
−2.0
GG
AG
AA

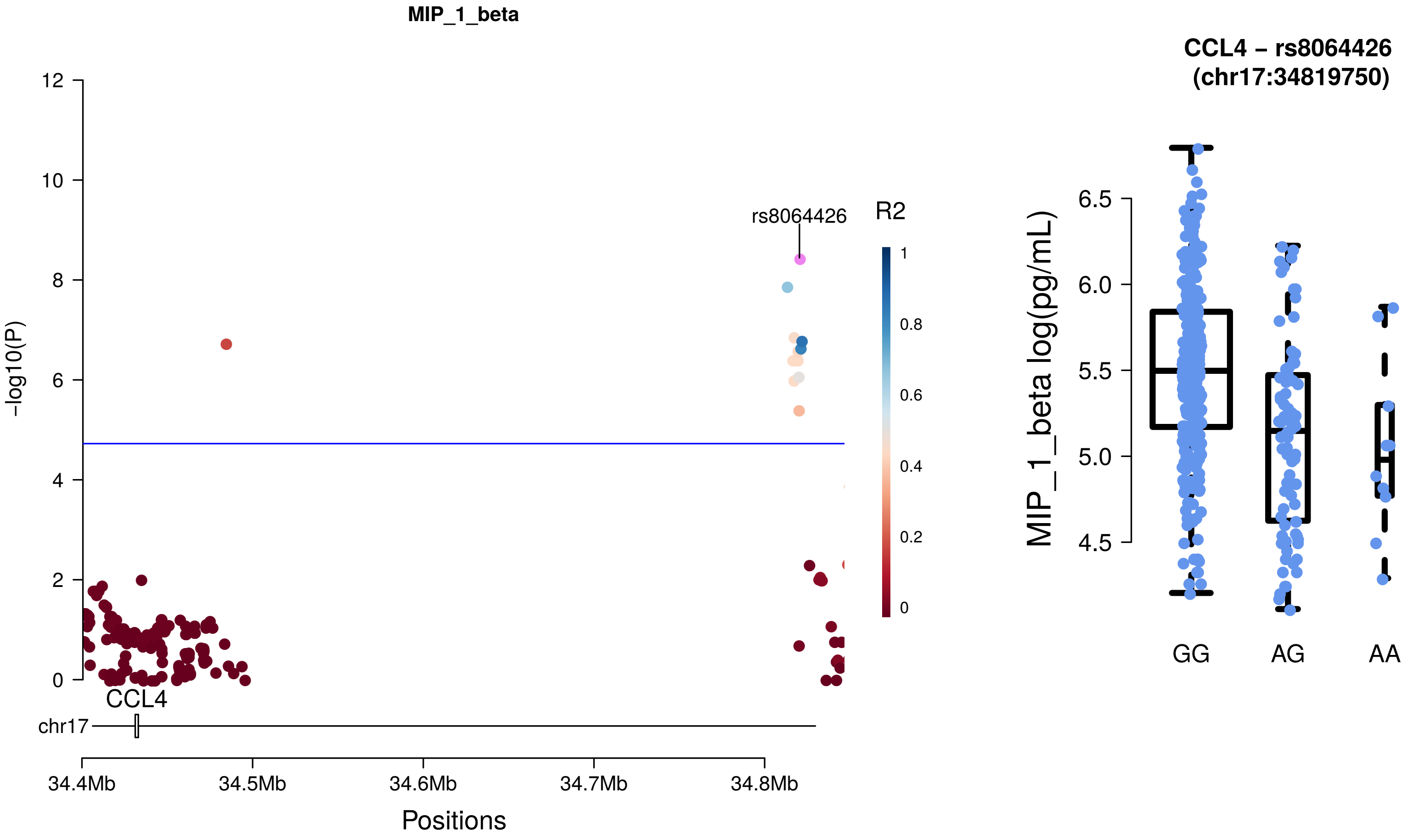
MIP_1_beta
-log10(P)
12
10
8
6
4
2
0
rs8064426
R2
1
0.8
0.6
0.4
0.2
0
CCL4
chr17
34.4Mb
34.5Mb
34.6Mb
34.7Mb
34.8Mb
Positions
CCL4 – rs8064426
(chr17:34819750)
MIP_1_beta log(pg/mL)
6.5
6.0
5.5
5.0
4.5
GG
AG
AA

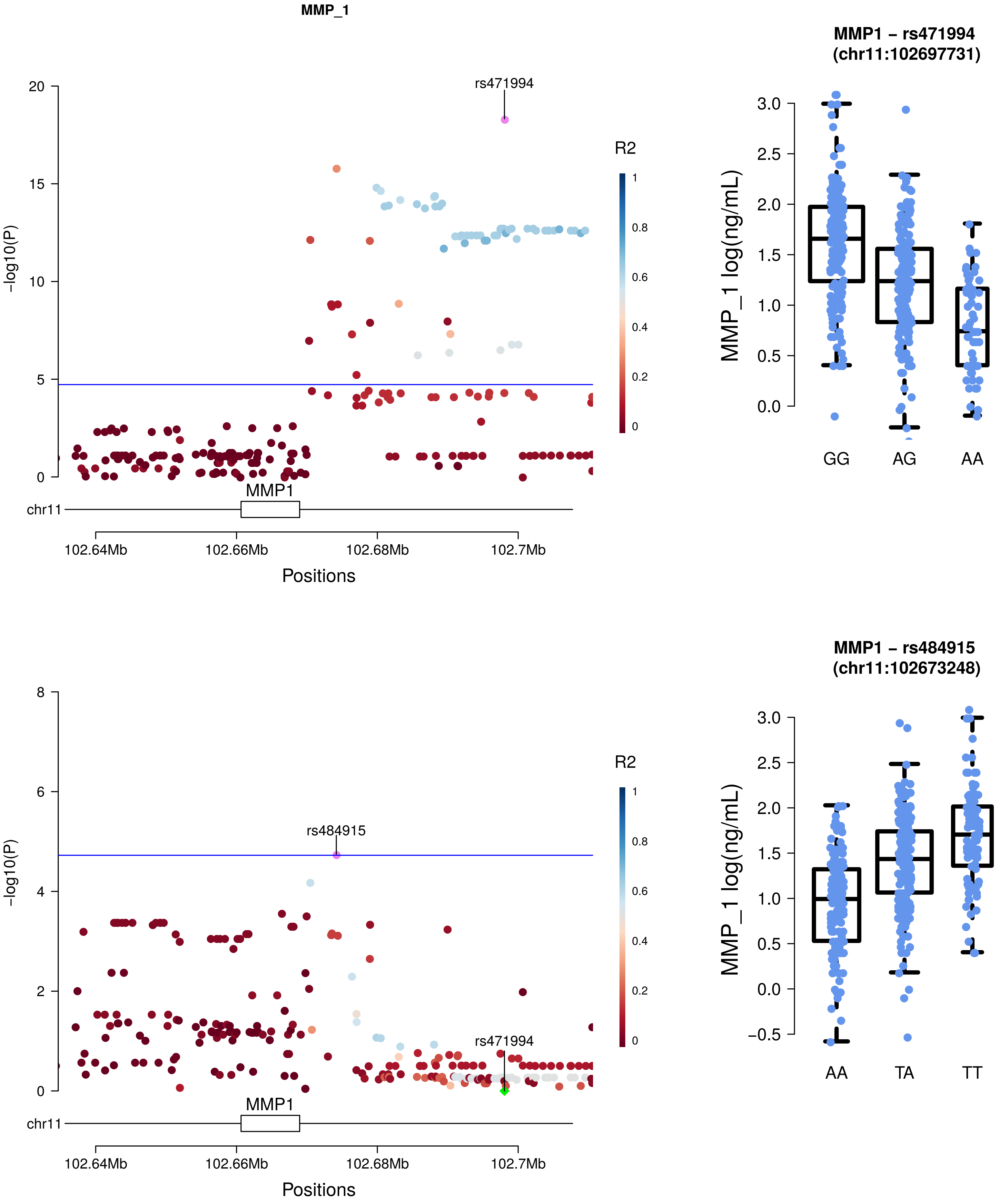
MMP_1
rs471994
-log10(P)
R2
1
0.8
0.6
0.4
0.2
0
MMP1
chr11
102.64Mb
102.66Mb
102.68Mb
102.7Mb
Positions
MMP1 – rs471994
(chr11:102697731)
MMP_1 log(ng/mL)
GG
AG
AA
rs484915
MMP1 – rs484915
(chr11:102673248)
AA
TA
TT

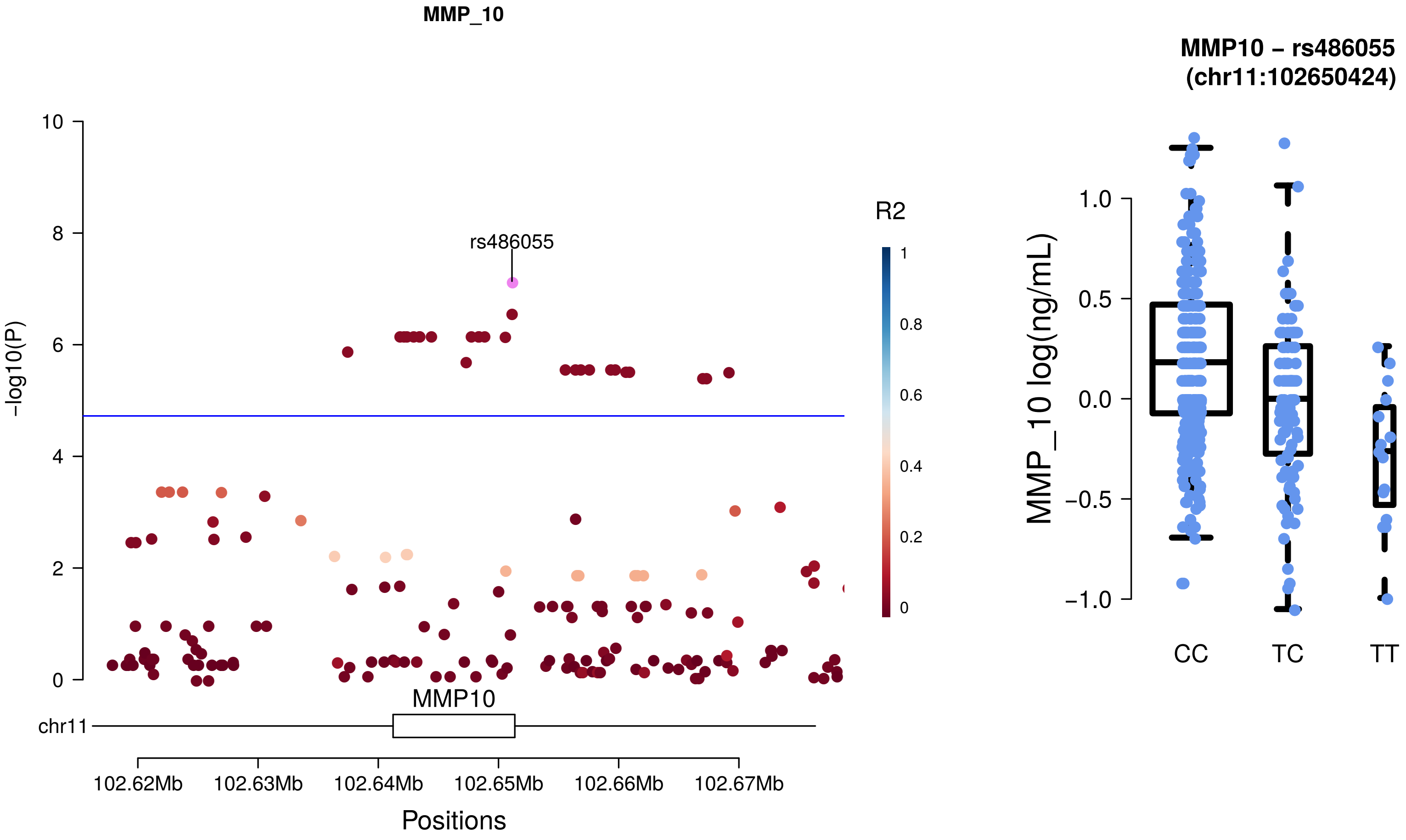

MMP_10
-log10(P)
10
8
6
4
2
0
rs486055
R2
1
0.8
0.6
0.4
0.2
0
MMP10
chr11
102.62Mb
102.63Mb
102.64Mb
102.65Mb
102.66Mb
102.67Mb
Positions
MMP10 – rs486055
(chr11:102650424)
MMP_10 log(ng/mL)
1.0
0.5
0.0
−0.5
−1.0
CC
TC
TT

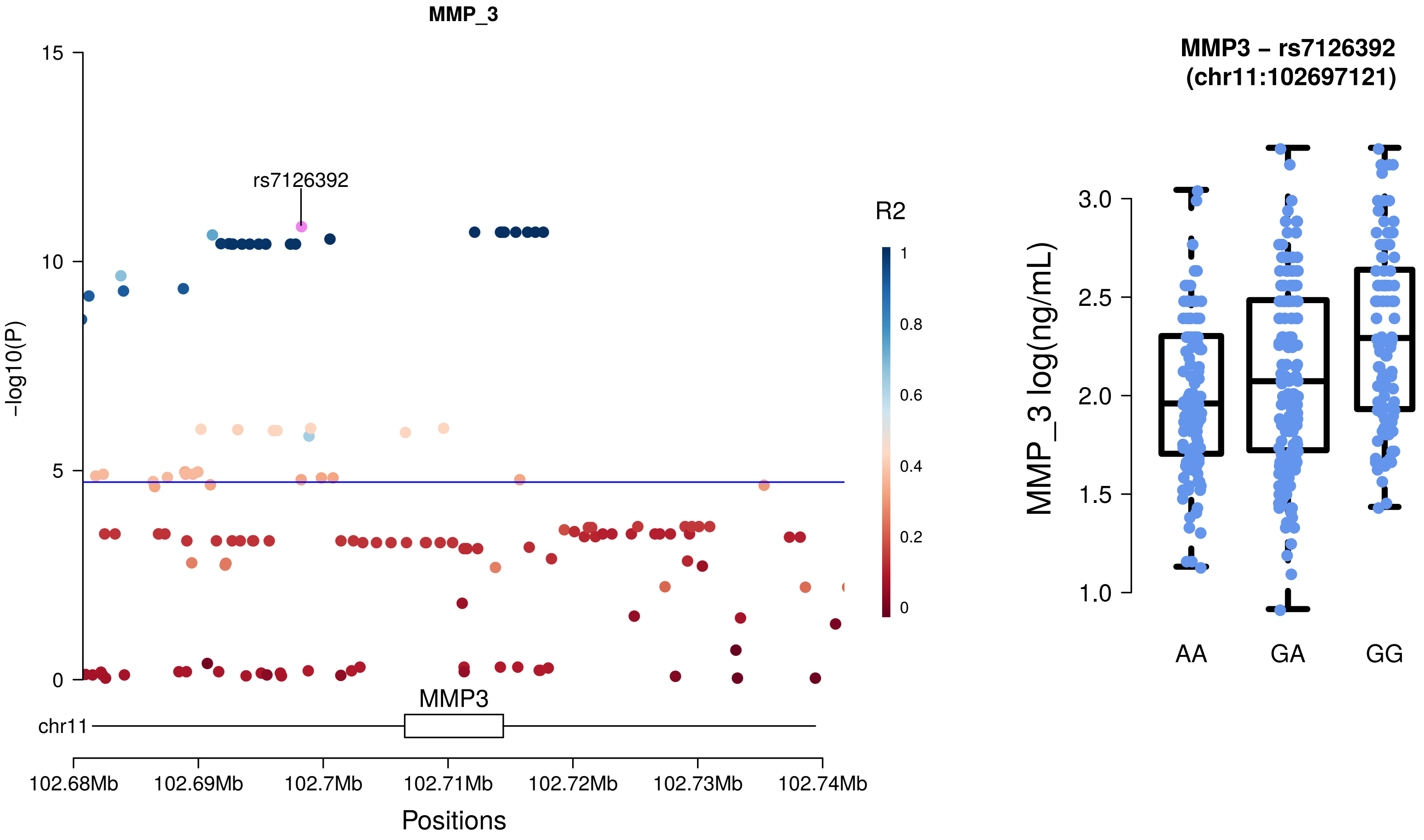

MMP_3
rs7126392
R2
1
0.8
0.6
0.4
0.2
0
15
10
5
0
-log10(P)
MMP3
chr11
102.68Mb
102.69Mb
102.7Mb
102.71Mb
102.72Mb
102.73Mb
102.74Mb
Positions
MMP3 – rs7126392
(chr11:102697121)
MMP_3 log(ng/mL)
3.0
2.5
2.0
1.5
1.0
AA
GA
GG

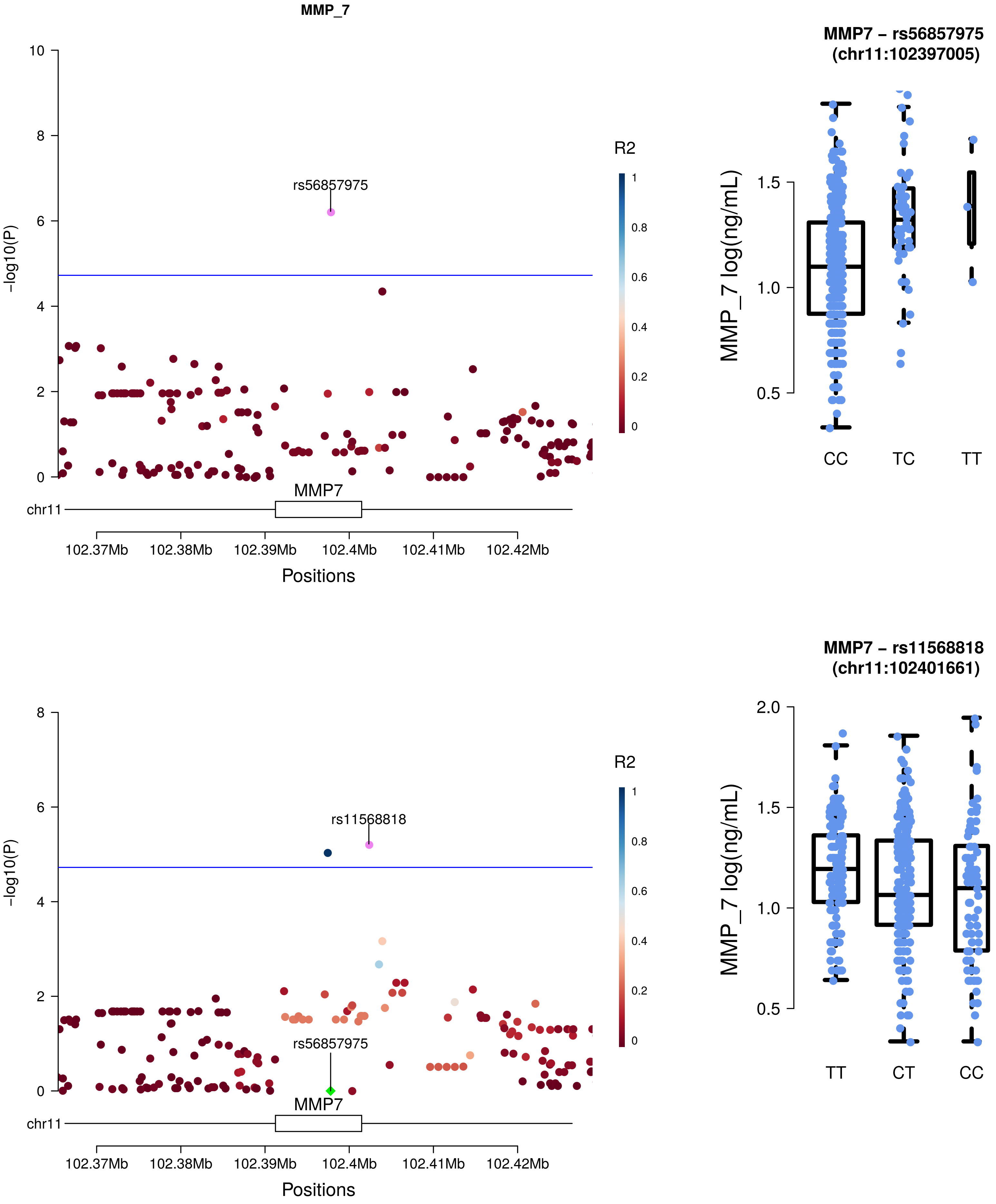
MMP_7
rs56857975
-log10(P)
R2
MMP7
chr11
102.37Mb
102.38Mb
102.39Mb
102.4Mb
102.41Mb
102.42Mb
Positions
MMP7 – rs56857975
(chr11:102397005)
MMP_7 log(ng/mL)
CC
TC
TT
rs11568818
rs56857975
MMP7 – rs11568818
(chr11:102401661)
TT
CT
CC

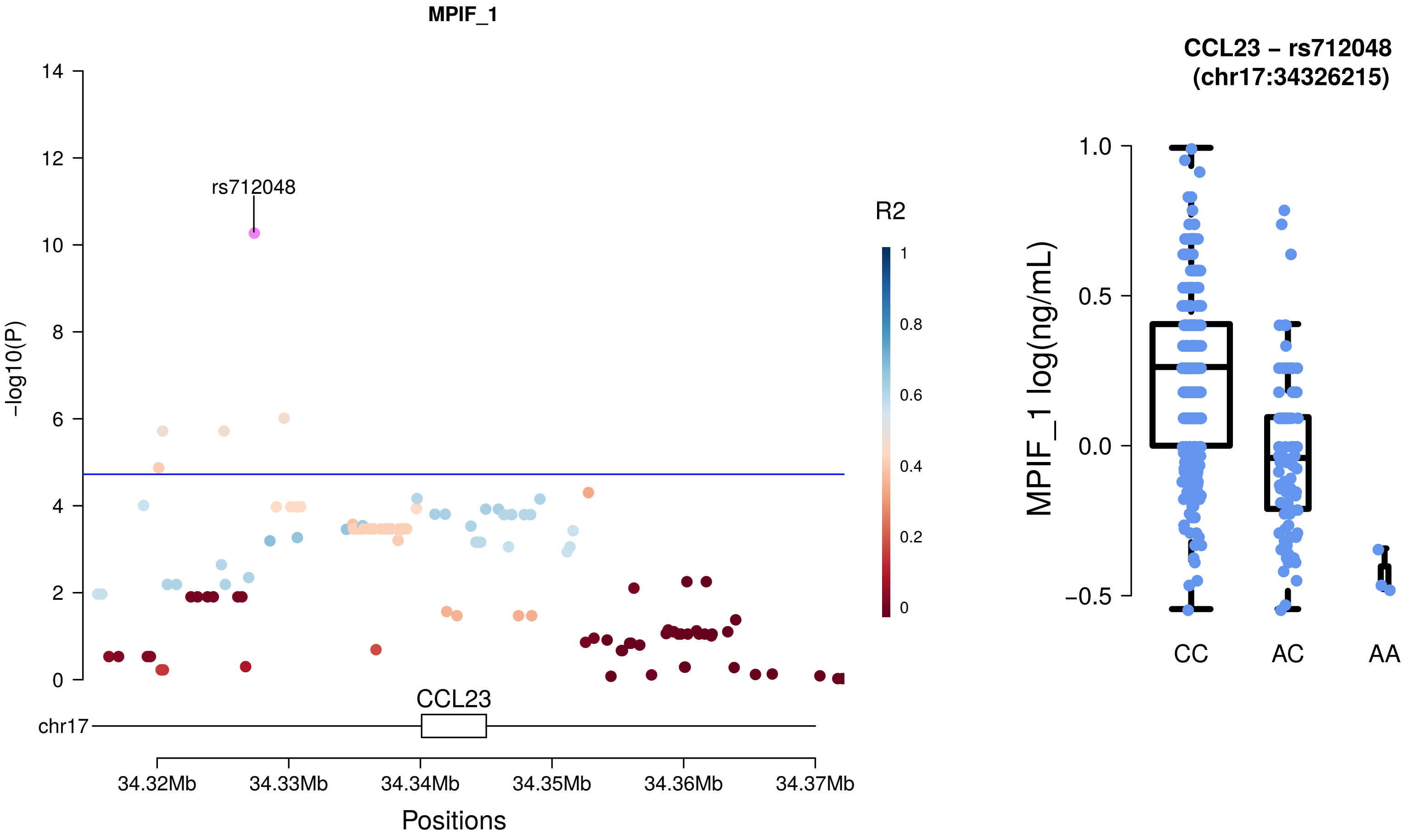

MPIF_1
rs712048
-log10(P)
R2
CCL23
chr17
34.32Mb
34.33Mb
34.34Mb
34.35Mb
34.36Mb
34.37Mb
Positions
CCL23 – rs712048
(chr17:34326215)
MPIF_1 log(ng/mL)
CC
AC
AA

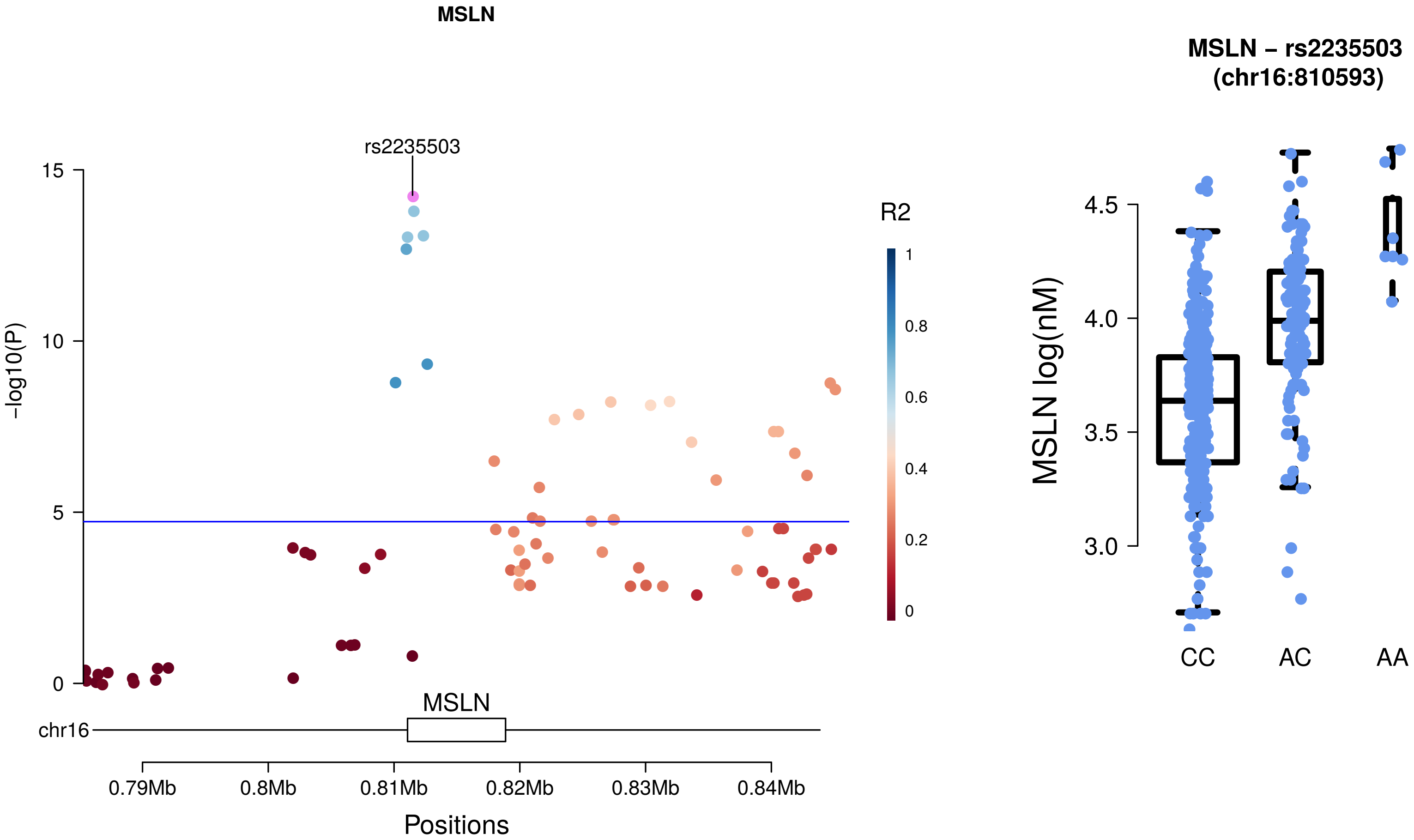

MSLN
rs2235503
-log10(P)
15
10
5
0
R2
1
0.8
0.6
0.4
0.2
0
MSLN
chr16
0.79Mb
0.8Mb
0.81Mb
0.82Mb
0.83Mb
0.84Mb
Positions
MSLN – rs2235503
(chr16:810593)
MSLN log(nM)
4.5
4.0
3.5
3.0
CC
AC
AA

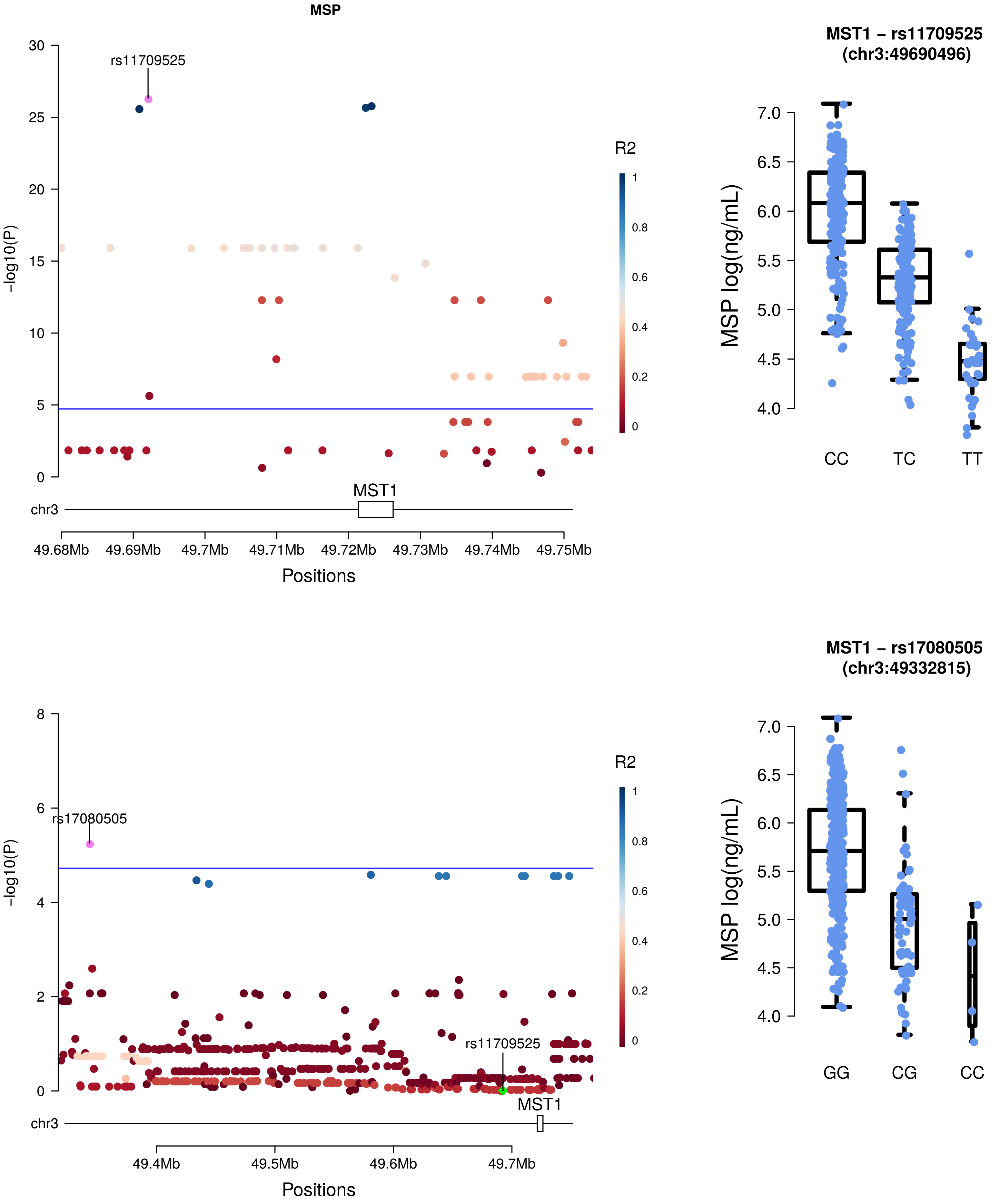
MSP
rs11709525
-log10(P)
R2
MST1
chr3
49.68Mb
49.69Mb
49.7Mb
49.71Mb
49.72Mb
49.73Mb
49.74Mb
49.75Mb
Positions
MST1 – rs11709525
(chr3:49690496)
MSP log(ng/mL)
CC
TC
TT
rs17080505
rs11709525
49.4Mb
49.5Mb
49.6Mb
49.7Mb
MST1 – rs17080505
(chr3:49332815)
GG
CG
CC

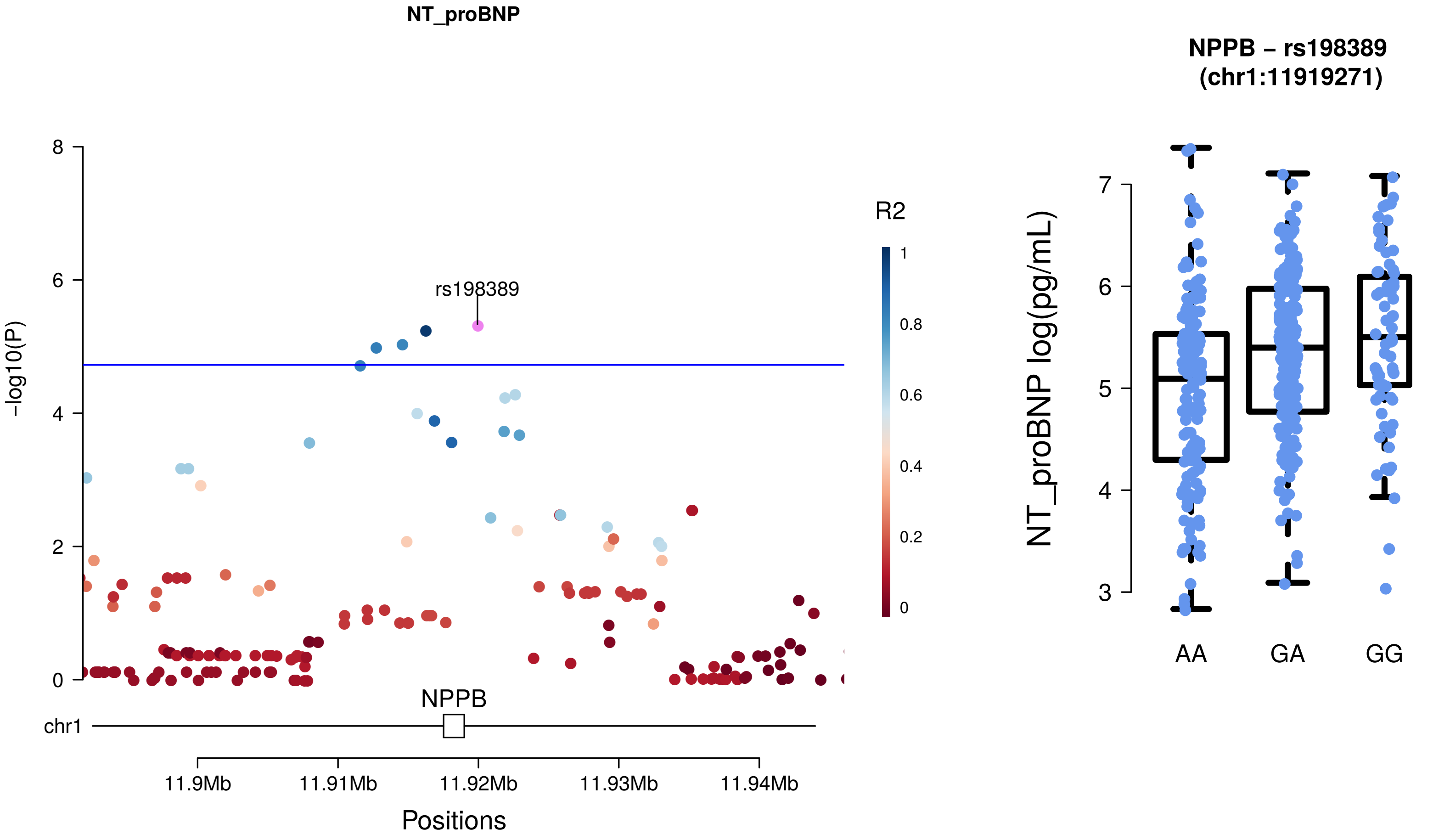

NT_proBNP
-log10(P)
rs198389
R2
NPPB
chr1
11.9Mb
11.91Mb
11.92Mb
11.93Mb
11.94Mb
Positions
NPPB – rs198389
(chr1:11919271)
NT_proBNP log(pg/mL)
AA
GA
GG

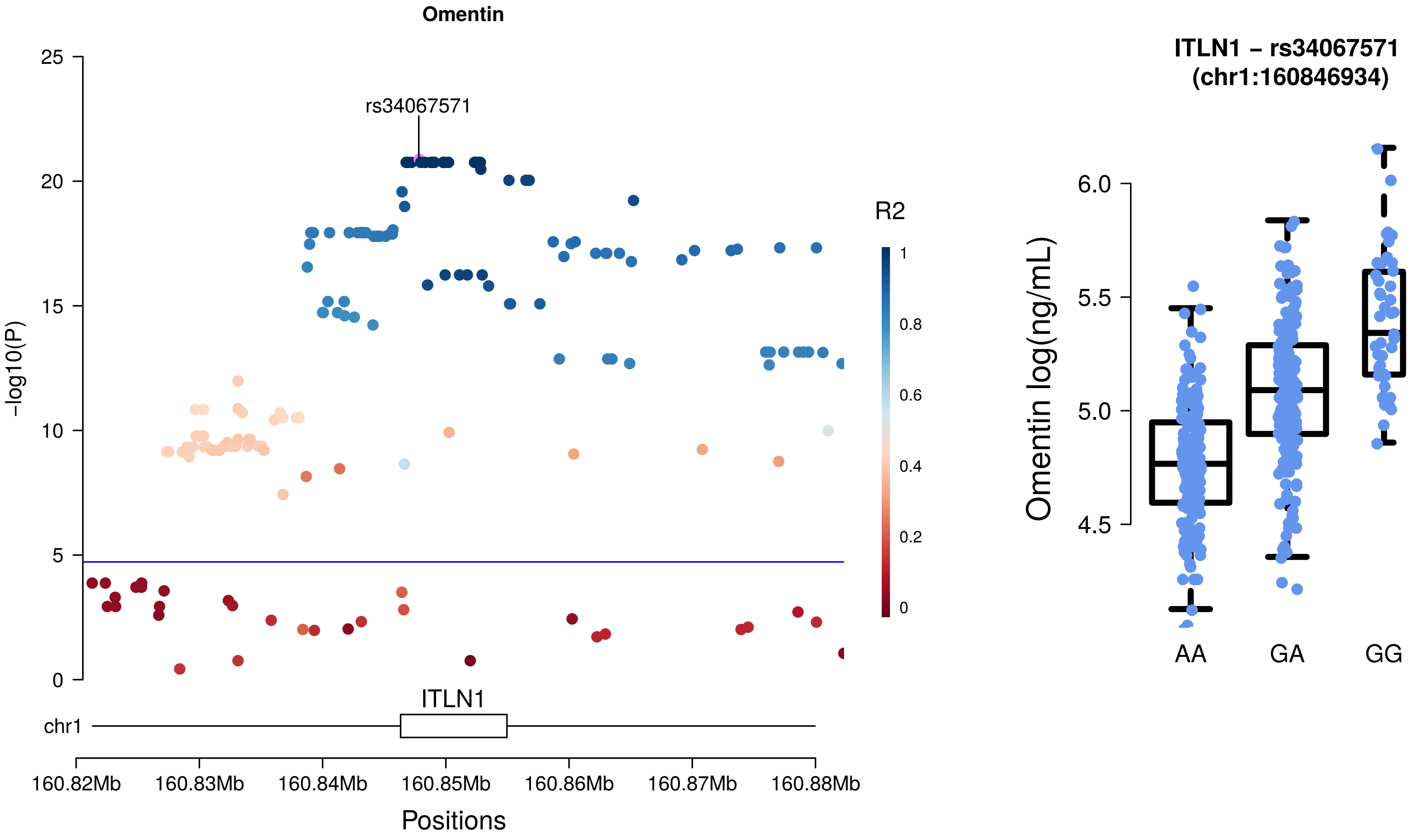
Omentin
rs34067571
-log10(P)
25
20
15
10
5
0
R2
1
0.8
0.6
0.4
0.2
0
ITLN1
chr1
160.82Mb
160.83Mb
160.84Mb
160.85Mb
160.86Mb
160.87Mb
160.88Mb
Positions
ITLN1 – rs34067571
(chr1:160846934)
Omentin log(ng/mL)
6.0
5.5
5.0
4.5
AA
GA
GG

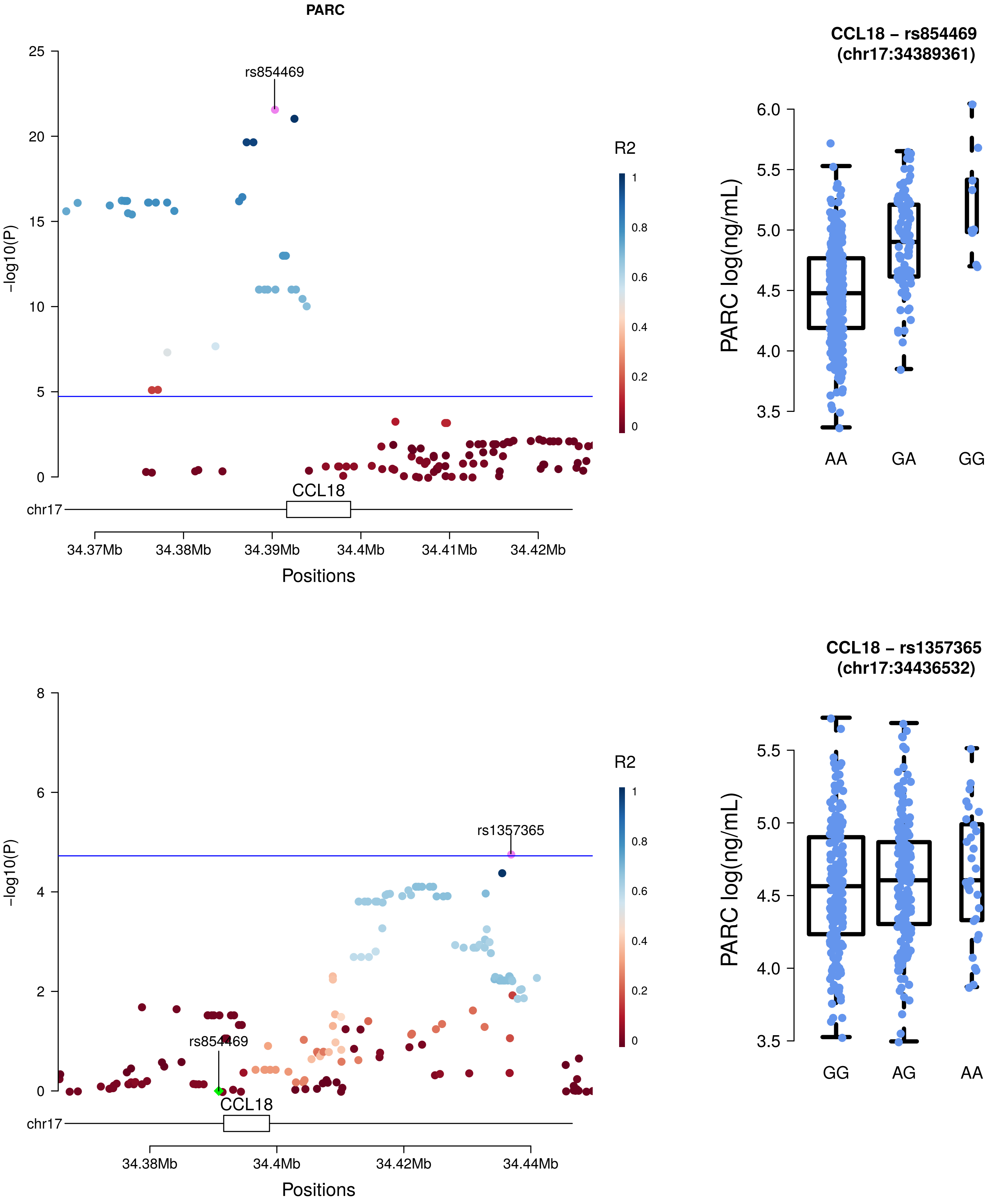

PARC
rs854469
R2
-log10(P)
CCL18
chr17
34.37Mb
34.38Mb
34.39Mb
34.4Mb
34.41Mb
34.42Mb
Positions
CCL18 – rs854469
(chr17:34389361)
PARC log(ng/mL)
AA
GA
GG
rs1357365
rs854469
34.38Mb
34.4Mb
34.42Mb
34.44Mb
CCL18 – rs1357365
(chr17:34436532)
GG
AG
AA

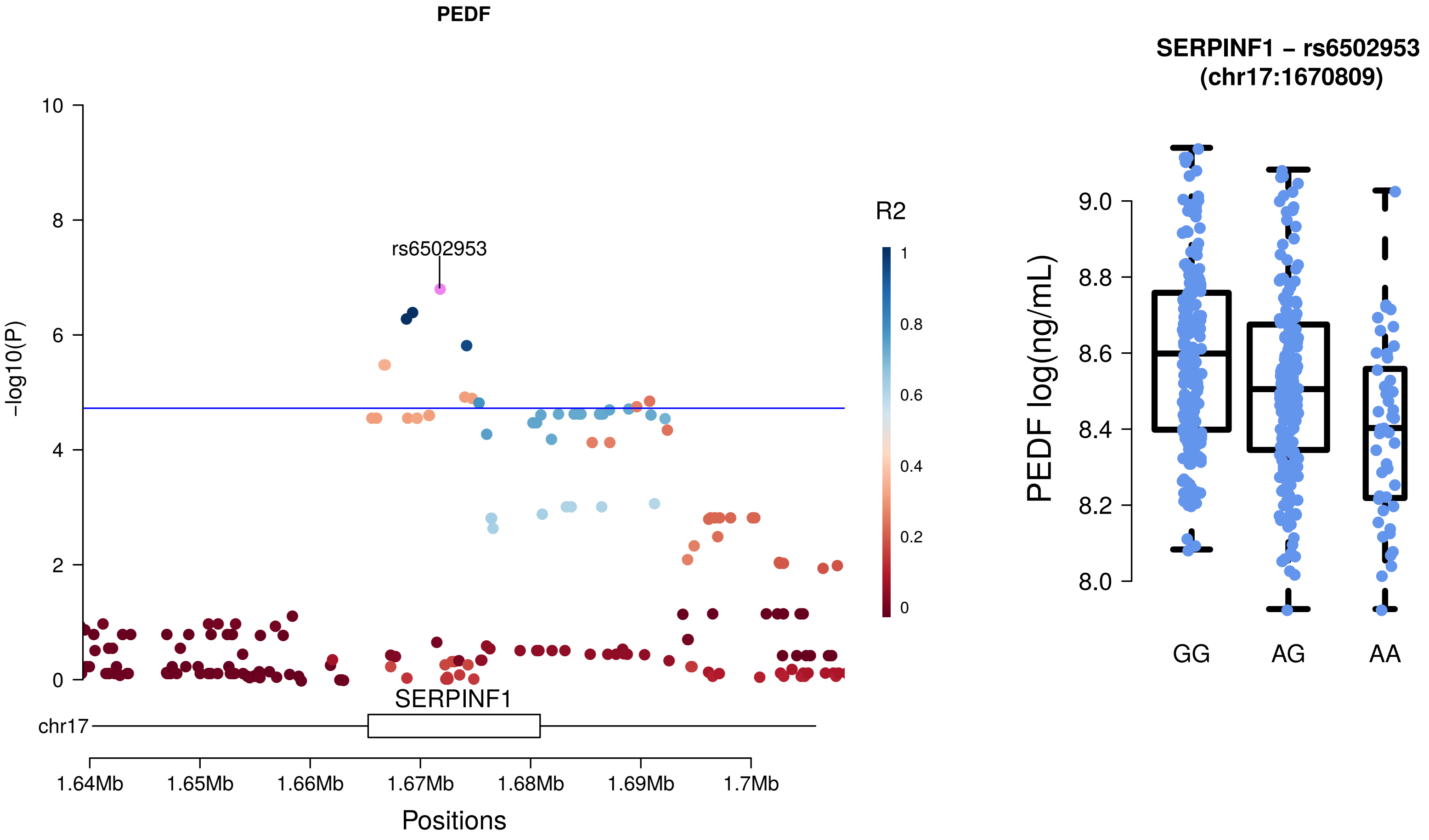
PEDF
-log10(P)
rs6502953
R2
SERPINF1
chr17
1.64Mb
1.65Mb
1.66Mb
1.67Mb
1.68Mb
1.69Mb
1.7Mb
Positions
SERPINF1 – rs6502953
(chr17:1670809)
PEDF log(ng/mL)
GG
AG
AA

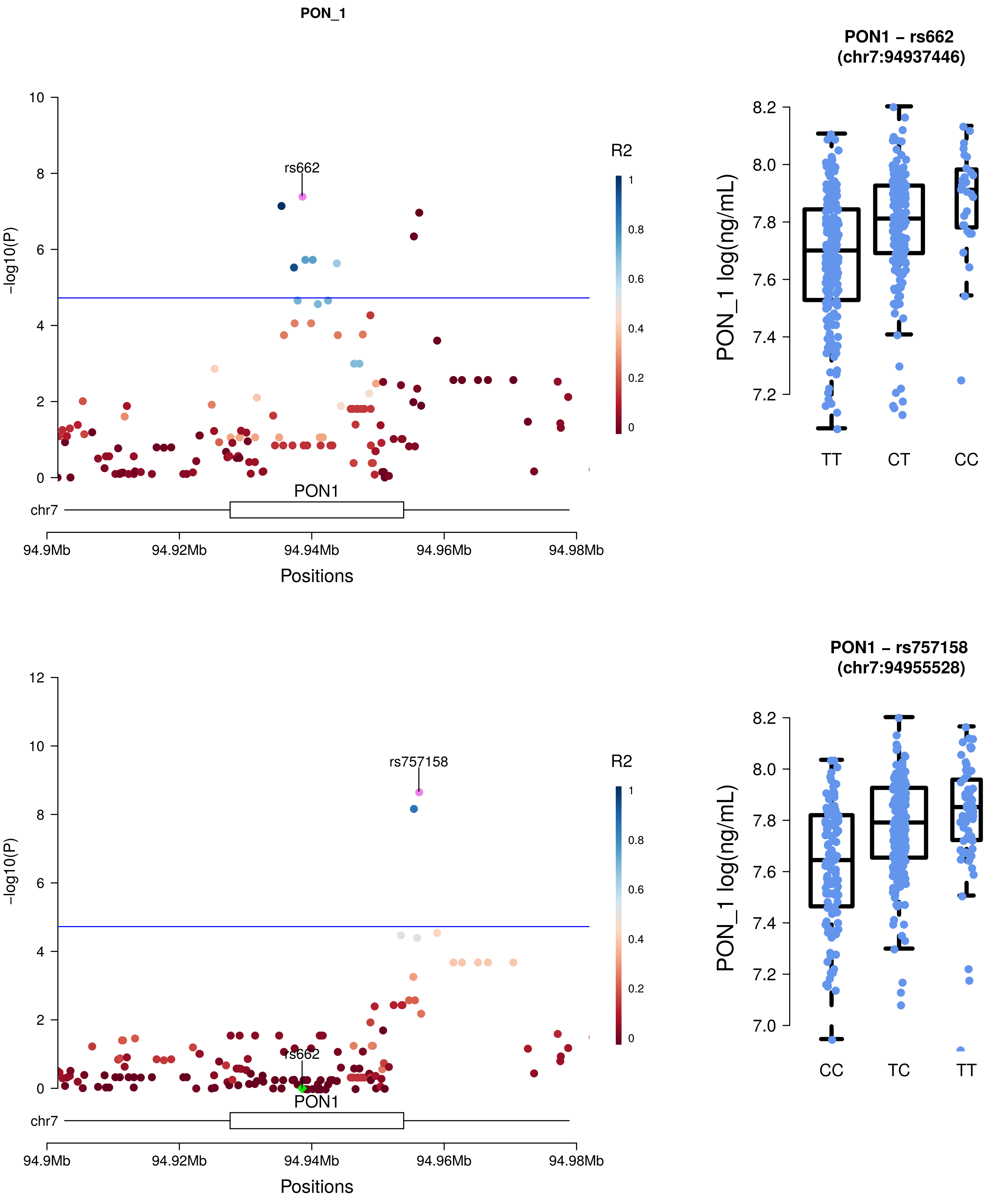

PON_1
rs662
R2
-log10(P)
PON1
chr7
94.9Mb
94.92Mb
94.94Mb
94.96Mb
94.98Mb
Positions
PON1 – rs662 (chr7:94937446)
PON_1 log(ng/mL)
TT
CT
CC
rs757158
PON1 – rs757158 (chr7:94955528)
CC
TC
TT

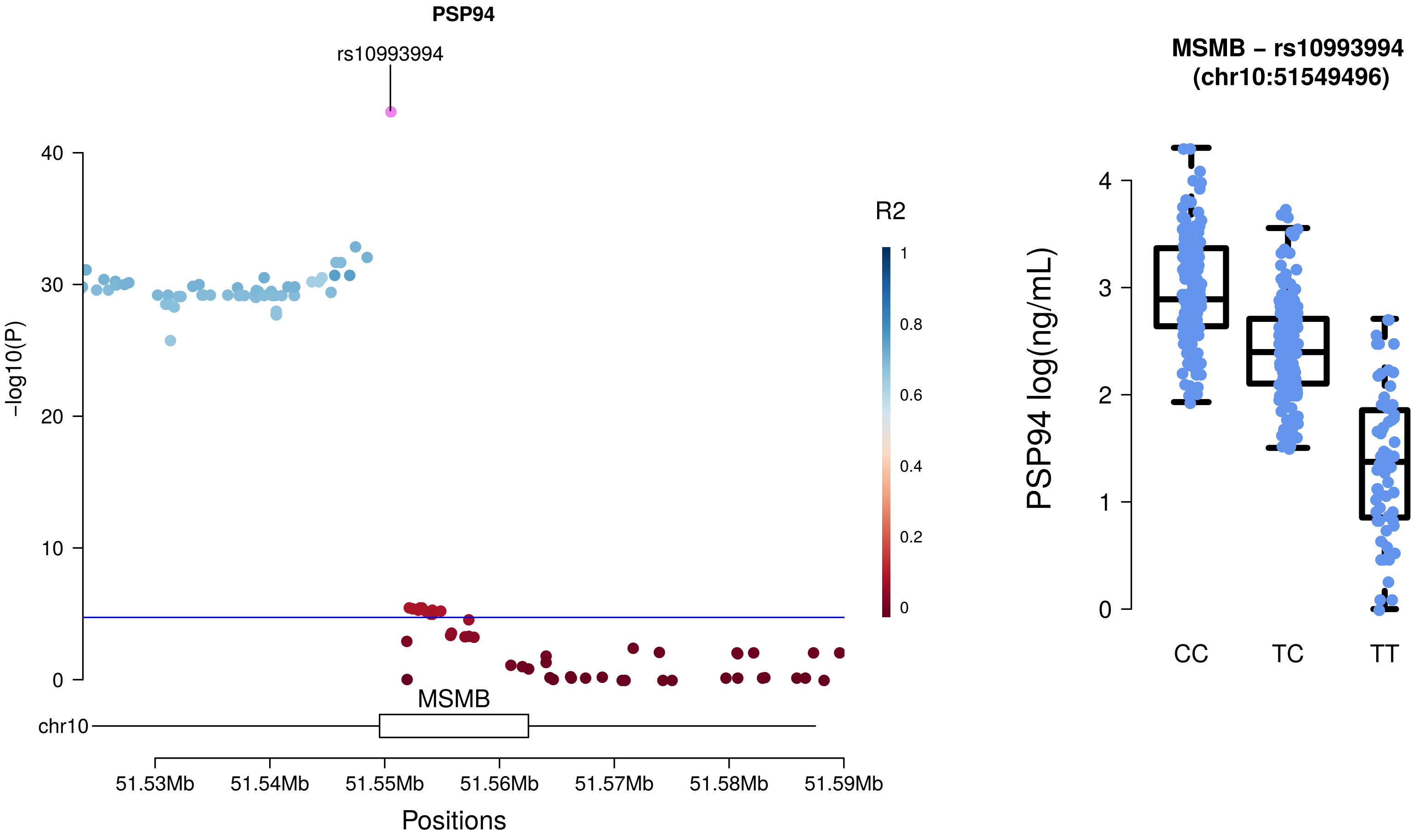

PSP94
rs10993994
-log10(P)
40
30
20
10
0
R2
1
0.8
0.6
0.4
0.2
0
MSMB
chr10
51.53Mb
51.54Mb
51.55Mb
51.56Mb
51.57Mb
51.58Mb
51.59Mb
Positions
MSMB – rs10993994
(chr10:51549496)
PSP94 log(ng/mL)
4
3
2
1
0
CC
TC
TT

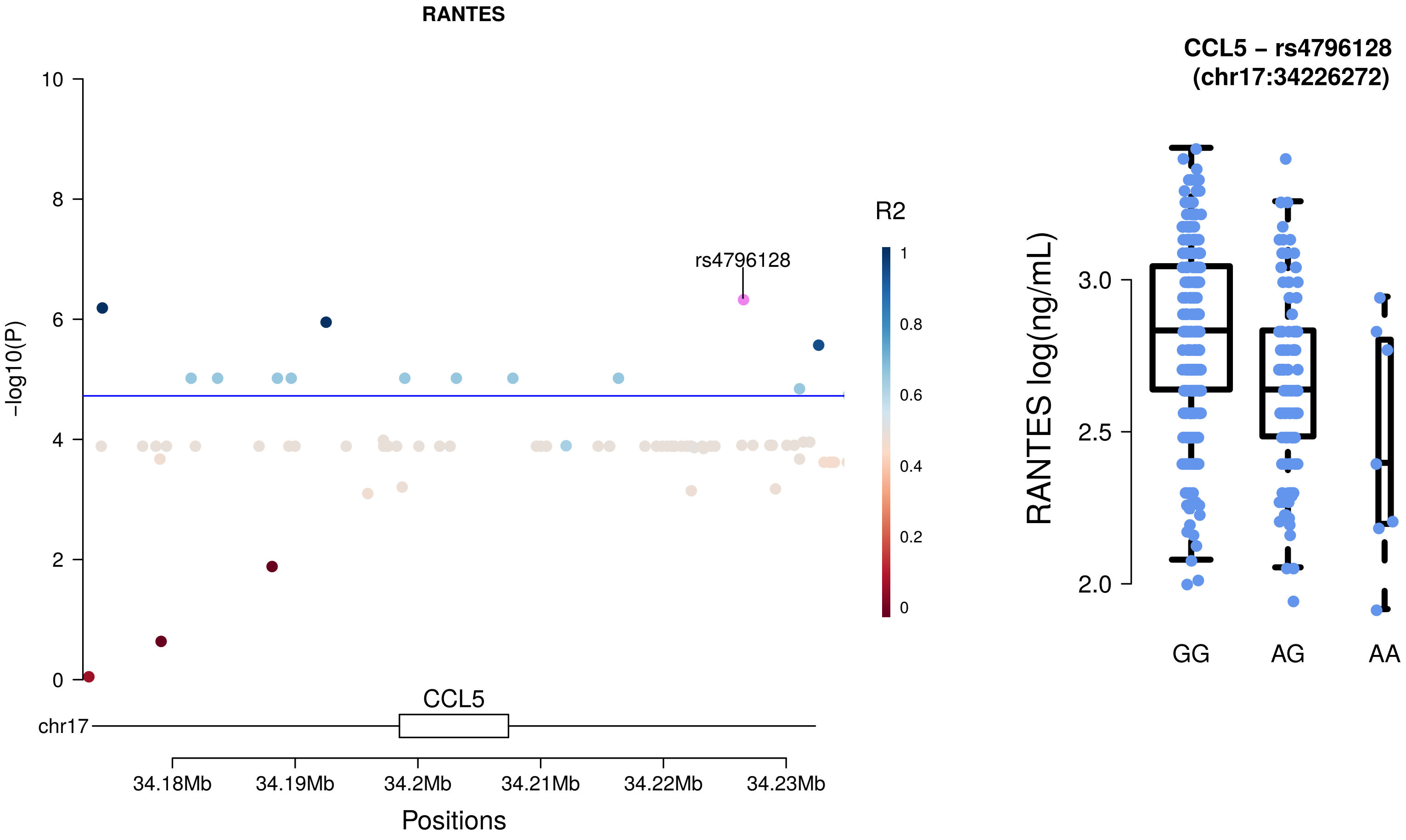
RANTES
rs4796128
R2
1
0.8
0.6
0.4
0.2
0
-log10(P)
10
8
6
4
2
0
CCL5
chr17
34.18Mb
34.19Mb
34.2Mb
34.21Mb
34.22Mb
34.23Mb
Positions
CCL5 – rs4796128
(chr17:34226272)
RANTES log(ng/mL)
3.0
2.5
2.0
GG
AG
AA

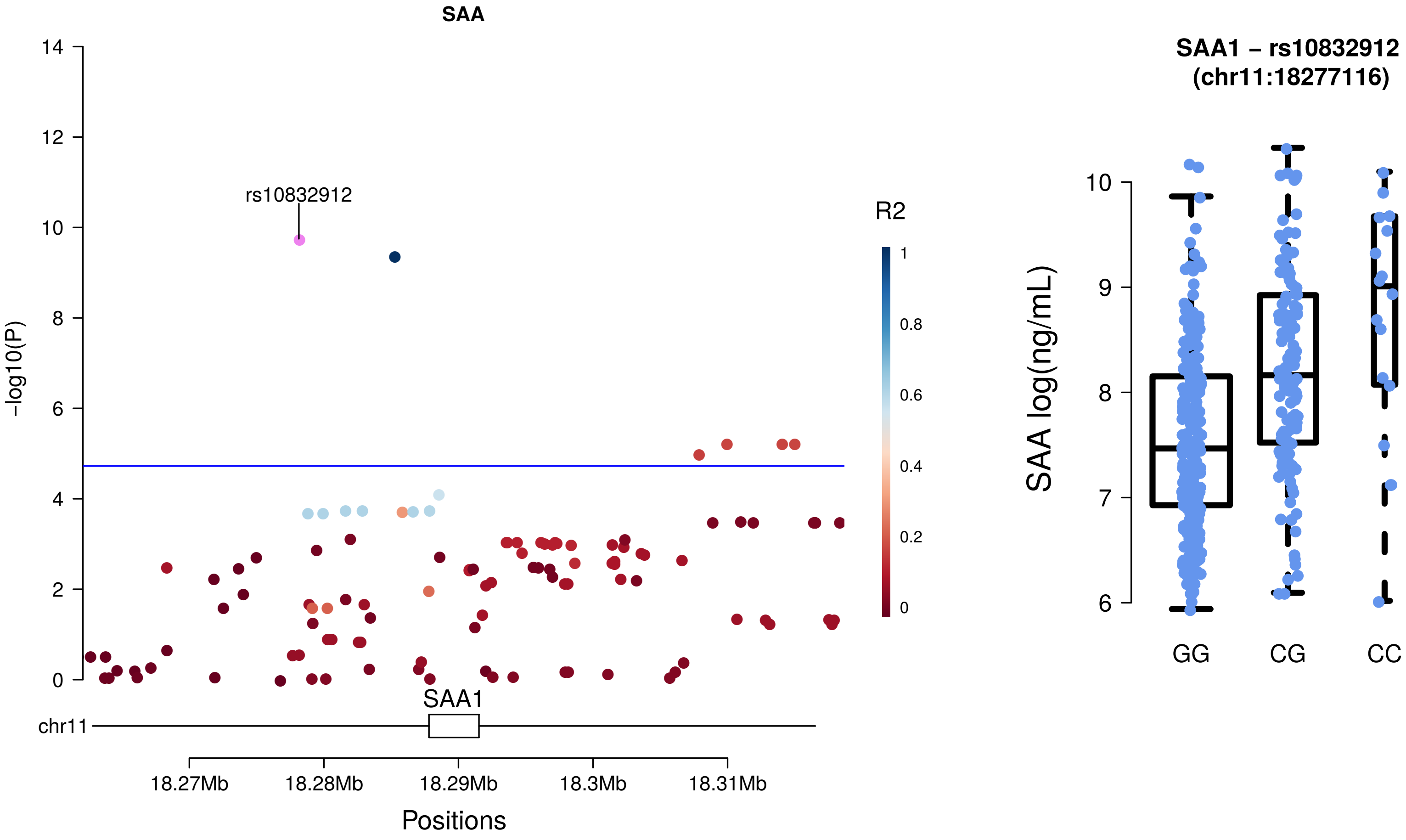

SAA
rs10832912
R2
-log10(P)
SAA1
chr11
18.27Mb
18.28Mb
18.29Mb
18.3Mb
18.31Mb
Positions
SAA1 – rs10832912
(chr11:18277116)
SAA log(ng/mL)
GG
CG
CC

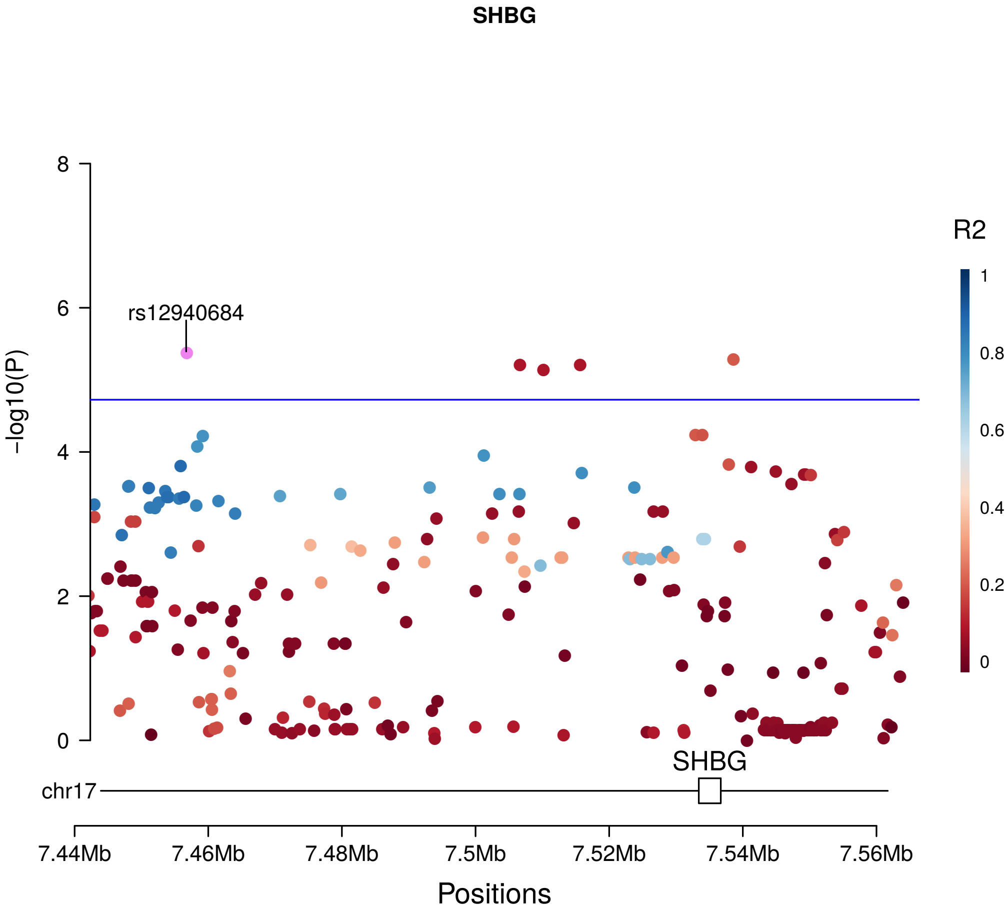
SHBG
R2
1
0.8
0.6
0.4
0.2
0
8
6
4
2
0
-log10(P)
rs12940684
chr17
SHBG
7.44Mb
7.46Mb
7.48Mb
7.5Mb
7.52Mb
7.54Mb
7.56Mb
Positions

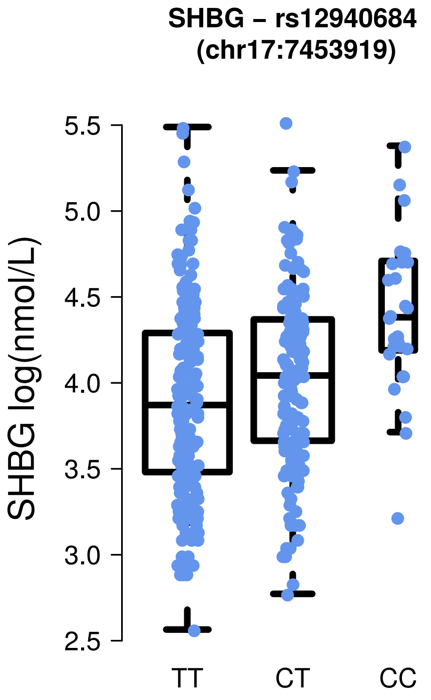
SHBG – rs12940684
(chr17:7453919)
5.5
5.0
4.5
4.0
3.5
3.0
2.5
SHBG log(nmol/L)
TT
CT
CC

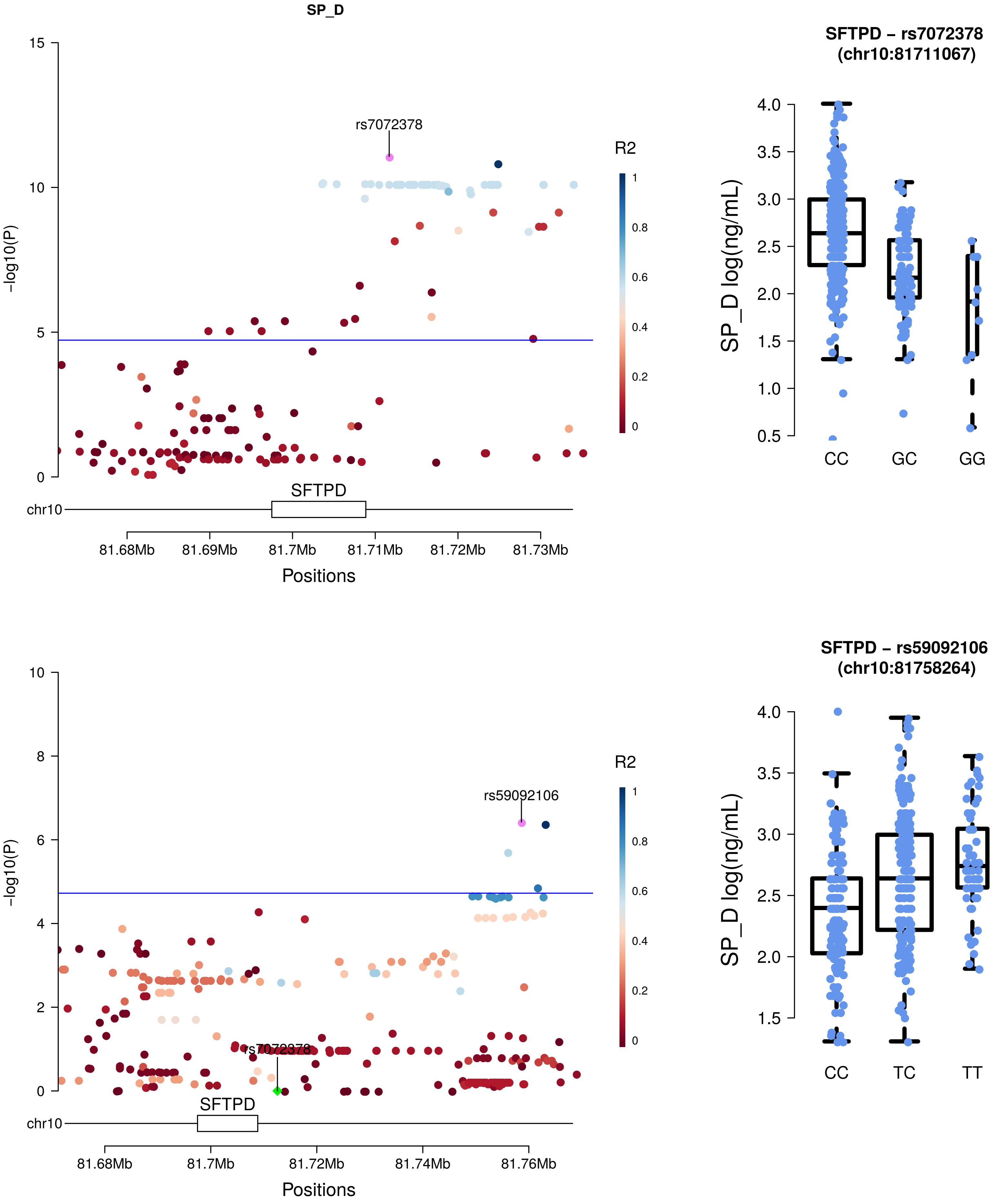
SP_D
rs7072378
R2
-log10(P)
SFTPD
chr10
81.68Mb
81.69Mb
81.7Mb
81.71Mb
81.72Mb
81.73Mb
Positions
SFTPD – rs7072378
(chr10:81711067)
SP_D log(ng/mL)
CC
GC
GG
rs59092106
81.74Mb
81.76Mb
SFTPD – rs59092106
(chr10:81758264)
TC
TT

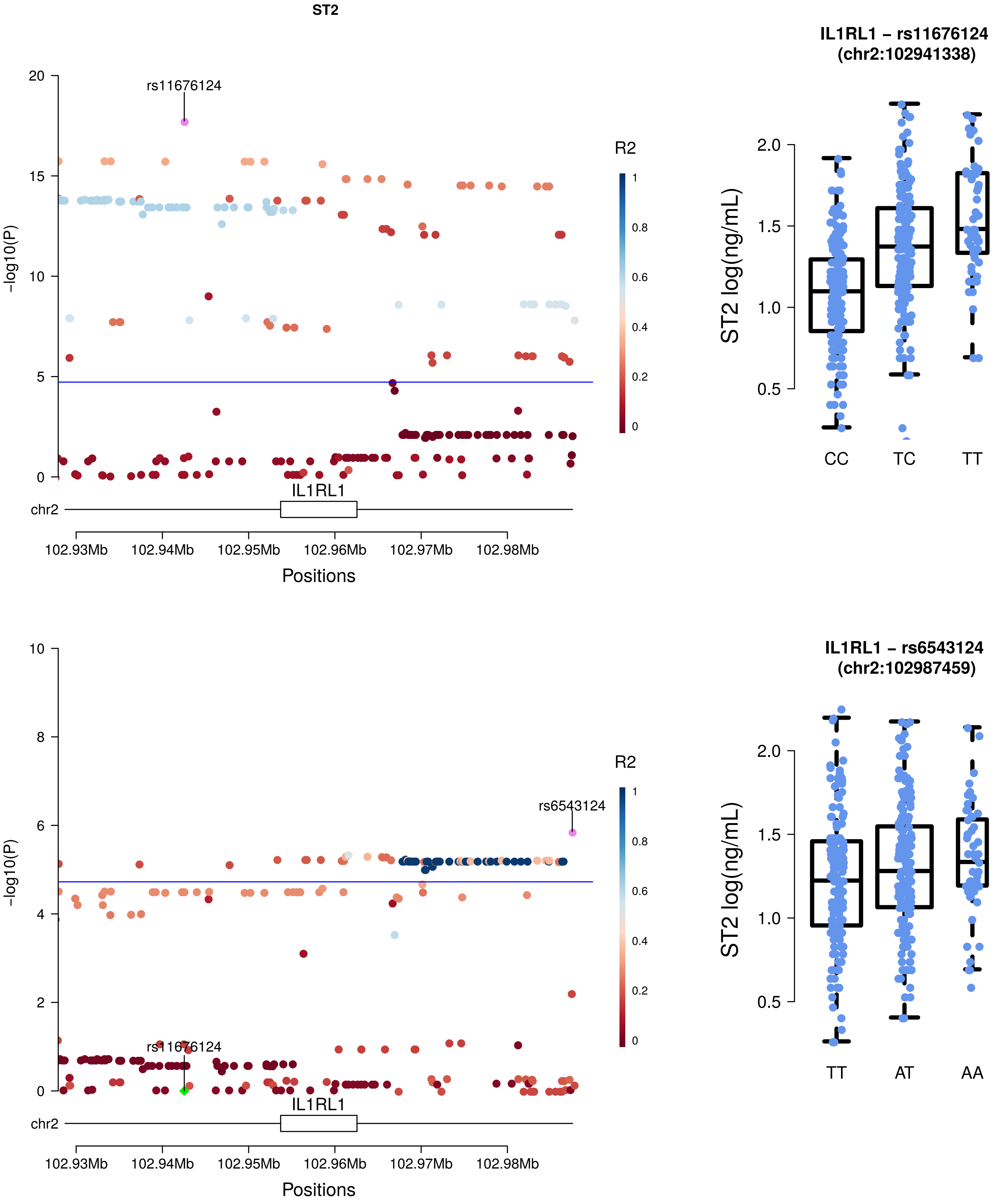

ST2
rs11676124
-log10(P)
R2
IL1RL1
chr2
102.93Mb
102.94Mb
102.95Mb
102.96Mb
102.97Mb
102.98Mb
Positions
IL1RL1 – rs11676124
(chr2:102941338)
ST2 log(ng/mL)
CC
TC
TT
rs6543124
rs11676124
IL1RL1 – rs6543124
(chr2:102987459)
TT
AT
AA

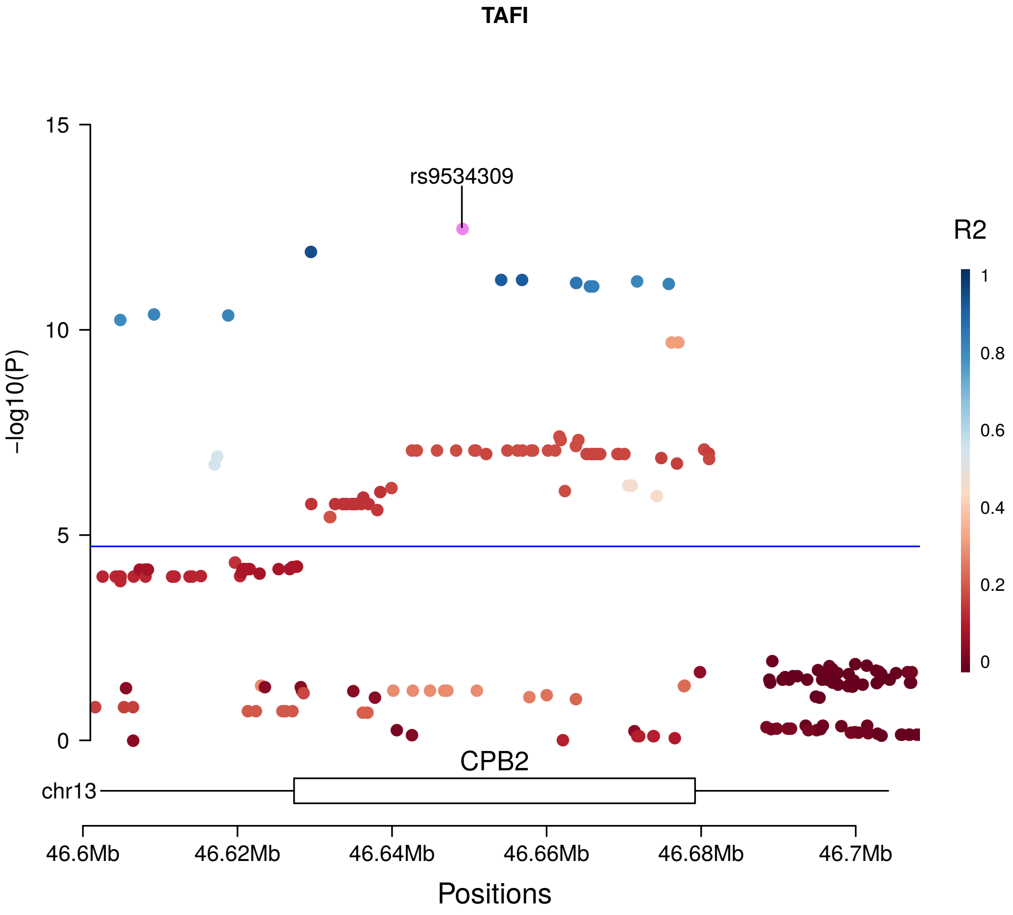
TAFI
rs9534309
R2
1
0.8
0.6
0.4
0.2
0
15
10
5
0
-log10(P)
CPB2
chr13
46.6Mb
46.62Mb
46.64Mb
46.66Mb
46.68Mb
46.7Mb
Positions

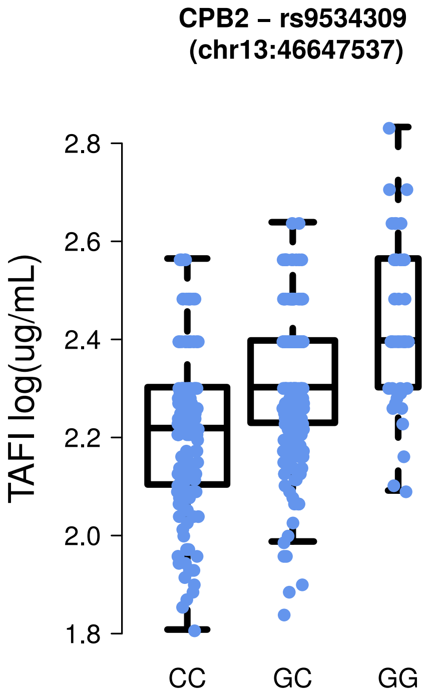
CPB2 – rs9534309
(chr13:46647537)
TAFI log(ug/mL)
2.8
2.6
2.4
2.2
2.0
1.8
CC
GC
GG

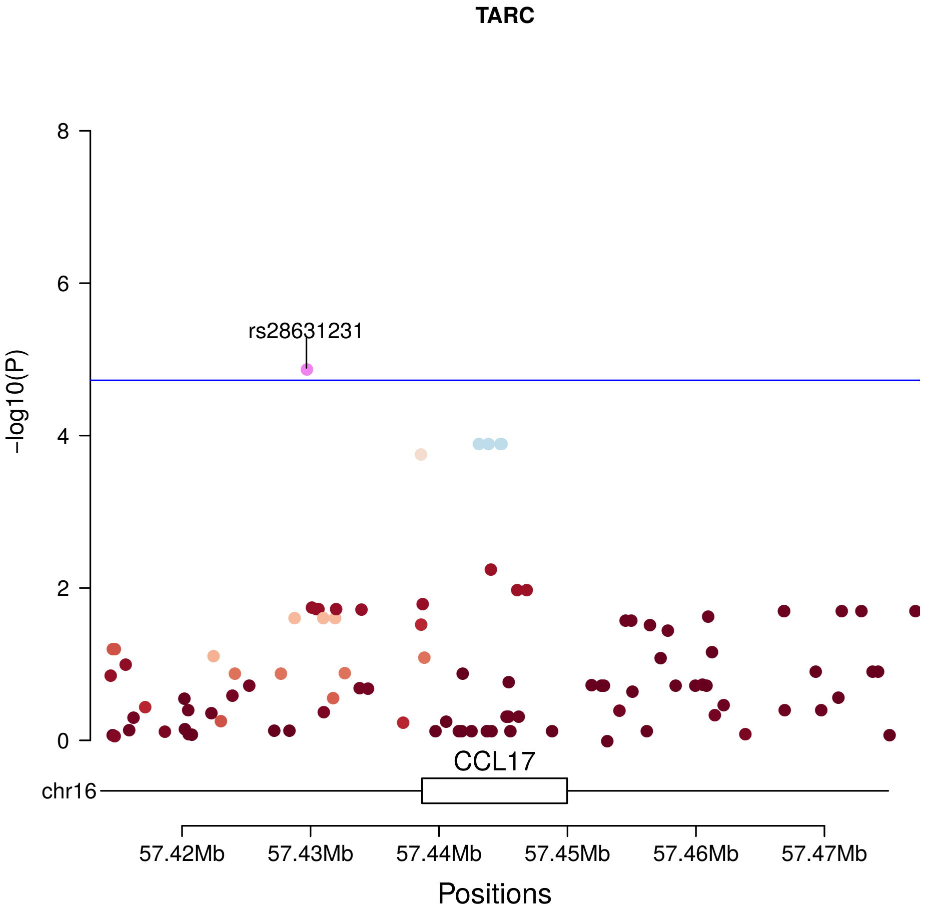
TARC
rs28631231
-log10(P)
8
6
4
2
0
R2
1
0.8
0.6
0.4
0.2
0
CCL17
chr16
57.42Mb
57.43Mb
57.44Mb
57.45Mb
57.46Mb
57.47Mb
Positions

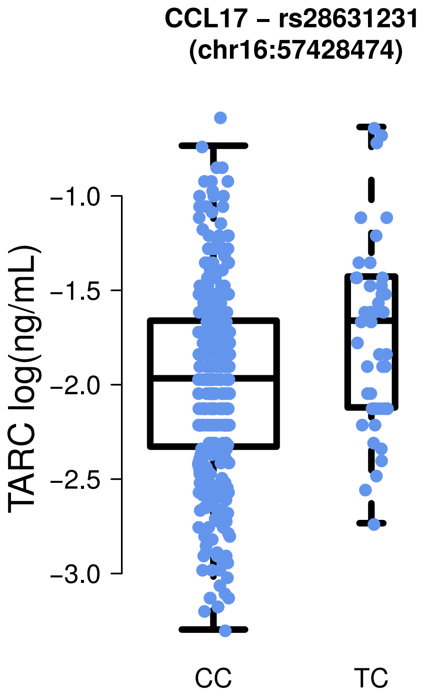
CCL17 – rs28631231
(chr16:57428474)
TARC log(ng/mL)
−1.0
−1.5
−2.0
−2.5
−3.0
CC
TC

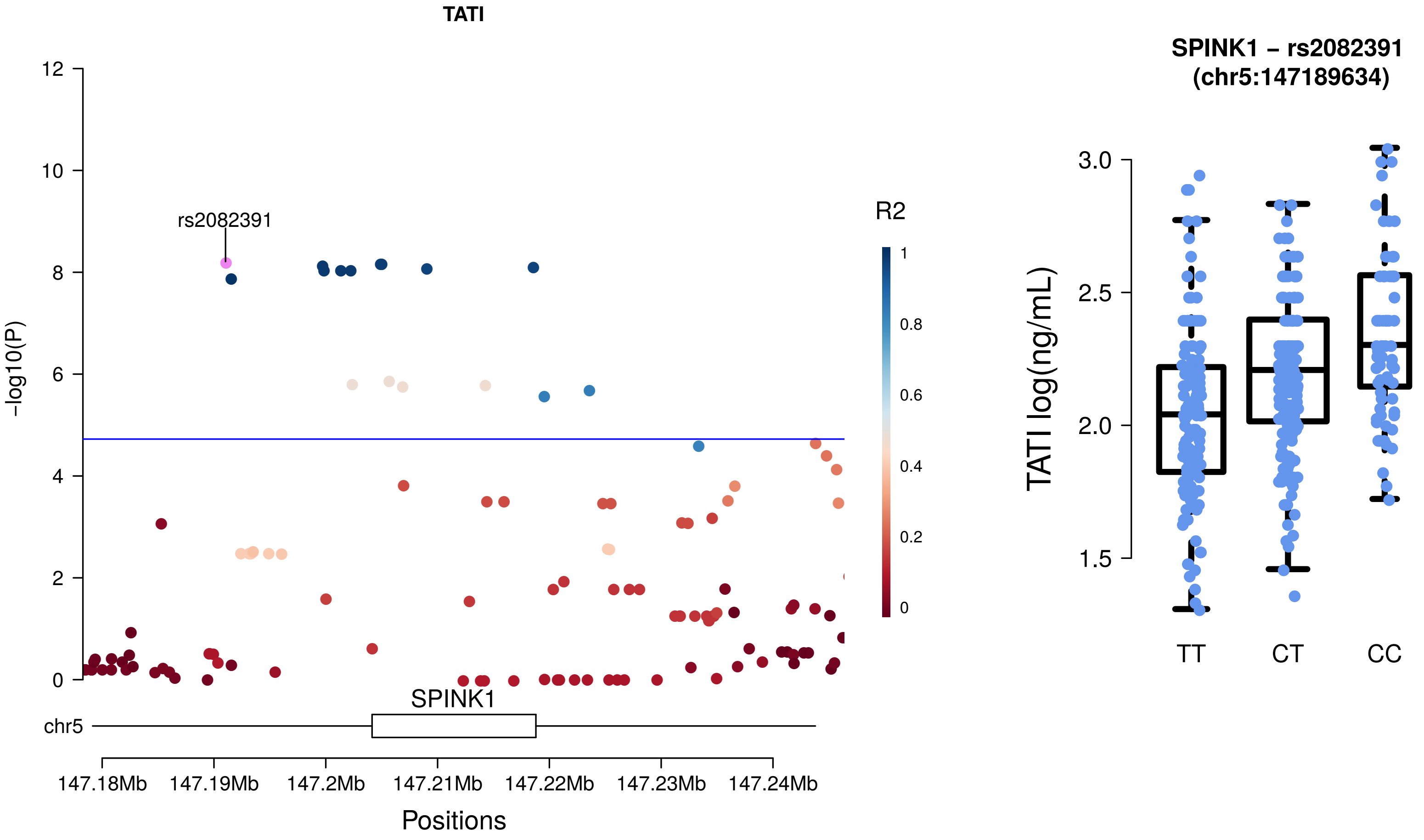

TATI
rs2082391
-log10(P)
R2
SPINK1
chr5
147.18Mb
147.19Mb
147.2Mb
147.21Mb
147.22Mb
147.23Mb
147.24Mb
Positions
SPINK1 – rs2082391
(chr5:147189634)
TATI log(ng/mL)
TT
CT
CC

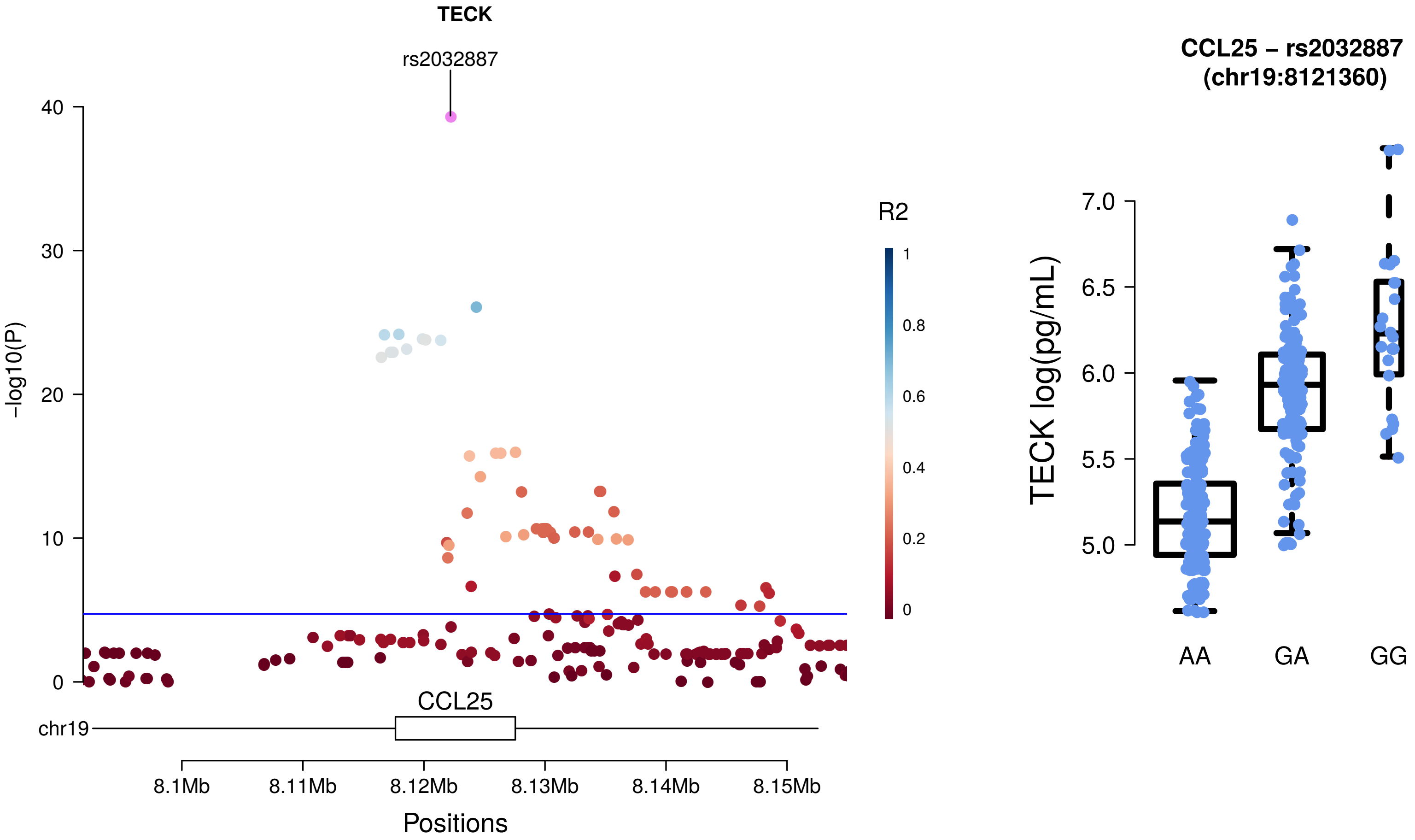
TECK
rs2032887
40
30
20
10
0
-log10(P)
R2
1
0.8
0.6
0.4
0.2
0
CCL25
chr19
8.1Mb
8.11Mb
8.12Mb
8.13Mb
8.14Mb
8.15Mb
Positions
CCL25 – rs2032887
(chr19:8121360)
TECK log(pg/mL)
7.0
6.5
6.0
5.5
5.0
AA
GA
GG

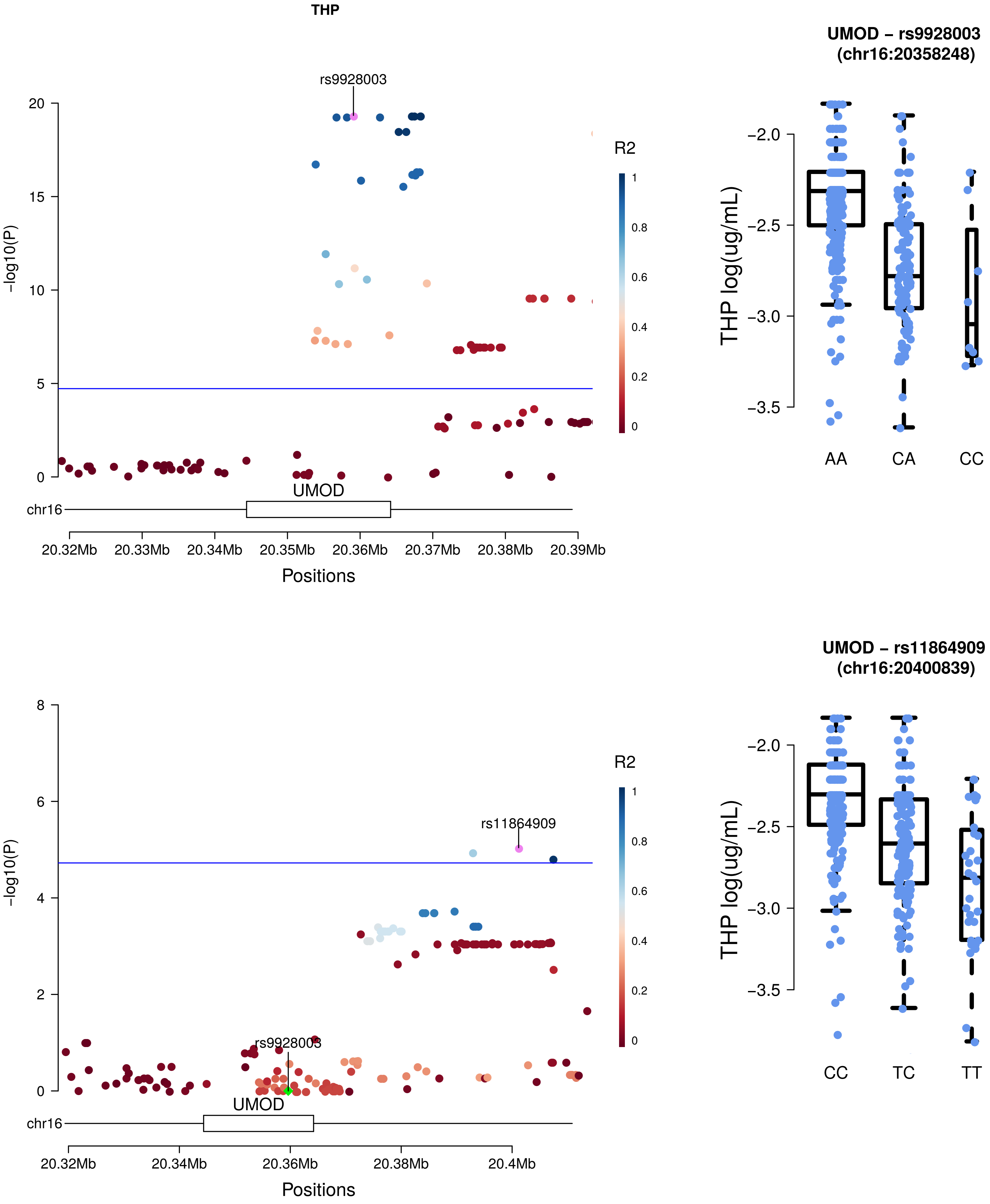
THP
rs9928003
-log10(P)
20
15
10
5
0
R2
1
0.8
0.6
0.4
0.2
0
UMOD
chr16
20.32Mb
20.33Mb
20.34Mb
20.35Mb
20.36Mb
20.37Mb
20.38Mb
20.39Mb
Positions
UMOD – rs9928003
(chr16:20358248)
THP log(ug/mL)
−2.0
−2.5
−3.0
−3.5
AA
CA
CC
-log10(P)
8
6
4
2
0
rs11864909
rs9928003
R2
1
0.8
0.6
0.4
0.2
0
UMOD
chr16
20.32Mb
20.34Mb
20.36Mb
20.38Mb
20.4Mb
Positions
UMOD – rs11864909
(chr16:20400839)
THP log(ug/mL)
−2.0
−2.5
−3.0
−3.5
CC
TC
TT

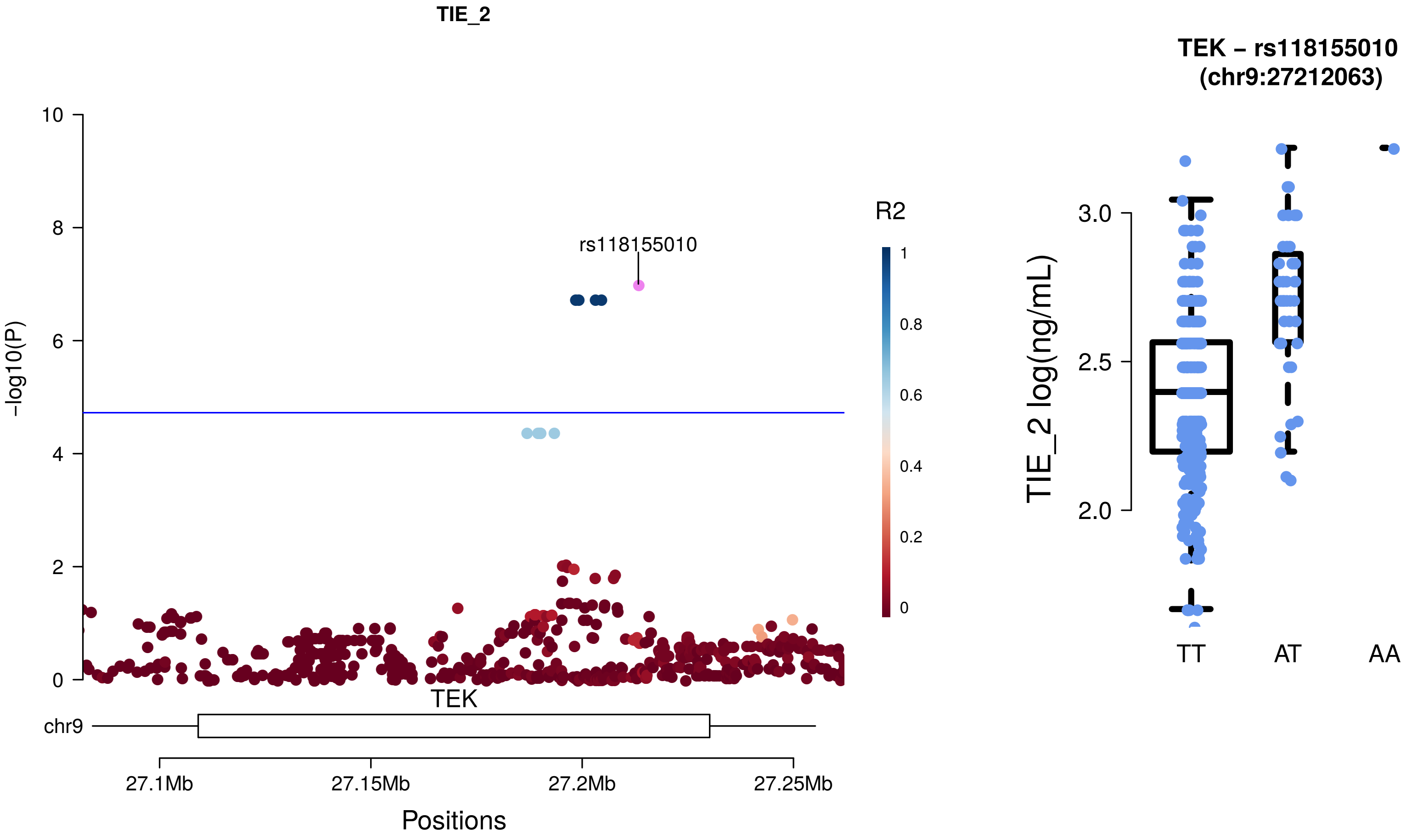

TIE_2
TEK – rs118155010
(chr9:27212063)
10
8
6
4
2
0
-log10(P)
rs118155010
R2
1
0.8
0.6
0.4
0.2
0
TEK
chr9
27.1Mb
27.15Mb
27.2Mb
27.25Mb
Positions
TIE_2 log(ng/mL)
3.0
2.5
2.0
TT
AT
AA

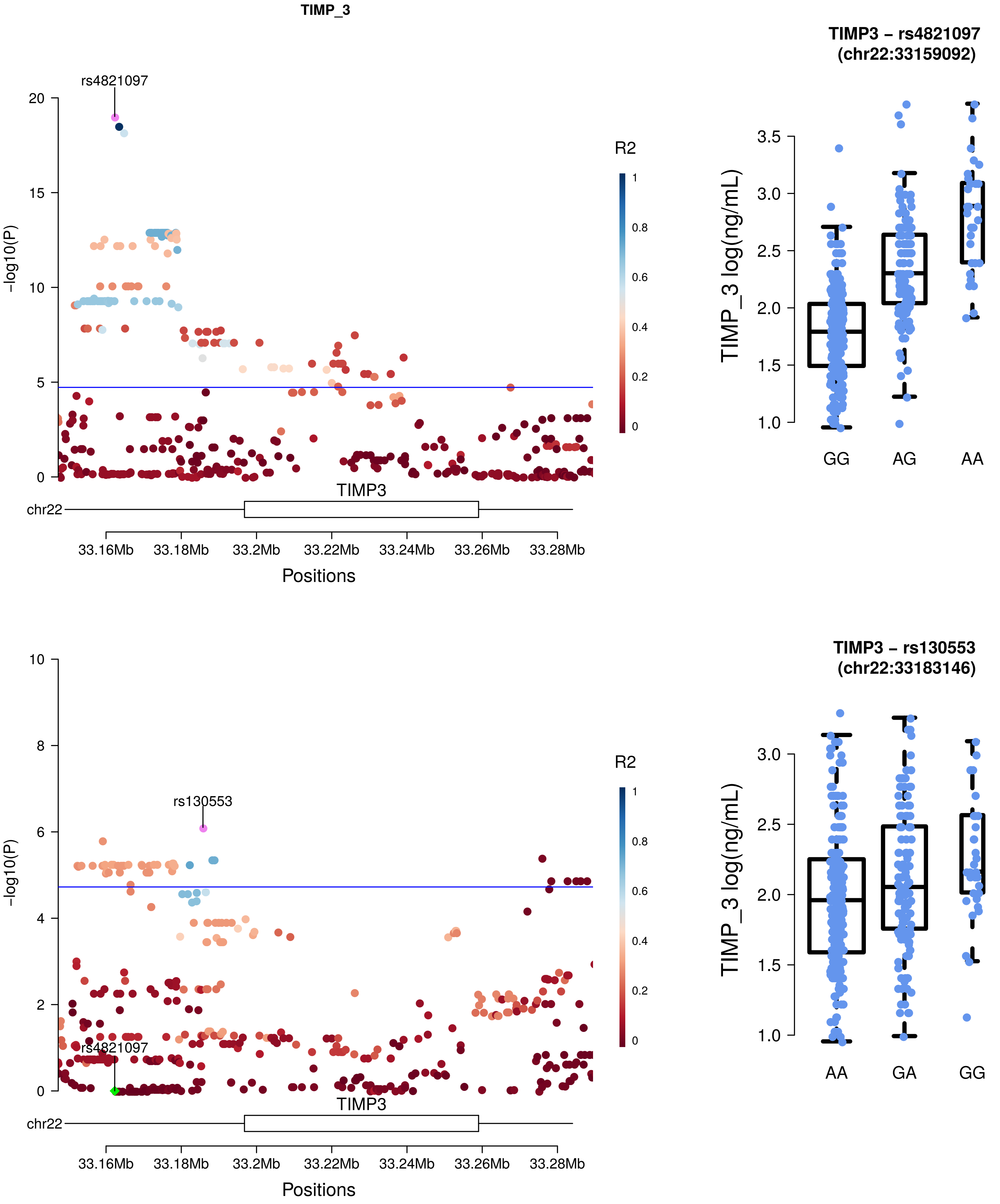
TIMP_3
rs4821097
-log10(P)
R2
TIMP3
chr22
33.16Mb
33.18Mb
33.2Mb
33.22Mb
33.24Mb
33.26Mb
33.28Mb
Positions
TIMP3 – rs4821097
(chr22:33159092)
TIMP_3 log(ng/mL)
GG
AG
AA
rs130553
rs4821097
TIMP3 – rs130553
(chr22:33183146)
AA
GA
GG

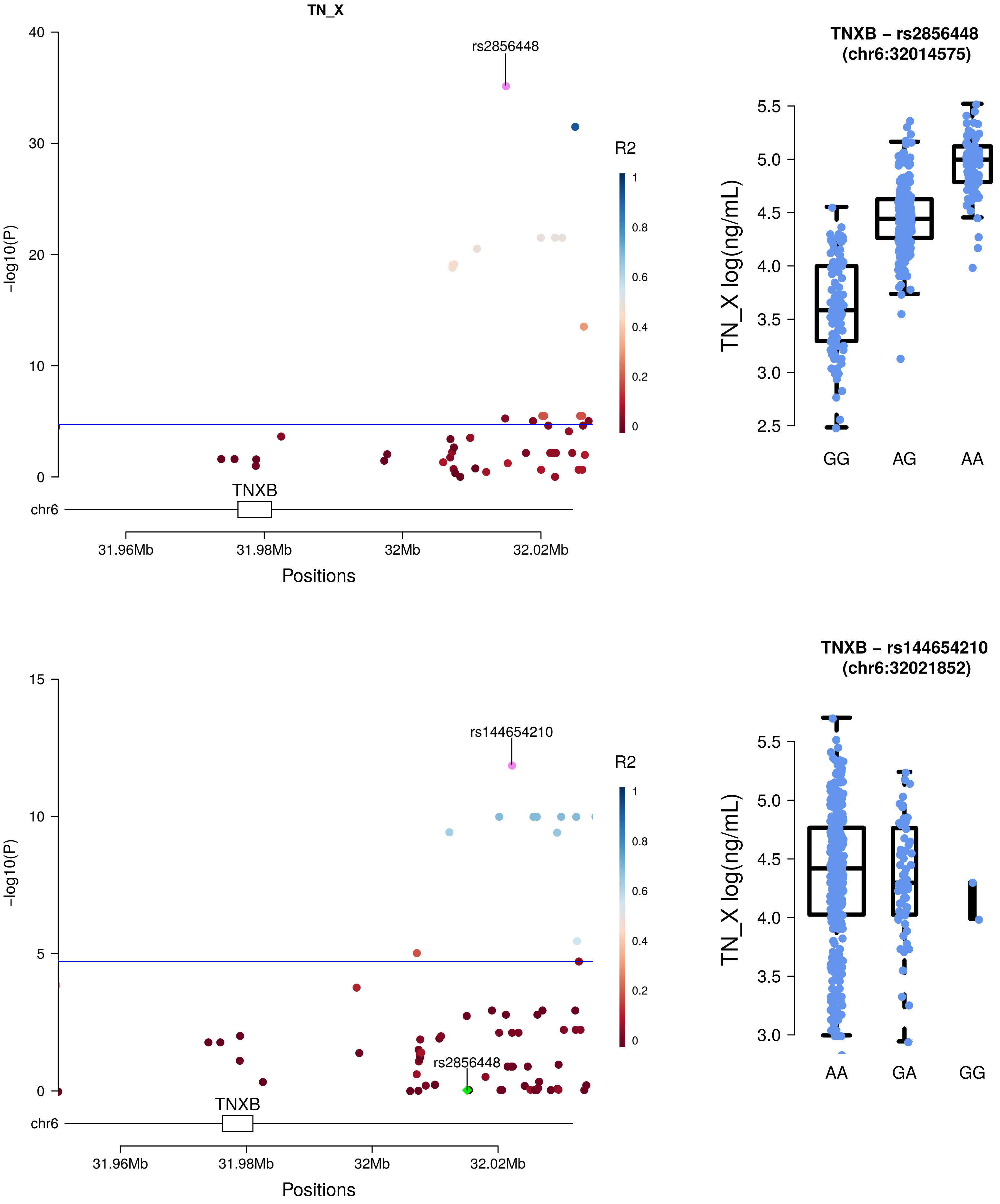
TN_X
rs2856448
R2
-log10(P)
TNXB
chr6
31.96Mb
31.98Mb
32Mb
32.02Mb
Positions
TNXB – rs2856448
(chr6:32014575)
TN_X log(ng/mL)
GG
AG
AA
rs144654210
rs2856448
TNXB – rs144654210
(chr6:32021852)
AA
GA
GG

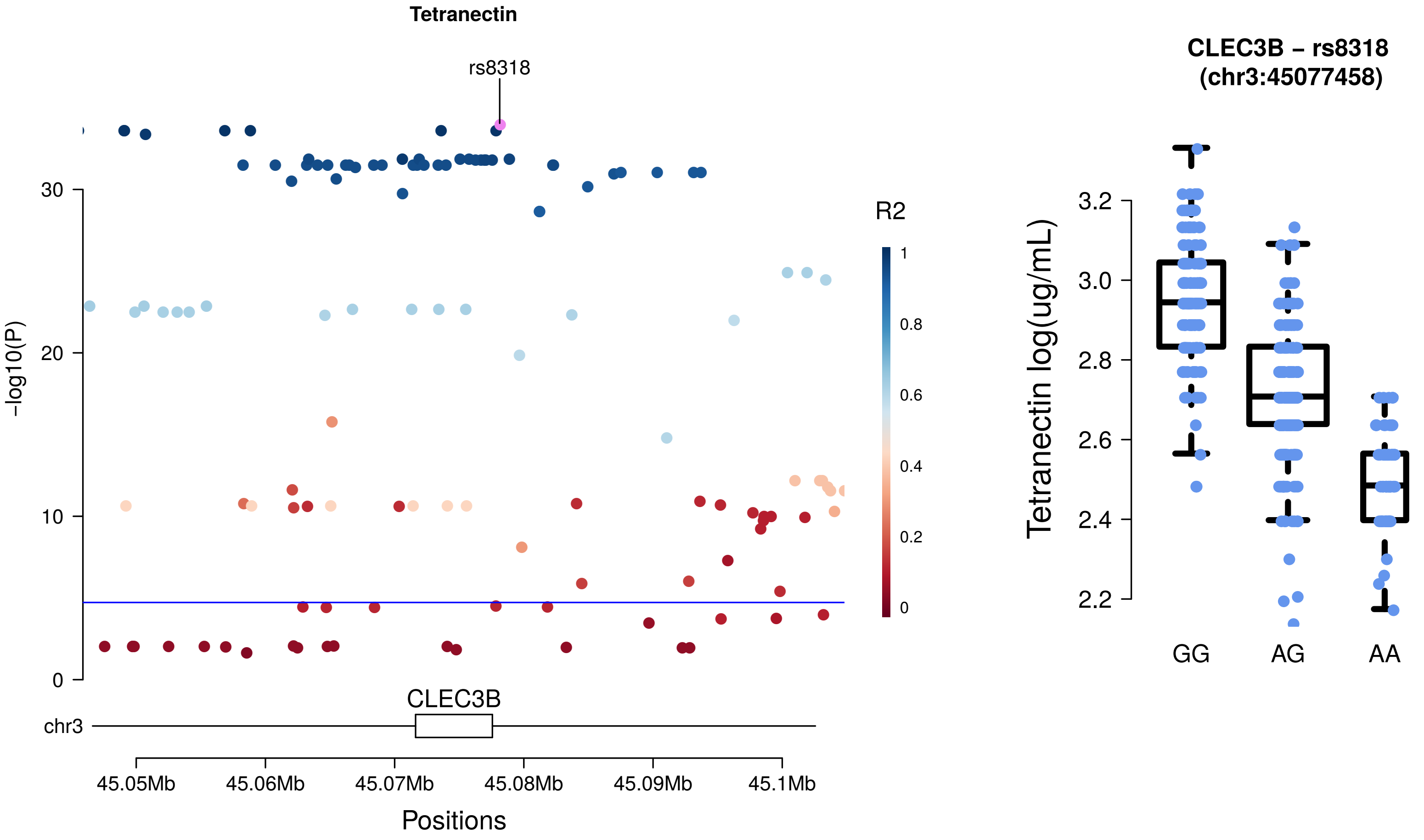
Tetranectin
rs8318
-log10(P)
30
20
10
0
R2
1
0.8
0.6
0.4
0.2
0
CLEC3B
chr3
45.05Mb
45.06Mb
45.07Mb
45.08Mb
45.09Mb
45.1Mb
Positions
CLEC3B – rs8318
(chr3:45077458)
Tetranectin log(ug/mL)
3.2
3.0
2.8
2.6
2.4
2.2
GG
AG
AA

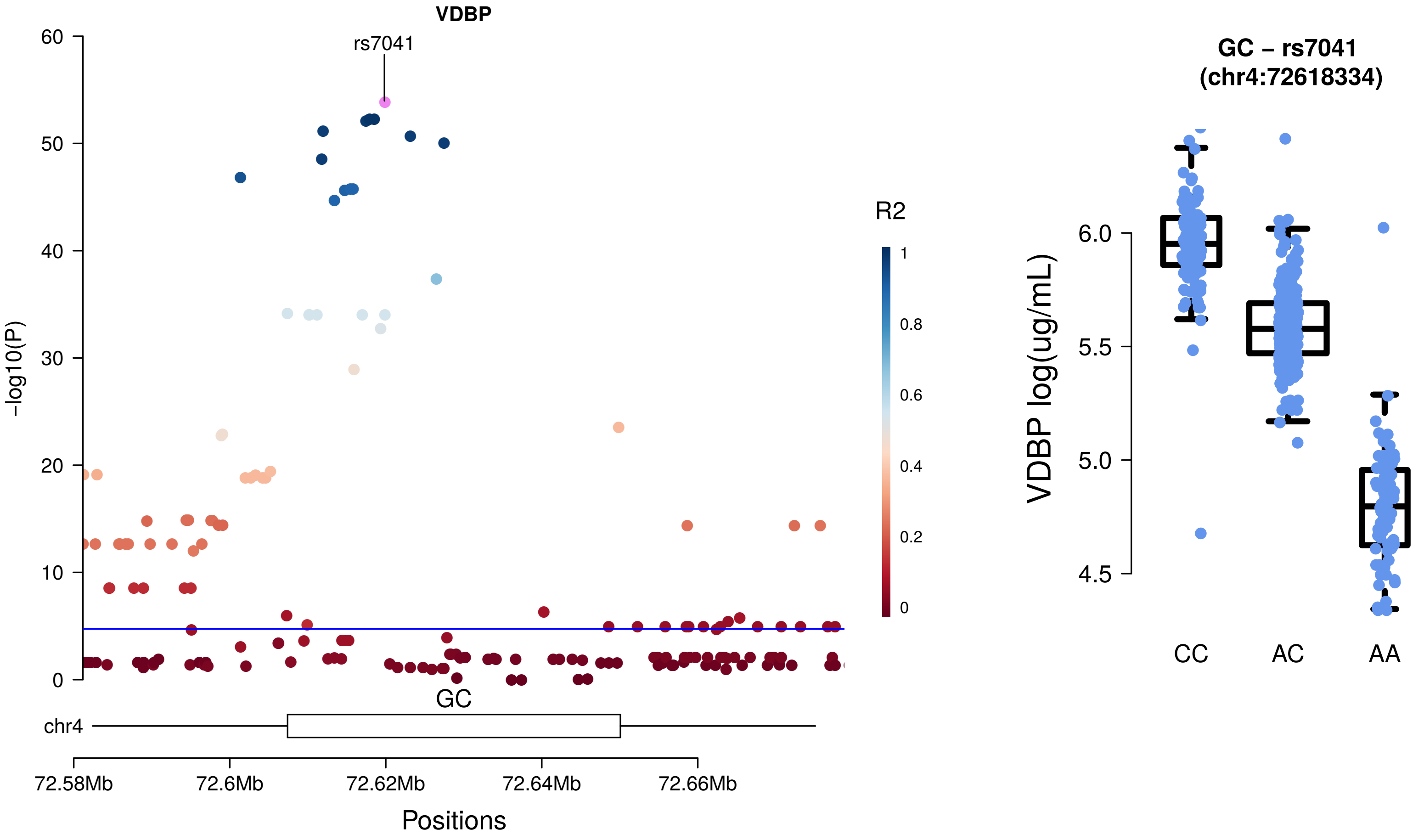
VDBP
rs7041
-log10(P)
60
50
40
30
20
10
0
R2
1
0.8
0.6
0.4
0.2
0
GC
chr4
72.58Mb
72.6Mb
72.62Mb
72.64Mb
72.66Mb
Positions
GC – rs7041
(chr4:72618334)
VDBP log(ug/mL)
6.0
5.5
5.0
4.5
CC
AC
AA

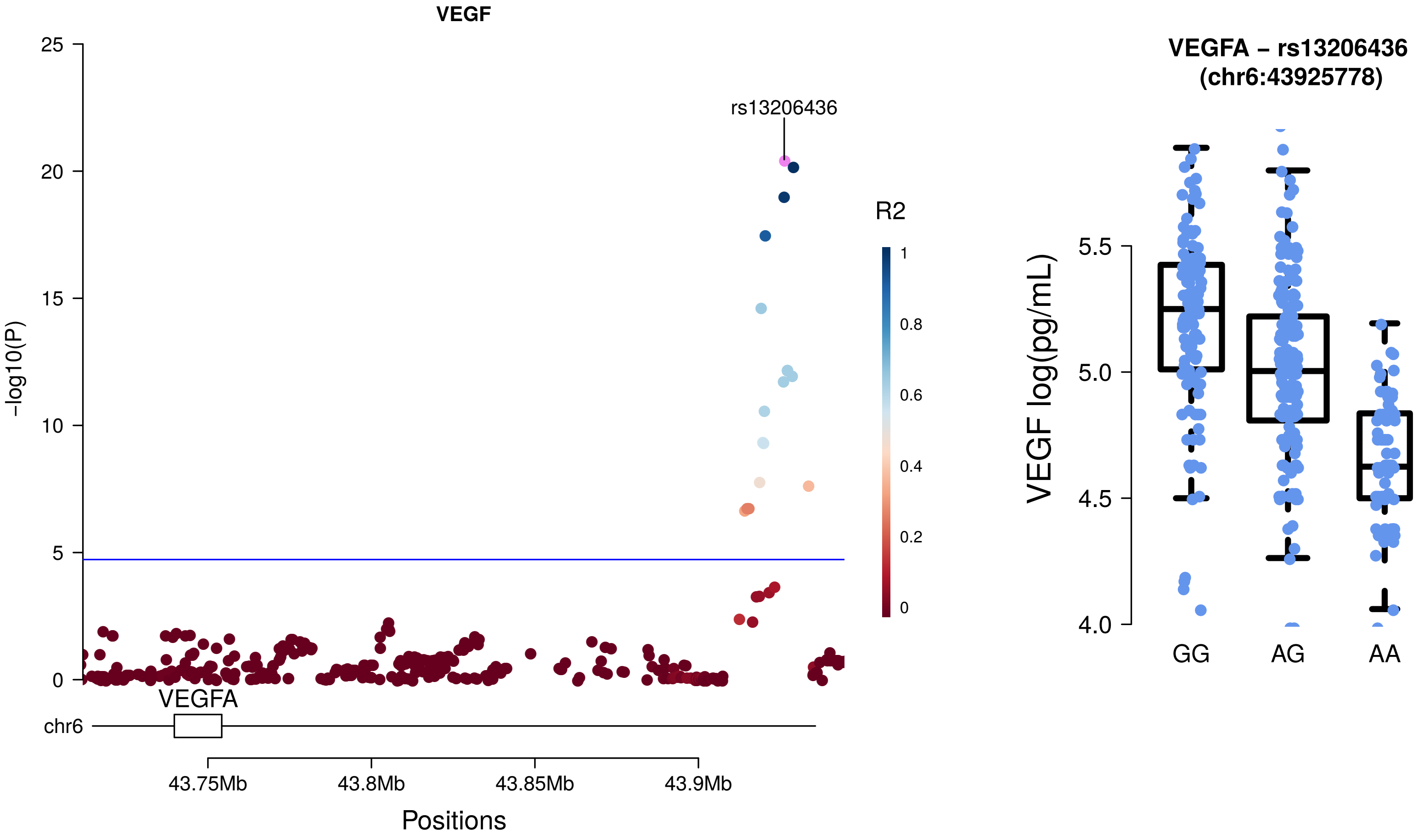

VEGF
rs13206436
-log10(P)
25
20
15
10
5
0
R2
1
0.8
0.6
0.4
0.2
0
VEGFA
chr6
43.75Mb
43.8Mb
43.85Mb
43.9Mb
Positions
VEGFA – rs13206436
(chr6:43925778)
VEGF log(pg/mL)
5.5
5.0
4.5
4.0
GG
AG
AA

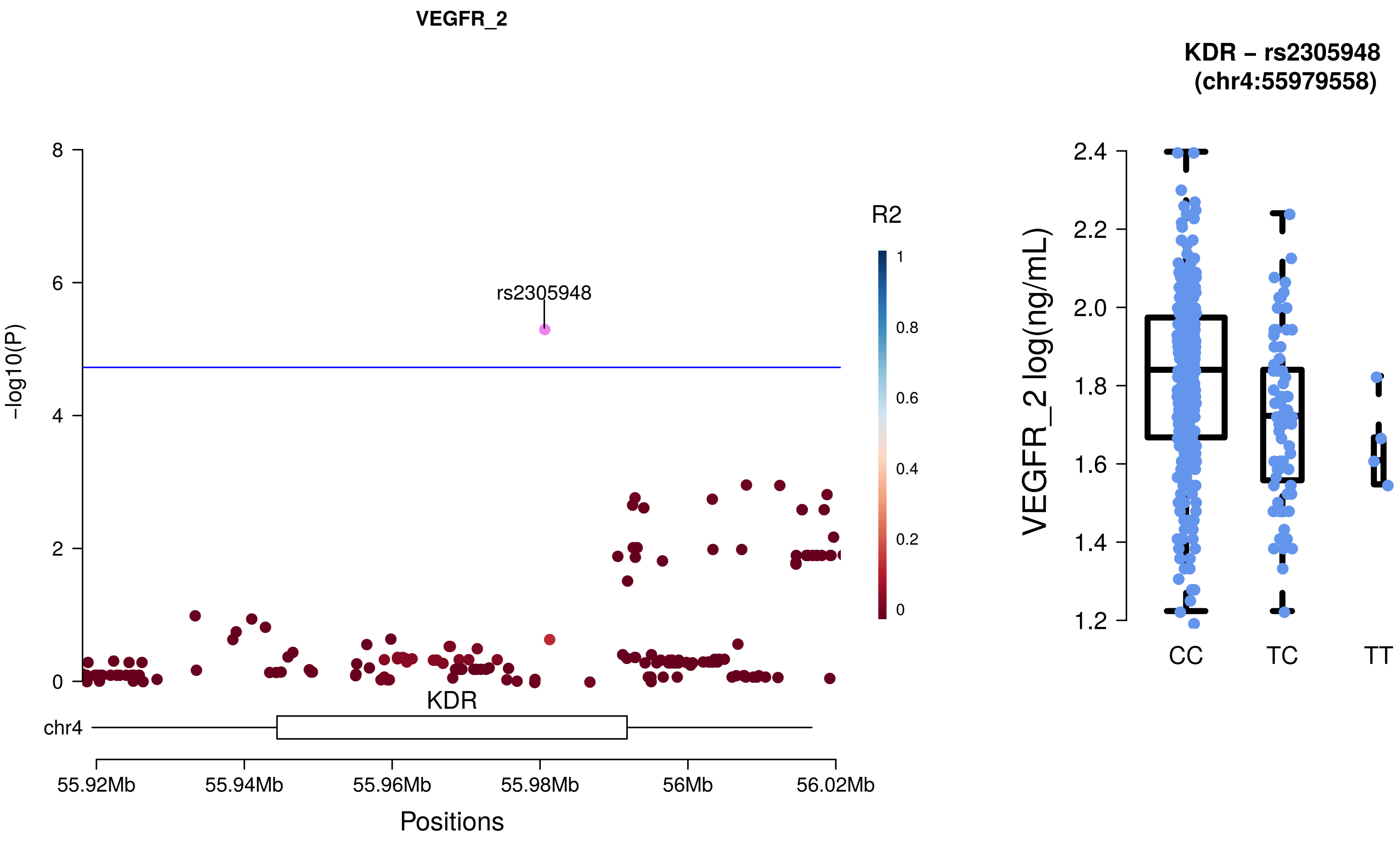
VEGFR_2
rs2305948
R2
1
0.8
0.6
0.4
0.2
0
-log10(P)
8
6
4
2
0
KDR
chr4
55.92Mb
55.94Mb
55.96Mb
55.98Mb
56Mb
56.02Mb
Positions
KDR – rs2305948
(chr4:55979558)
VEGFR_2 log(ng/mL)
2.4
2.2
2.0
1.8
1.6
1.4
1.2
CC
TC
TT

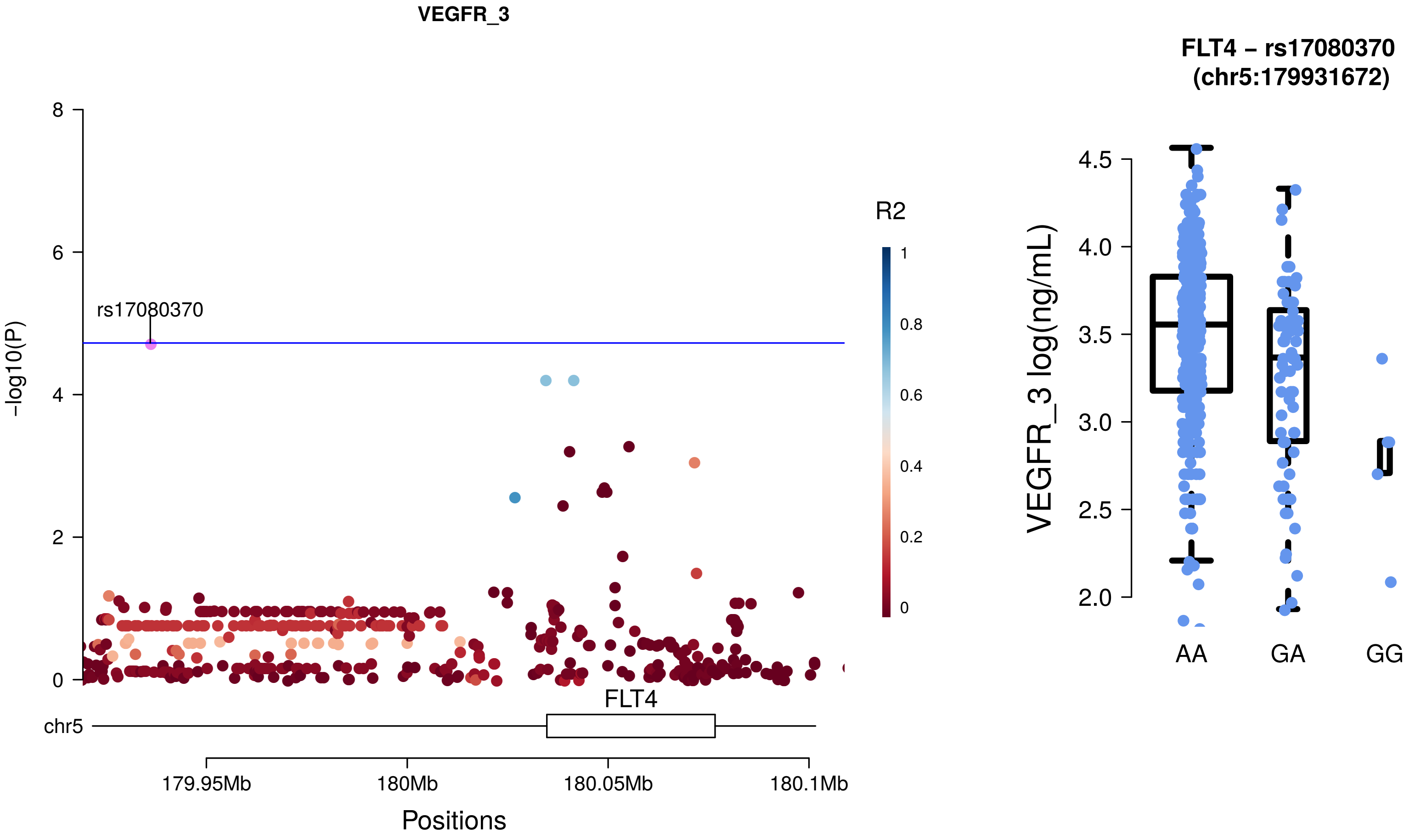
VEGFR_3
rs17080370
-log10(P)
R2
1
0.8
0.6
0.4
0.2
0
FLT4
chr5
179.95Mb
180Mb
180.05Mb
180.1Mb
Positions
FLT4 – rs17080370
(chr5:179931672)
VEGFR_3 log(ng/mL)
4.5
4.0
3.5
3.0
2.5
2.0
AA
GA
GG

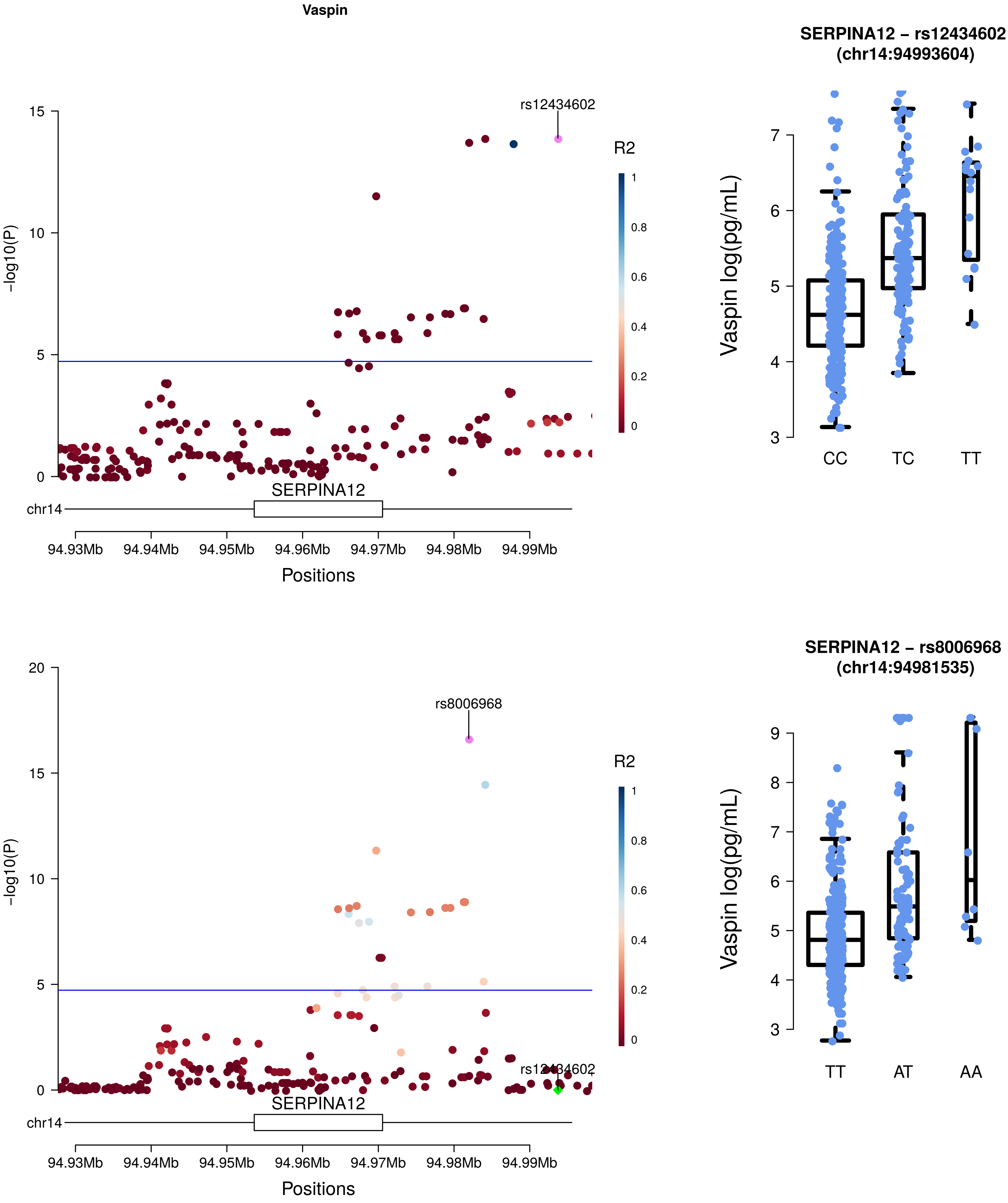

Vaspin
rs12434602
-log10(P)
R2
SERPINA12
chr14
94.93Mb 94.94Mb 94.95Mb 94.96Mb 94.97Mb 94.98Mb 94.99Mb
Positions
SERPINA12 – rs12434602
(chr14:94993604)
Vaspin log(pg/mL)
CC TC TT
rs8006968
rs12434602
SERPINA12 – rs8006968
(chr14:94981535)
TT AT AA

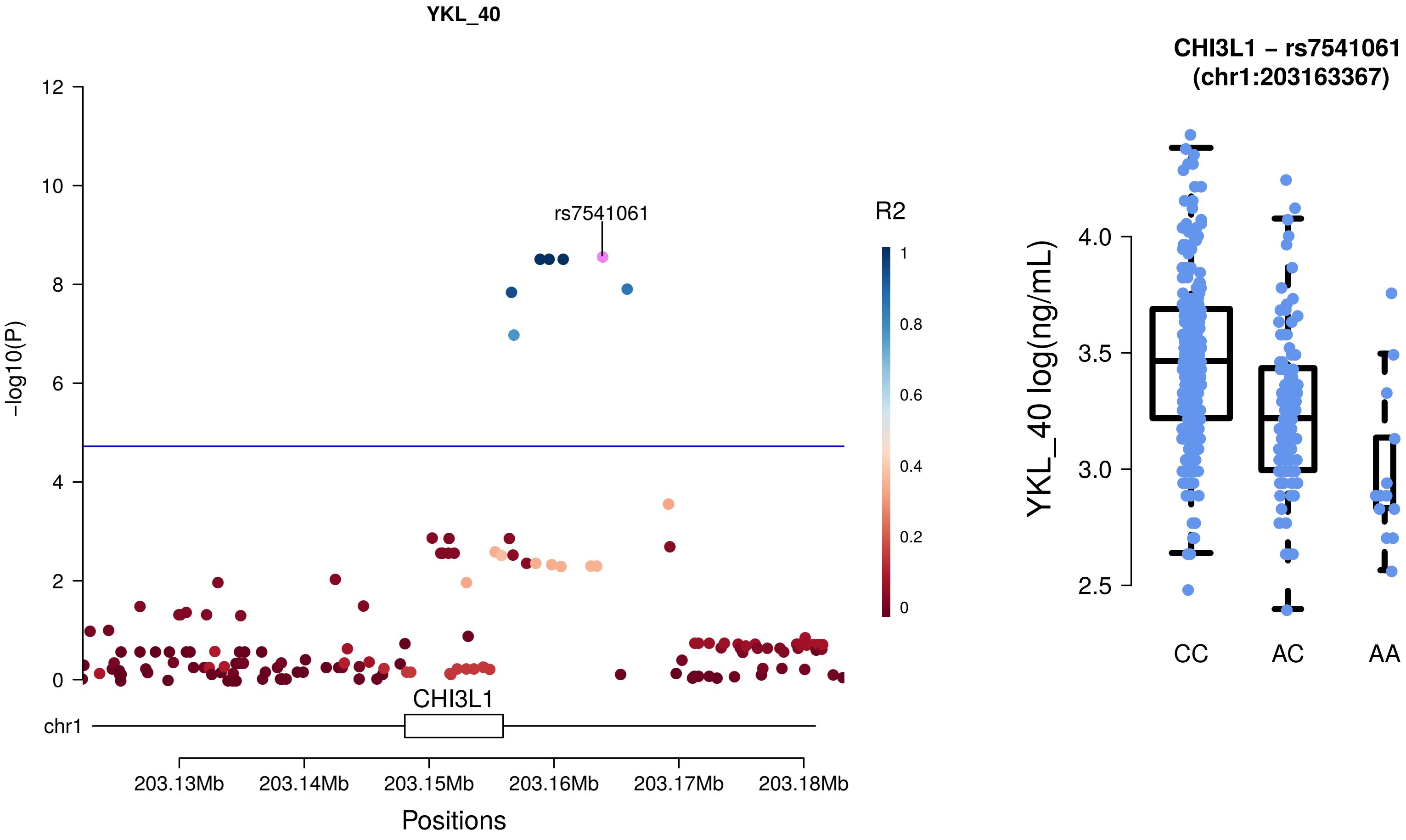

YKL_40
rs7541061
R2
-log10(P)
CHI3L1
chr1
203.13Mb
203.14Mb
203.15Mb
203.16Mb
203.17Mb
203.18Mb
Positions
CHI3L1 – rs7541061
(chr1:203163367)
YKL_40 log(ng/mL)
CC
AC
AA

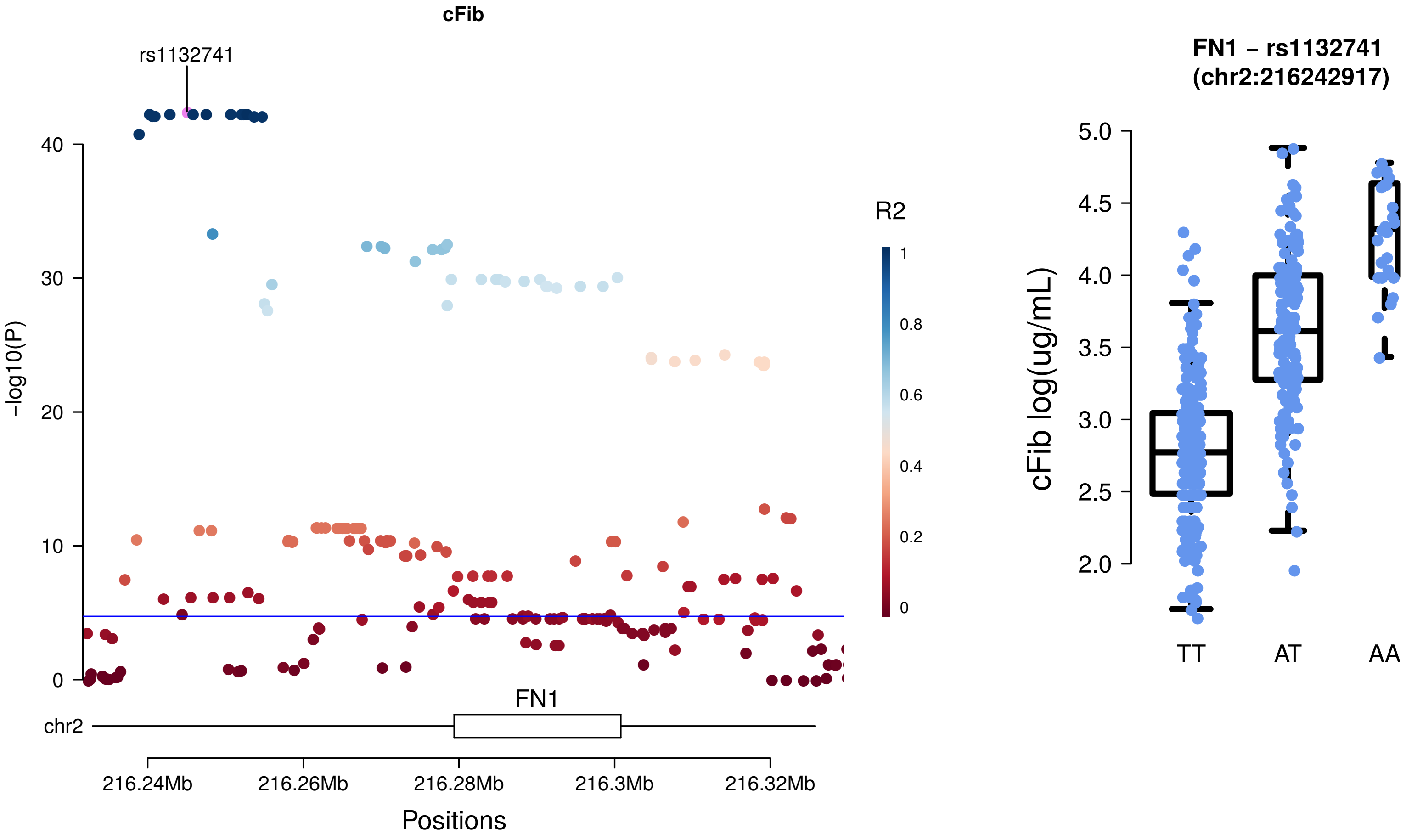
cFib
rs1132741
-log10(P)
40
30
20
10
0
R2
1
0.8
0.6
0.4
0.2
0
FN1
chr2
216.24Mb
216.26Mb
216.28Mb
216.3Mb
216.32Mb
Positions
FN1 – rs1132741
(chr2:216242917)
cFib log(ug/mL)
5.0
4.5
4.0
3.5
3.0
2.5
2.0
TT
AT
AA

Figure S3: Manhattan plots and allelic expression of levels of trans-pQTLs

Manhattan plots showing the distribution of genotyped and imputed SNPs across the 22 autosomes for the proteins with trans-pQTL identified in our study. The chromosomes are represented on the x axis, while the y axis represent the -log10 p-value of association, each dot corresponding to a SNP. The sentinel SNP is colored in pink and is identified by its dbsnp id. The other SNPs are colored based on their chromosome. The horizontal blue line represent the p value threshold corresponding to the trans-FDR level. Additionally, the expression levels of the two homozygous states and the heterozygous state of the corresponding trans-pQTLs are represented on the right, each dot corresponding to the log transformed plasma levels of an individual. If a second trans-pQTL was found during the conditional analysis, it is represented as a second pair of Manhattan and allelic expression plots. The Manhattan plots representing conditional trans-pQTLs are showing the conditional sentinel SNP in pink, while the previously identified sentinel SNP is represented in green. Both are labeled with their dbsnp id.

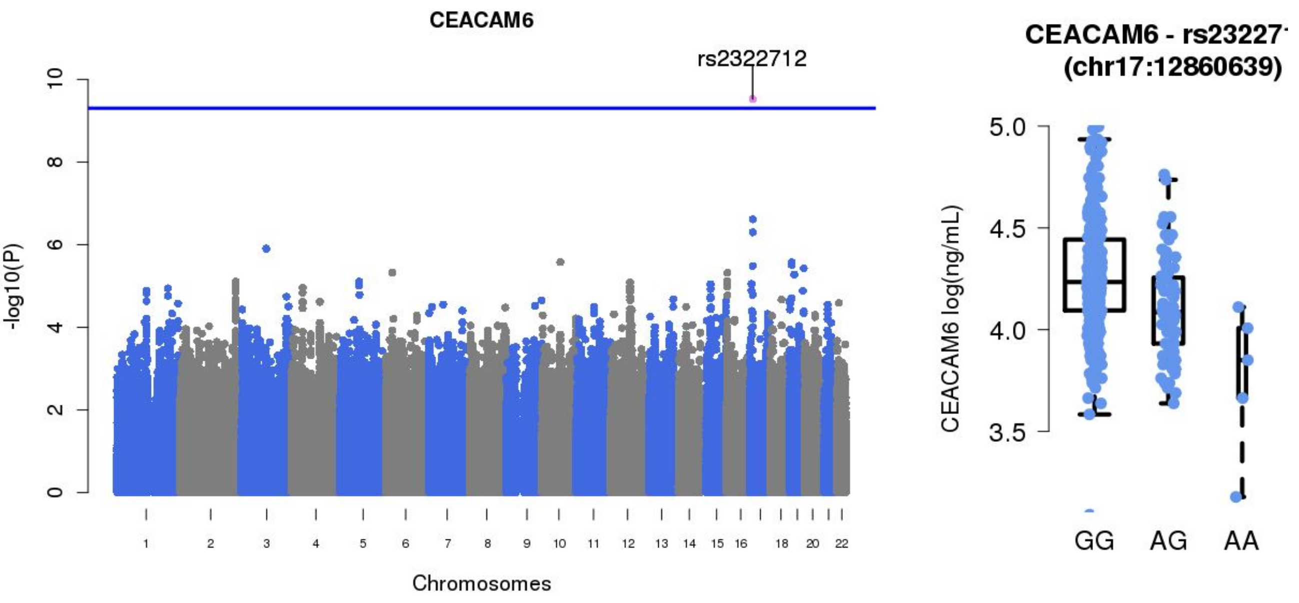

CEACAM6
rs2322712
-log10(P)
Chromosomes
CEACAM6 - rs23227
(chr17:12860639)
CEACAM6 log(ng/mL)
GG
AG
AA

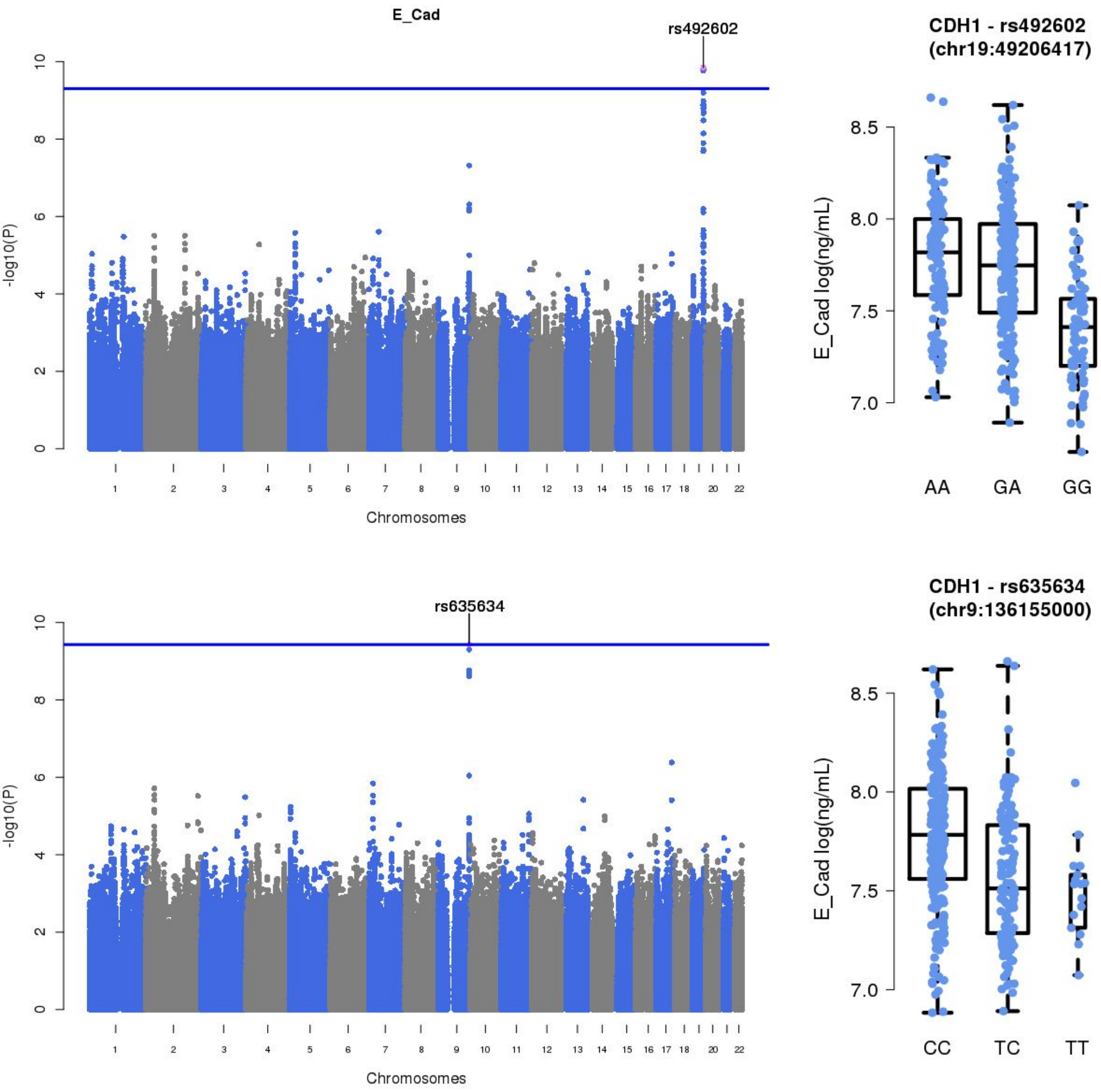

E_Cad
rs492602
-log10(P)
Chromosomes
CDH1 - rs492602
(chr19:49206417)
E_Cad log(ng/mL)
AA
GA
GG
rs635634
-log10(P)
Chromosomes
CDH1 - rs635634
(chr9:136155000)
E_Cad log(ng/mL)
CC
TC
TT

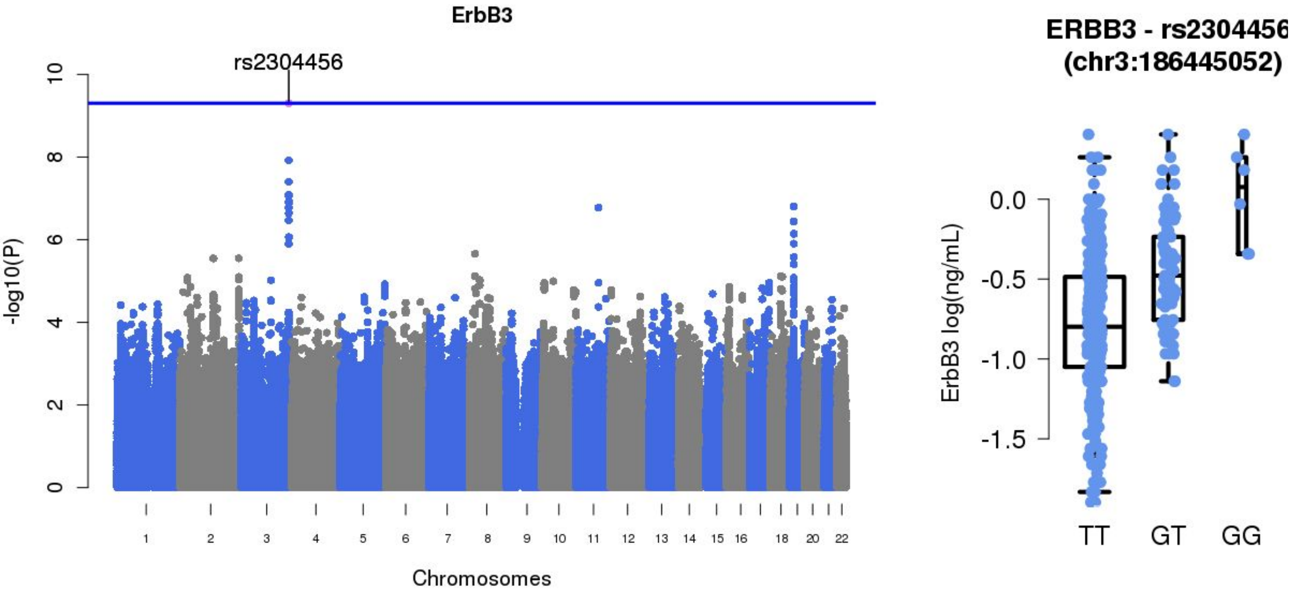

ErbB3
rs2304456
-log10(P)
0
2
4
6
8
10
1
2
3
4
5
6
7
8
9
10
11
12
13
14
15
16
18
20
22
Chromosomes
ERBB3 - rs2304456
(chr3:186445052)
ErbB3 log(ng/mL)
0.0
-0.5
-1.0
-1.5
TT
GT
GG

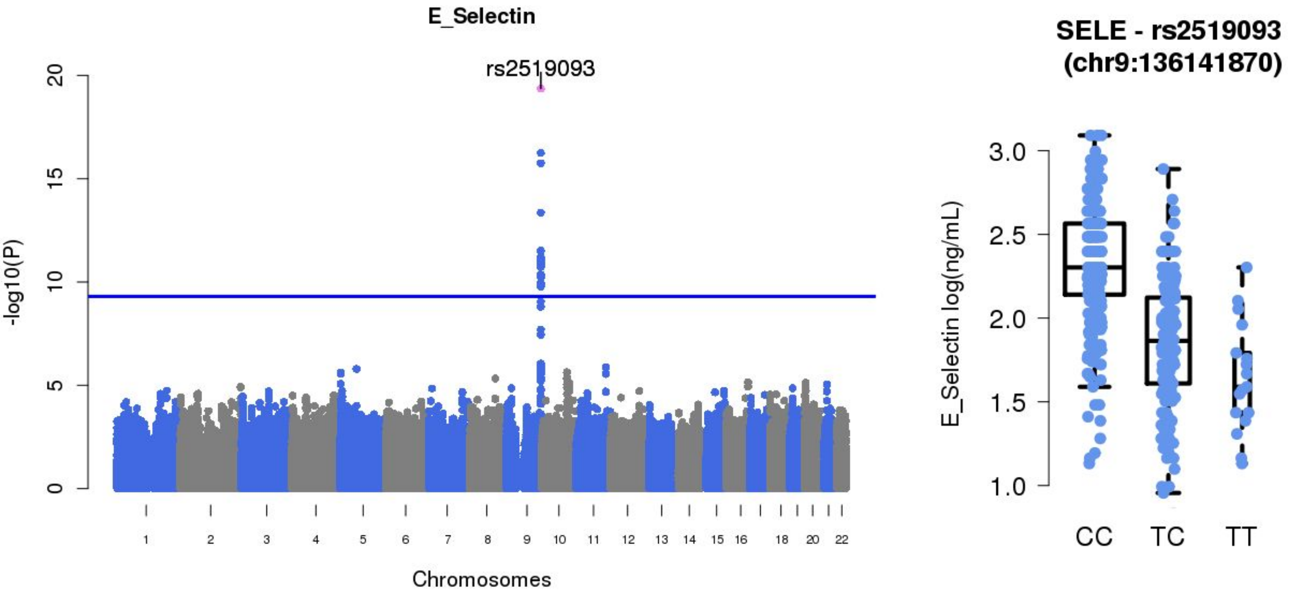
E_Selectin
rs2519093
-log10(P)
0
5
10
15
20
1
2
3
4
5
6
7
8
9
10
11
12
13
14
15
16
18
20
22
Chromosomes
SELE - rs2519093
(chr9:136141870)
E_Selectin log(ng/mL)
1.0
1.5
2.0
2.5
3.0
CC
TC
TT

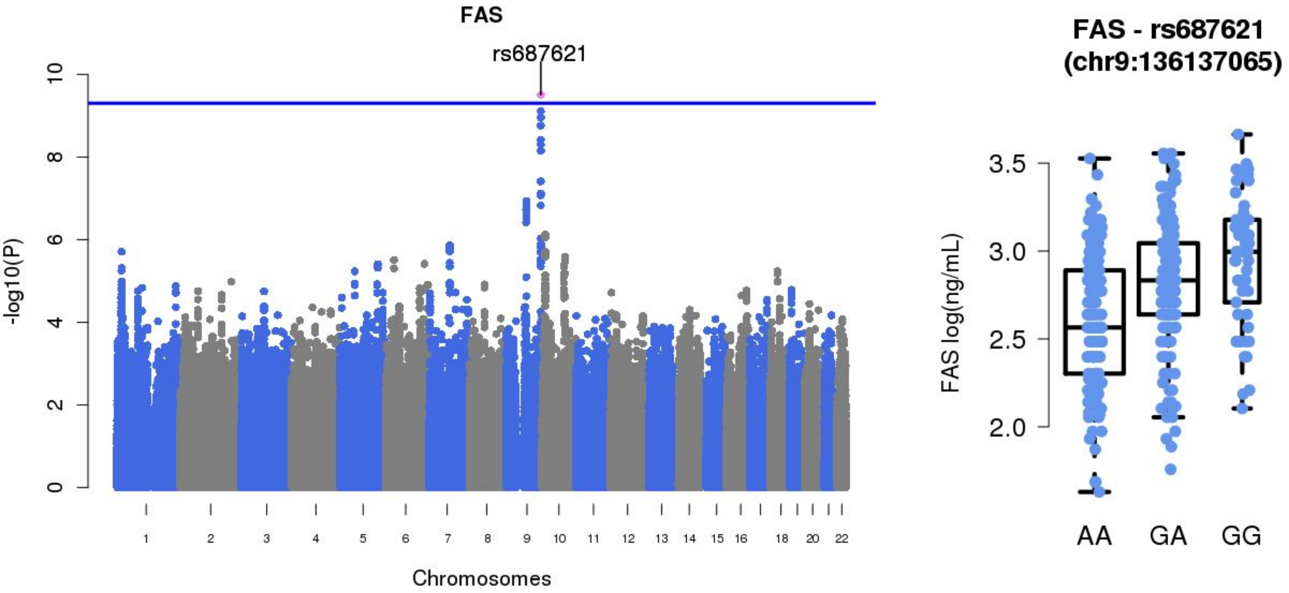

FAS
rs687621
-log10(P)
Chromosomes
1
2
3
4
5
6
7
8
9
10
11
12
13
14
15
16
18
20
22
FAS - rs687621
(chr9:136137065)
FAS log(ng/mL)
3.5
3.0
2.5
2.0
AA
GA
GG

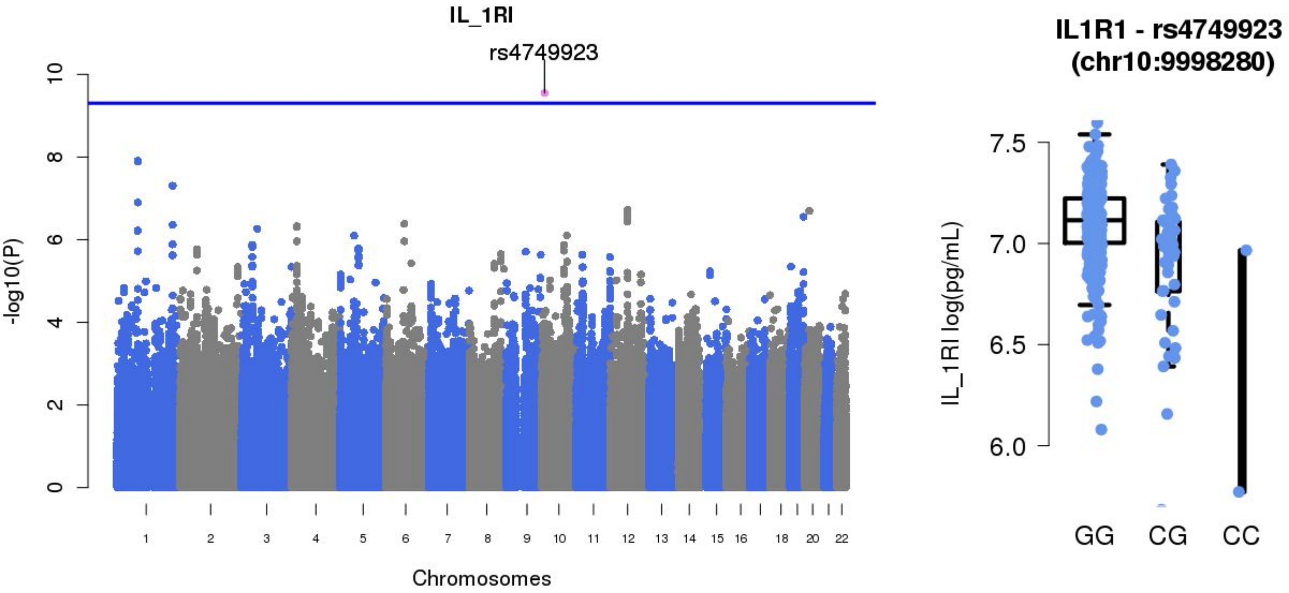
IL_1RI
rs4749923
-log10(P)
Chromosomes
IL1R1 - rs4749923
(chr10:9998280)
IL_1RI log(pg/mL)
GG
CG
CC

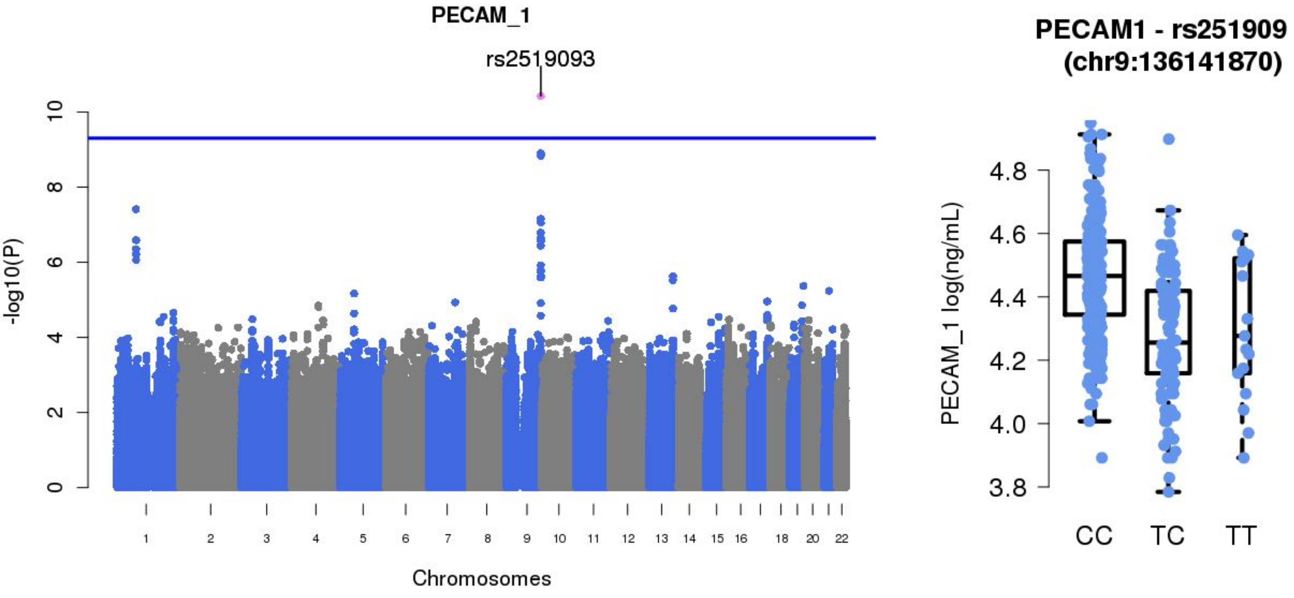
PECAM_1
rs2519093
-log10(P)
0
2
4
6
8
10
1
2
3
4
5
6
7
8
9
10
11
12
13
14
15
16
18
20
22
Chromosomes
PECAM1 - rs251909
(chr9:136141870)
PECAM_1 log(ng/mL)
3.8
4.0
4.2
4.4
4.6
4.8
CC
TC
TT

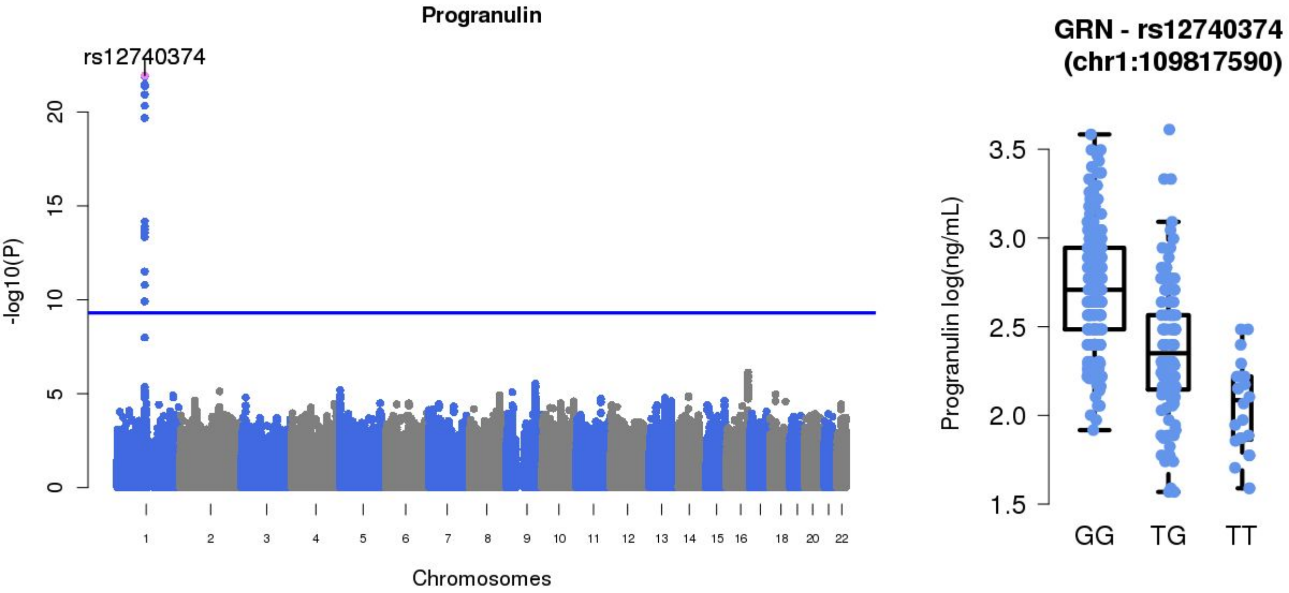

Progranulin
rs12740374
-log10(P)
0
5
10
15
20
1
2
3
4
5
6
7
8
9
10
11
12
13
14
15
16
18
20
22
Chromosomes
GRN - rs12740374
(chr1:109817590)
Progranulin log(ng/mL)
1.5
2.0
2.5
3.0
3.5
GG
TG
TT

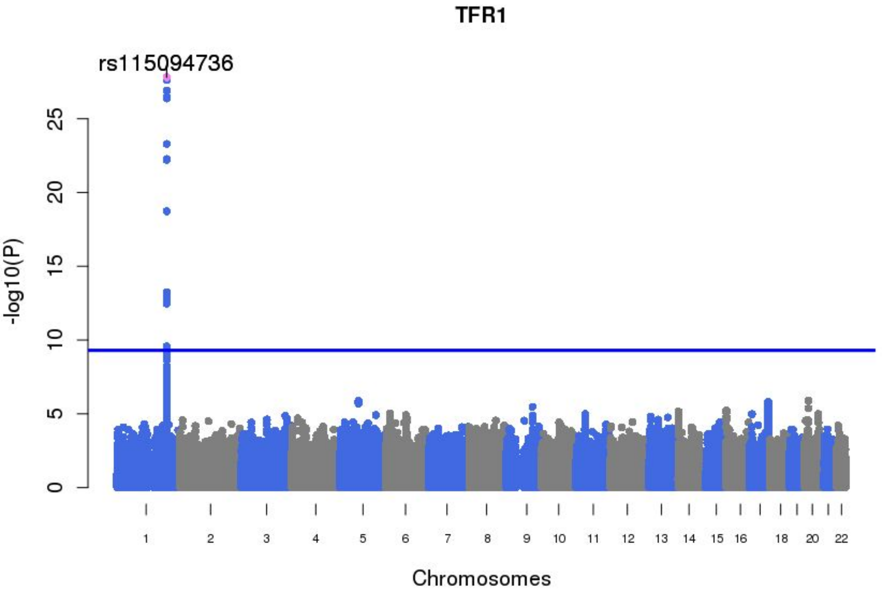
TFR1
rs115094736
-log10(P)
0
5
10
15
20
25
1
2
3
4
5
6
7
8
9
10
11
12
13
14
15
16
18
20
22
Chromosomes

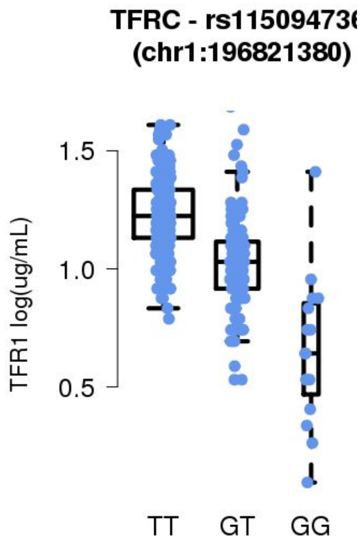
TFRC - rs11509473
(chr1:196821380)
TFR1 log(ug/mL)
1.5
1.0
0.5
TT
GT
GG

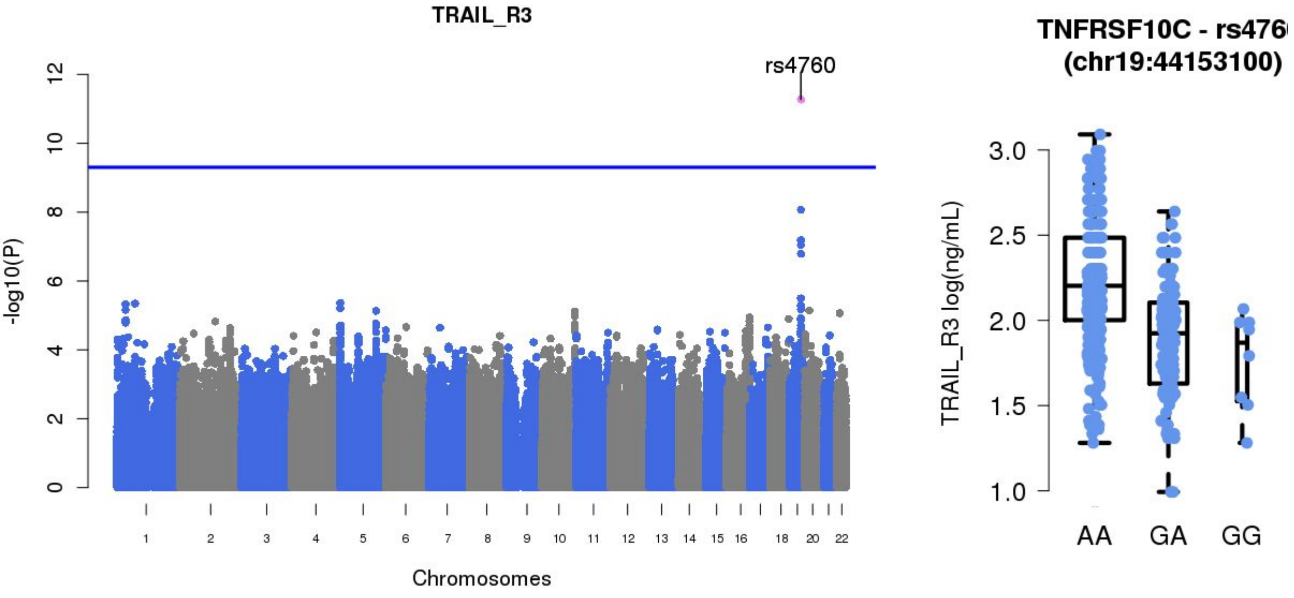

TRAIL_R3
rs4760
-log10(P)
0
2
4
6
8
10
12
1
2
3
4
5
6
7
8
9
10
11
12
13
14
15
16
18
20
22
Chromosomes
TNFRSF10C - rs476
(chr19:44153100)
TRAIL_R3 log(ng/mL)
1.0
1.5
2.0
2.5
3.0
AA
GA
GG

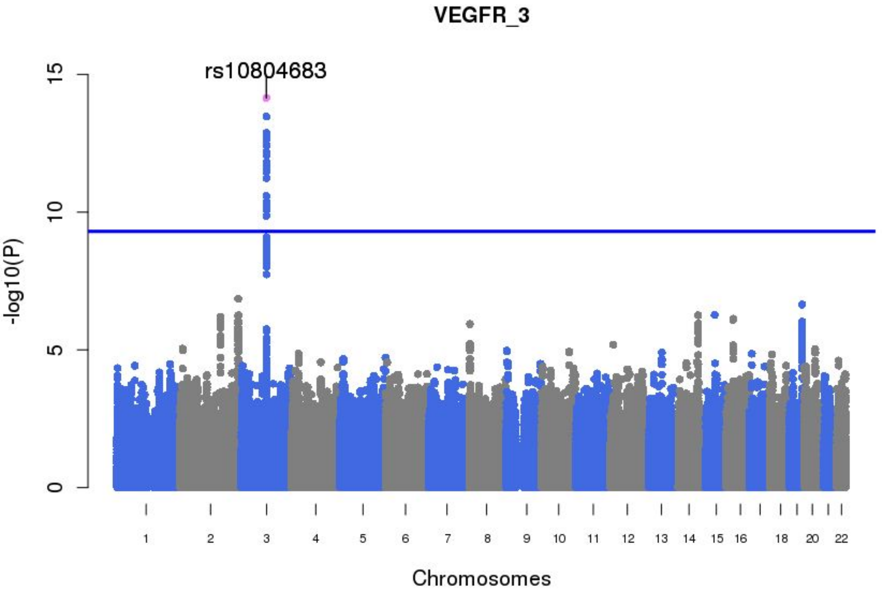
VEGFR_3
rs10804683
-log10(P)
0
5
10
15
1
2
3
4
5
6
7
8
9
10
11
12
13
14
15
16
18
20
22
Chromosomes

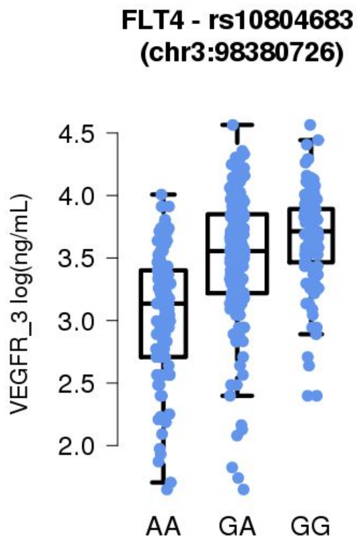
FLT4 - rs10804683
(chr3:98380726)
VEGFR_3 log(ng/mL)
2.0
2.5
3.0
3.5
4.0
4.5
AA
GA
GG

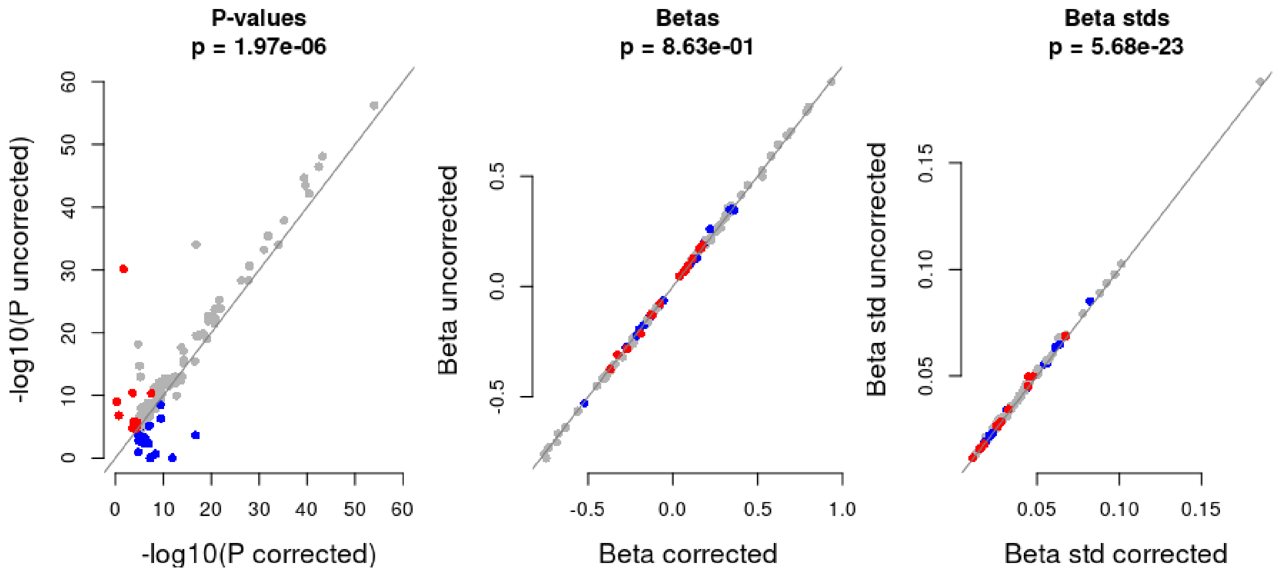

Figure S4: Impact of blood-cell fractions on associated protein-SNP pairs statistics.

The figure shows the distribution of p-values (left), Beta effect size estimates (center) and Beta standard deviation (right) of the 20 protein-SNP hits (blue dots) specific to the setting accounting for cell fractions, the 23 hits (red dots) specific to the setting not accounting for cell fractions, and the 92 hits (grey dots) common to both settings. Summary statistics corresponding to the setting accounting for blood-cell fractions are displayed on the x-axis, while the corresponding statistics not-accounting for blood cell fractions are displayed on the y-axis. Two-sided paired Wilcoxon test p-values comparing the distribution of the statistics across the 135 protein-SNP pairs are shown on the top of each plot. The Spearman rank correlation was high for the three statistics (rho = 0.77, 0.99 and 0.99 for p-values, Beta and Beta std, respectively).
